# Scalable RIS-aided hybrid beamforming for mmWave systems enables multiple sub-array architectures: A Geometric Mean Approach

**DOI:** 10.1371/journal.pone.0354965

**Published:** 2026-08-21

**Authors:** Hoang M. Tran, Tung V. Dinh, Hoa T. Nguyen, Huy T. Nguyen

**Affiliations:** 1 School of Electrical and Electronic Engineering, Hanoi University of Science and Technology, Hanoi, Vietnam; 2 Faculty of Mathematics and Informatics, Hanoi University of Science and Technology, Hanoi, Vietnam; 3 Institute of Physics, Vietnam Academy of Science and Technology, Giang Vo, Hanoi, Vietnam; 4 Faculty of Information Technology, Van Lang School of Technology, Van Lang University, Ho Chi Minh City, Vietnam; Posts and Telecommunications Institute of Technology, VIET NAM

## Abstract

This paper investigates a reconfigurable intelligent surface (RIS)-aided hybrid beamforming (HBF) framework for downlink multi-user millimeter-wave (mmWave) systems operating under practical hardware constraints. Given the severe path-loss disparity and blockage sensitivity inherent in mmWave propagation, RIS-assisted systems often suffer from pronounced user-rate imbalance, a challenge further exacerbated by the restricted spatial degrees of freedom in overlapped sub-array (OSA) hybrid architectures. To explicitly mitigate this disparity, we formulate a hardware-constrained optimization problem to maximize the geometric mean (GM) of user rates, thereby enforcing instantaneous fairness and service reliability. At the base station, an OSA-based HBF architecture is adopted to balance spatial flexibility and RF-chain efficiency, while the RIS operates with discrete phase shifts. Distinctive from conventional sum-rate-driven or idealized fully-digital designs, the proposed framework integrates GM-rate maximization with a manifold-theoretic optimization approach, enabling a realizable mapping from the fully-digital benchmark to a practical hybrid analog-digital structure. A two-block block coordinate descent (BCD) algorithm is developed, wherein the active precoder update admits an exact power-constrained solution and the passive phase optimization is executed on a Riemannian manifold to handle unit-modulus and discrete constraints. Numerical simulations demonstrate that the proposed approach effectively exploits OSA connectivity to achieve robust fairness across users. Specifically, an interference-aware warm-start strategy significantly accelerates convergence and yields an objective value improvement of up to 18.18% compared with existing methods, confirming both mathematical superiority and practical computational efficiency. Moreover, the results validate that low-resolution (e.g., 3-bit) phase shifters are sufficient to approach continuous-phase performance, underscoring the feasibility of the proposed framework for cost- and energy-efficient RIS-assisted mmWave deployments.

## 1 Introduction

Millimeter-wave (mmWave) communication in the 30–300 GHz band is widely recognized as a cornerstone physical-layer technology for sixth-generation (6G) wireless systems because the availability of large contiguous bandwidth fundamentally enables ultra-high data rates and supports spectrum-intensive services envisioned for future networks [1]. At the network architecture level, 6G is further expected to accommodate massive device connectivity and heterogeneous traffic demands across highly diverse deployment scenarios that extend beyond conventional terrestrial cellular infrastructures [2].

Reconfigurable intelligent surfaces (RISs) have emerged as a key enabler of this paradigm shift by transforming the wireless environment into a programmable entity [3]. Comprehensive surveys have established RIS as a fundamental building block of smart radio environments, capable of reshaping wireless propagation to support emerging 6G services [4,5]. Early RIS research primarily focused on enhancing signal strength and spectral efficiency. More recent studies reposition RISs as multi-functional and reliability-critical platforms that integrate communication, sensing, and wireless power transfer [6,7]. In parallel, advanced RIS architectures, including multi-layer, refracting, and self-powered designs, have been investigated to support satellite-terrestrial and space-air-ground integrated networks [8–10]. For instance, multi-layer stacking RIS receivers have been studied for high-altitude platform (HAP)-enabled simultaneous wireless information and power transfer (SWIPT) networks [8], while self-powered adaptive RIS designs have been considered for secure satellite-terrestrial integration [10]. Refracting RIS has also been investigated for hybrid satellite-terrestrial relay systems [9]. More recently, air-driven seamless and massive access has been discussed for space-air-ground integrated networks, where aerial platforms (e.g., unmanned aerial vehicle (UAV) and HAP tiers) play a coordinating role to enable wide-area continuity of access and large-scale user connectivity across heterogeneous layers [11]. These developments are consistent with broader 6G visions that emphasize scalable and intelligent connectivity across heterogeneous network layers [11].

Despite their promise, RIS-assisted mmWave systems face severe physical-layer challenges. MmWave propagation suffers from high path loss, limited diffraction, and strong sensitivity to blockage, which significantly degrades non-line-of-sight (NLoS) coverage. Recent path-loss measurements and channel modeling studies reveal that RIS-assisted mmWave links exhibit pronounced geometry-dependent attenuation and user-specific disparities, particularly in indoor and outdoor deployments [12,13]. Furthermore, statistical characterizations of RIS-assisted channels demonstrate non-trivial SNR distributions across near- and far-field regimes, which further intensify rate imbalance among users [14]. These model- and measurement-driven observations indicate that channel disparity in RIS-assisted mmWave systems is often structural rather than incidental, making fairness and service reliability first-order design challenges rather than secondary performance metrics.

Existing RIS-assisted multi-user MIMO designs predominantly adopt sum-rate or weighted sum-rate maximization frameworks, often solved via WMMSE, semidefinite relaxation (SDR), or alternating optimization methods [15–17]. Although these throughput-centric approaches improve aggregate spectral efficiency, they systematically favor users with strong composite channels and may severely degrade the service quality of cell-edge or blocked users. This limitation is particularly pronounced in RIS-assisted mmWave systems, where blockage and path-loss disparities are inherently amplified by passive reflection mechanisms. Furthermore, many existing works rely on fully-digital precoding assumptions, which are impractical at mmWave frequencies due to the prohibitive cost and power consumption associated with large numbers of RF chains.

Hybrid beamforming (HBF) architectures mitigate these hardware limitations by reducing RF-chain requirements. Fully-connected (FC) architectures provide high beamforming flexibility at significant hardware cost, whereas non-overlapped sub-array (NOSA) architectures reduce complexity at the expense of spatial degrees of freedom [18–21]. The overlapped sub-array (OSA) architecture generalizes both designs by allowing antenna sharing across RF chains, thereby improving spatial flexibility without incurring the full hardware burden of FC architectures [22,23]. However, when RIS-assisted transmission is combined with OSA-based HBF and discrete phase constraints, conventional sum-rate-driven optimization becomes insufficient to guarantee user fairness or service reliability.

Geometric mean (GM) rate maximization provides a principled mechanism to address this fairness and reliability gap by explicitly balancing individual user rates [24]. Unlike sum-rate objectives, which are insensitive to individual rate collapse, GM-rate penalizes vanishing user rates and enforces balanced performance across all users within a single transmission interval. Moreover, the GM-rate achieves a more favorable trade-off between fairness guarantees and overall performance maximization compared to conventional fairness paradigms, such as the max-min fairness objective or proportional rate constraints. Unlike the max-min objective, which heavily favors fairness at the expense of efficiency, or proportional rate constraints, which incur additional computational complexity, the GM objective offers a tractable and scalable solution that implicitly improves fairness without introducing any associated complex constraints. This property is particularly important in quasi-static RIS-assisted mmWave systems, where limited scheduling diversity and reliance on reflected paths prevent fairness from being achieved through long-term averaging. Under OSA-based hybrid beamforming, GM-rate aligns with reduced spatial degrees of freedom by prioritizing robustness and service continuity over peak throughput.

The above design choices are not independent but are closely connected through both fairness and implementation considerations. While GM-rate maximization addresses the fairness limitations of conventional throughput-oriented designs by preventing rate collapse among users with weak composite channels [24], its practical realization in RIS-assisted mmWave systems requires a hardware-efficient beamforming architecture. The OSA-based hybrid beamforming structure provides an attractive compromise between the beamforming flexibility of fully-connected architectures and the reduced hardware complexity of non-overlapped sub-array designs [22]. However, the resulting system introduces strongly coupled digital precoding, analog beamforming, and RIS phase variables under unit-modulus and discrete phase constraints. These characteristics naturally motivate the use of Riemannian manifold optimization, which enables direct optimization over the feasible set without relying on high-dimensional relaxation methods [25]. Furthermore, because the resulting optimization problem remains highly non-convex and involves multiple coupled variable blocks, a BCD framework is adopted to decompose the original problem into tractable subproblems while preserving monotonic objective improvement during the iterative optimization process [26]. Together, these design choices establish a fairness-oriented, hardware-consistent, and computationally tractable optimization framework for RIS-assisted mmWave communications.

In parallel, artificial intelligence (AI) and deep reinforcement learning (DRL) techniques have been actively explored for RIS and hybrid beamforming optimization [17,27]. Although these data-driven approaches exhibit strong adaptability in complex environments, they typically require large training datasets, lack interpretability, and do not explicitly optimize fairness objectives. Model-based optimization frameworks therefore remain attractive for fairness-critical systems, as they provide transparent performance control, interpretable optimization updates, and direct incorporation of hardware constraints. Accordingly, this work adopts a model-based design philosophy that complements, rather than replaces, AI-based RIS optimization. Table 1 highlights how the proposed framework addresses fairness objectives under practical hardware constraints when compared with classical and AI/DRL-based methods.

**Table 1:** Comparative analysis of HBF optimization frameworks for RIS systems.

Feature	Classical (SDR/WMMSE)	AI/DRL-based	Proposed work
**Representative refs.**	[15,16,18]	[4,17,27]	This work
**Objective**	Aggregate sum-rate	Policy / reward-driven adaptation	**Fairness-oriented GM-rate optimization**
**Complexity**	High (matrix lifting, iterative solvers)	Training-heavy (offline learning + online inference)	**Moderate (BCD + RCG)**
**Hardware realizability**	Idealized / relaxed constraints	Scenario-specific implementations	**OSA-consistent hybrid structure**
**Phase resolution**	Continuous (ideal assumption)	Discrete (heuristic or learned)	**Discrete (optimization-driven)**
**Mechanism**	Analytical optimization	Data-driven policy learning	**Riemannian manifold optimization**
**Data dependency**	None	Training data required	**CSI-only (model-based)**
**Fairness treatment**	Implicit or constraint-based	Reward-dependent	**Explicit GM-rate optimization**

As summarized in Table 1, the proposed framework occupies an intermediate position between conventional optimization-based and learning-based beamforming designs. Compared with SDR- and WMMSE-based approaches, it directly accommodates the OSA hybrid architecture and discrete RIS phase constraints without relying on semidefinite relaxation or matrix lifting, while directly handling the structured constraints imposed by the hardware architecture. Compared with AI- and DRL-based methods, the proposed approach does not require offline training and explicitly incorporates user fairness through GM-rate maximization. Additionally, the proposed framework inherits several limitations common to model-based optimization, including its dependence on CSI availability and convergence to a stationary point of a non-convex problem. Moreover, its iterative nature makes it more suitable for scenarios where channel variations occur on a slower time scale than the optimization process. Therefore, the proposed framework should be viewed as a fairness-oriented and hardware-consistent model-based solution that complements, rather than replaces, existing analytical and learning-based approaches.

Meanwhile, OSA-based RIS hybrid beamforming is particularly suitable for practical mmWave deployments where blockage is a major concern, such as indoor hotspots in shopping malls, airports, and large office buildings. In these scenarios, severe penetration loss and frequent human blockage degrade mmWave links. Compared to fully connected architectures, the OSA structure reduces the number of phase shifters and RF interconnections, thereby lowering hardware complexity and power consumption while maintaining satisfactory data rates. Moreover, the control and calibration overhead remains manageable, as RIS phase updates are performed over a low-rate control link at the channel coherence time scale, and with discrete phase shifts of limited resolution, the signaling payload scales linearly with the number of elements and quantization bits.

Motivated by these observations, this paper investigates a downlink multi-user mmWave system assisted by an RIS, where the base station employs OSA-based hybrid beamforming and the RIS operates with discrete phase shifts, with a particular focus on fairness-oriented joint precoder and phase optimization under practical hardware constraints. The proposed framework directly maximizes the GM-rate to ensure fairness while explicitly accounting for hardware limitations imposed by hybrid architectures and discrete phase control. Unlike fully-digital GM-rate designs [24], this work develops a realizable OSA-consistent hybrid beamforming solution.

The main contributions of this paper are summarized as follows:

1. **Conceptual novelty**: We formulate a fairness-driven GM-rate maximization problem for RIS-assisted mmWave systems with OSA-based hybrid beamforming and discrete RIS phases, thereby extending the applicable configuration concept and explicitly addressing the reliability gap inherent in sum-rate-centric designs prevalent in the existing literature.
2. **Methodological differentiation**: We propose an OSA-consistent Riemannian manifold factorization that maps the fully-digital GM-optimal precoder benchmark into a realizable hybrid analog-digital structure without resorting to matrix lifting or semidefinite relaxation. We apply a dynamic GM-based weighting approximation in BCD that differs from fixed weights in sum-rate formulations. Furthermore, instead of directly applying the Riemannian manifold conjugate descent, we iteratively search for the optimal digital beamforming matrix via a closed-form expression while reformulating the optimal analog beamforming matrix as a manifold factorization problem.
3. **Practical advantages**: We develop an interference-aware warm-start strategy that stabilizes the power-constrained precoder update and accelerates convergence by up to 18.18% compared with random initialization. Extensive simulations demonstrate that OSA-based architectures with moderate RF-chain counts and low-resolution (3-bit) phase shifters at both the BS and RIS can closely approach continuous-phase performance, validating the practicality of the proposed framework.

**Novelty Statement.** To the best of our knowledge, this work represents the first principled attempt to systematically bridge three previously disconnected aspects in RIS-assisted mmWave systems, namely fairness-oriented performance optimization by leveraging a GM objective, practical OSA-based hybrid beamforming architectures, and discrete phase control within a unified Riemannian manifold optimization framework. In terms of the conceptual system model, unlike the prevailing literature that prioritizes sum-rate maximization or relies on idealized fully-digital precoding assumptions [15–17], the proposed framework directly addresses the structural rate imbalance intrinsic to RIS-aided mmWave propagation with the influence of hybrid beamforming, where blockage sensitivity and geometry-dependent attenuation fundamentally limit user-level reliability [12,13]. By integrating GM-rate maximization with an OSA-consistent hybrid realization under discrete phase constraints, this work fills a critical research gap and paves a realizable methodology toward fairness-critical RIS-assisted deployments under stringent hardware limitations [24].

This journal article substantially extends the authors’ preliminary [28] through several conceptual and methodological advancements rather than incremental refinements. Specifically, the journal version introduces: (i) a refined fairness-oriented optimization formulation that explicitly targets user-rate imbalance instead of aggregate throughput maximization, which was not considered in the conference version [24]; (ii) a novel OSA-consistent Riemannian manifold factorization mechanism that rigorously maps the fully-digital optimum into a realizable hybrid analog-digital structure with discrete phase constraints [22,23]; and (iii) an explicit theoretical and practical positioning with respect to emerging AI- and DRL-based RIS optimization approaches [17,27]. Collectively, these extensions establish a more comprehensive and tightly grounded optimization framework with enhanced system-level relevance beyond the scope of the original conference contribution.

## 2 System model and problem formulation

### 2.1 System mModel

**Fig 1:**
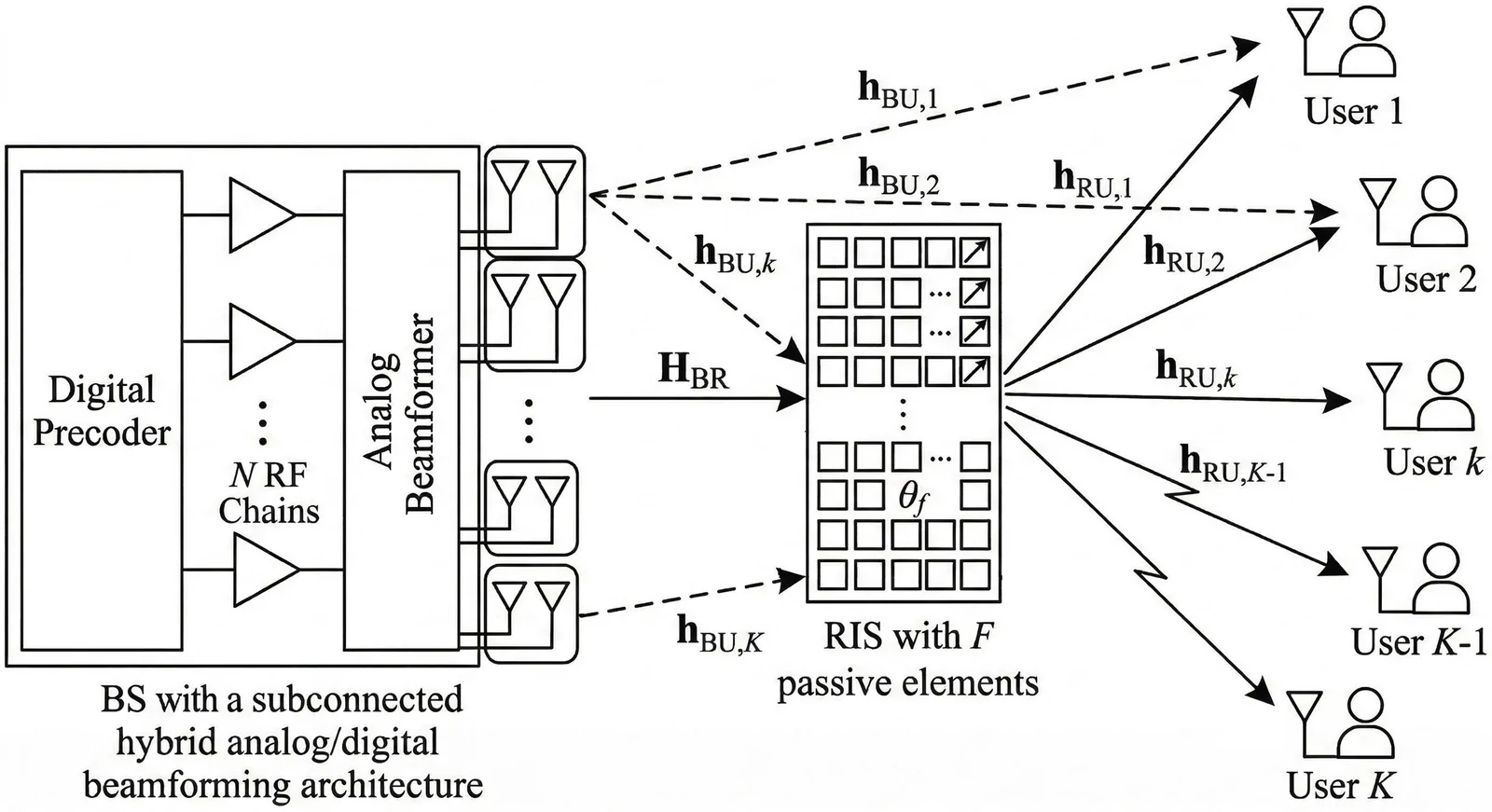
System model of the RIS-assisted hybrid beamforming (HBF) mmWave communication system.

We consider an RIS-assisted downlink millimeter-wave (mmWave) communication system, where a single base station (BS) equipped with *M* antennas serves *K* single-antenna users with the aid of one RIS comprising *F* passive reflecting elements, as illustrated in Fig 1. The BS adopts a subconnected hybrid analog/digital (A/D) beamforming architecture with *N* RF chains, where each RF chain is connected to a subset of $M_{S}$ consecutive antennas with $M_{S}\leq M$. This subarray-based structure effectively reduces hardware complexity while preserving sufficient beamforming flexibility. Due to the severe blockage and scattering characteristics of mmWave propagation, the direct BS–user links are assumed to be weak and are modeled as scattered paths, while the RIS establishes a dominant reflected link to enhance the received signal quality at the users. The Table 2 summarizes all key symbols, including variables, vectors, matrices, and sets, together with their dimensions and concise descriptions.

**Table 2:** List of symbols and notations.

Symbol	Dimension / Set	Description
k∈𝒦	{1,...,K}	User index and user set
n∈𝒩	{1,...,N}	RF-chain index
f∈ℱ	{1,...,F}	RIS element index
κ	${\mathbb{Z}}_{+}$	Iteration index of BCD optimization
η	${\mathbb{Z}}_{+}$	Iteration index of hybrid beamforming factorization
*M*	${\mathbb{Z}}_{+}$	Number of BS antennas
*N*	${\mathbb{Z}}_{+}$	Number of RF chains
*F*	${\mathbb{Z}}_{+}$	Number of RIS reflecting elements
*P*_0_	${\mathbb{R}}_{+}$	Maximum transmit power at the BS
$\sigma_{k}^{2}$	${\mathbb{R}}_{+}$	Noise variance at user *k*
${𝐰}_{k}$	${\mathbb{C}}^{N\times 1}$	Digital beamforming vector for user *k*
𝐖	${\mathbb{C}}^{N\times K}$	Digital beamforming matrix
𝐕	${\mathbb{C}}^{M\times N}$	Analog beamforming matrix with OSA structure
Θ	${\mathbb{C}}^{F\times F}$	RIS reflection coefficient matrix
$b_{f}$	ℂ	Reflection coefficient of RIS element *f*
𝐆	${\mathbb{C}}^{F\times M}$	BS-RIS channel matrix
${𝐡}_{k}$	${\mathbb{C}}^{F\times 1}$	RIS-user *k* channel vector
$R_{k}$	${\mathbb{R}}_{+}$	Achievable rate of user *k*
𝐐	${\mathbb{C}}^{M\times K}$	Fully-digital equivalent precoder
G(·)	${\mathbb{R}}_{+}$	Geometric mean (GM) rate objective
$\rho_{k}^{\left(\kappa \right)}$	${\mathbb{R}}_{+}$	GM linearization weight for user *k* at iteration κ
μ	${\mathbb{R}}_{+}$	Lagrange multiplier associated with the BS transmit power constraint
$\mu_{k}^{\left(\kappa \right)}$	${\mathbb{R}}_{+}$	SINR-related auxiliary variable for user *k* at iteration κ
V	Manifold	Feasible manifold of unit-modulus analog beamforming coefficients

Let $s_{k}$ be the complex-valued information symbol designated for user *k*, where k∈𝒦={1,2,...,K}. We assume that the signals $\left\{s_{k}\right\}$ are mutually independent and normalized to unit power, such that $𝔼[|s_{k}{|}^{2}]=1$ for all k∈𝒦. Initially, each signal $s_{k}$ is weighted by a digital beamforming vector ${𝐰}_{k}\in {\mathbb{C}}^{N\times 1}$ to map the signal onto *N* RF chains. These digitally precoded signals are then summed across all users. This process results in a composite baseband signal vector of dimension N×1. The composite baseband signal, formed by digital precoding across *K* users, is subsequently processed by an analog beamforming matrix $𝐕=[{𝐯}_{1},{𝐯}_{2},...,{𝐯}_{N}]\in {\mathbb{C}}^{M\times N}$, where column ${𝐯}_{n}$ represents the analog precoder associated with RF chain *n*, for n∈𝒩={1,2,...,N}. This analog precoding stage maps the RF-chain outputs to the physical antenna elements. Under the subconnected architecture, each RF chain is connected to a subset of $M_{S}$ consecutive antennas. Accordingly, the analog beamforming vector ${𝐯}_{n}$ contains $M_{S}$ nonzero entries, denoted by ${\tilde{v}}_{n}(m)$ for $m\in {ℳ}_{S}=\{1,2,...,M_{S}\}$, corresponding to the antennas assigned to RF chain *n*. Each ${\tilde{v}}_{n}(m)$ is implemented via a phase shifter that applies a fixed-magnitude complex weight to the signal, satisfying $|{\tilde{v}}_{n}(m)|=1$.

In this work, we assume discrete phase control for all analog coefficients. The set of allowable phase shifts is defined as


$$\begin{array}{c} {𝒮}_{a}=\left\{e^{j\theta }\;|\;\theta \in \left\{0,\frac{2\pi }{2^{Q_{1}}},...,\frac{2\pi (2^{Q_{1}}-1)}{2^{Q_{1}}}\right\}\right\}, \end{array}$$ (1)

where *Q*_1_ denotes the number of control bits per phase shifter. In the special case where $Q_{1}=\infty$, the phase shifts become continuous over [0,2π).

The structure of 𝐕 depends on the array architecture. In the subconnected case, each column contains exactly $M_{S}$ non-zero entries corresponding to the antennas connected to its RF chain, while all other entries are zero. The structure of 𝐕 is illustrated as


$$\begin{array}{c} 𝐕=[\begin{array}{cccc} {\tilde{v}}_{1}(1) & & & \\ \vdots & {\tilde{v}}_{2}(1) & & \\ {\tilde{v}}_{1}(M_{S}) & \vdots & \ddots & {\tilde{v}}_{N}(1)\\ & {\tilde{v}}_{2}(M_{S}) & \cdots & \vdots \\ & & & {\tilde{v}}_{N}(M_{S}) \end{array}], \end{array}$$ (2)

where ${\tilde{v}}_{n}(m)$ recalls the complex-valued phase shift applied to antenna *m* by RF chain *n*, for $m\in {ℳ}_{S}=\{1,2,...,M_{S}\}$. All non-zero entries satisfy the unit-modulus constraint, meaning $|{\tilde{v}}_{n}(m)|=1$, and are chosen from a discrete phase set ${𝒮}_{a}$.

For the general OSA design, 𝐕 features vectors that contain shifted zeros of size $\Delta M_{S}$. This design naturally encompasses two special cases: the FC case, where the constraint is $\Delta M_{S}=0$, $M_{S}=M$, and the NOSA case, where $\Delta M_{S}=M_{S}=M/N$. As a result, 𝐕 has no zeros or becomes a block diagonal, respectively. Consequently, the total transmit power of the BS is given by


$$\begin{array}{c} P_{BS}\;=\;\sum_{k=1}^{K}\|𝐕{𝐰}_{k}{\|}^{2}. \end{array}$$ (3)

The RIS is connected to the BS through an RIS control link for transmission and information exchange. Denote ℱ={1,2,...,F} the set of all RIS unit cells. The response matrix at the RIS is formulated by


$$\begin{array}{c} \Theta =\mathrm{diag}(b_{1},b_{2},...,b_{F}), \end{array}$$ (4)

where $b_{f}=\beta_{f}e^{j\theta_{f}},\beta_{f}\in [0,1]$ and $\theta_{f}\in [0,2\pi )$ are the amplitude reflection coefficient and the phase shift of the *f*-th unit cell, respectively. Since we adopt the passive RIS configuration, we have $\beta_{f}=1$, ∀f∈ℱ. Denote ${𝒮}_{r}$ as the set of all possible phase shifts for the RIS reflection coefficients, given by:


$$\begin{array}{c} {𝒮}_{r}=\left\{e^{j\theta }\;|\;\theta \;\in \left\{0,\;\frac{2\pi }{2^{Q_{2}}},...,\frac{2\pi \left(2^{Q_{2}}-1\right)}{2^{Q_{2}}}\right\}\;\right\}, \end{array}$$ (5)

where *Q*_2_ is the number of control bits for each RIS element. The special case of $Q_{2}=\infty$ corresponds to the continuous phase shifts. We consider the scenario that the BS-user direct link is blocked, and thus the direct path can be ignored. The signal power reflected two or more times is much lower than that reflected just once due to the high free-space path loss. Thus, we ignore the power of the signals that are reflected by the RIS more than once. In addition, we consider that full channel state information (CSI) is available at the BS, and all channels experience quasi-static flat-fading. It is important to highlight that the assumption of CSI at the base station is widely used in HBF for mmWave communication studies [20,24,29], as it enables tractable optimization and serves as a benchmark for performance evaluation. In this work, CSI refers to either perfect or reliably estimated values obtained through standard pilot-based training methods [30], which are practical in slow-fading or quasi-static conditions. Although this assumption simplifies the analysis, it provides meaningful upper bounds for system design. Suppose that $𝐆\in {\mathbb{C}}^{F\times M}$ is the channel matrix from BS to RIS, ${𝐡}_{k}^{H}\in {\mathbb{C}}^{1\times F}$ is the channel vector from RIS to user *k*. Then the signal received by the user *k* can be represented as


$$\begin{array}{c} y_{k}={𝐡}_{k}^{H}\Theta GV\sum_{j=1}^{K}{𝐰}_{j}s_{j}+n_{k},\;\forall k\in K, \end{array}$$ (6)

where $n_{k}\sim CN(0,\sigma_{k}^{2})$ is the additive white Gaussian noise at the receiver of user *k* with zero mean and variance $\sigma_{k}^{2}$. The achievable rate of user *k* can be expressed as:


$$\begin{array}{c} R_{k}=\log_{2}(1+{SINR}_{k}), \end{array}$$ (7)

where ${SINR}_{k}$ is the signal-to-interference-plus-noise ratio (SINR) at the *k*-th user, which can be calculated by


$$\begin{array}{c} {SINR}_{k}=\frac{{\left|{𝐡}_{k}^{H}\Theta 𝐆𝐕{𝐰}_{k}\right|}^{2}}{\sum_{j\neq k}{\left|{𝐡}_{k}^{H}\Theta 𝐆𝐕{𝐰}_{j}\right|}^{2}+\sigma_{k}^{2}},\;\forall k\in 𝒦. \end{array}$$ (8)

### 2.2 mmWave channel model

We adopt the widely used narrowband clustered channel model for mmWave communications. Specifically, the channel matrix between the BS and the RIS can be written as [20]:


$$\begin{array}{c} 𝐆=\sqrt{\frac{MF}{N_{cl_{1}}N_{ray_{1}}}}\sum_{i=1}^{N_{cl_{1}}}\sum_{l=1}^{N_{ray_{1}}}\alpha_{il}{𝐚}_{R}(\phi_{il}^{Rr},\delta_{il}^{Rr}){𝐚}_{B}(\phi_{il}^{B},\delta_{il}^{B}{)}^{H}. \end{array}$$ (9)

Here, $N_{cl_{1}}$ denotes the number of scattering clusters, $N_{ray_{1}}$ denotes the number of rays in each cluster, and $\alpha_{il}$ denotes the channel coefficient of the *l* -th ray in the *i* -th propagation cluster. Moreover, ${𝐚}_{R}(\phi_{il}^{Rr},\delta_{il}^{Rr})$ and ${𝐚}_{B}(\phi_{il}^{B},\delta_{il}^{B})$ represent the receive array response vectors of the RIS and the transmit array response vectors of the BS, respectively, with $\phi_{il}^{Rr}(\phi_{il}^{B})$ and $\delta_{il}^{Rr}(\delta_{il}^{B})$ representing azimuth and elevation angles of arrival at the RIS (or departing from the BS). The channe*l* vector between the RIS and the *k* -th user can be represented as


$$\begin{array}{c} {𝐡}_{k}=\sqrt{\frac{F}{N_{cl2}N_{ray2}}}\sum_{i=1}^{N_{cl2}}\sum_{l=1}^{N_{ray2}}\beta_{il}{𝐚}_{R}(\phi_{il}^{Rt},\delta_{il}^{Rt}). \end{array}$$ (10)

Here, $N_{cl2}$, $N_{ray2}$, $\beta_{il}$, $\phi_{il}^{Rt}$, and $\delta_{il}^{Rt}$ are defined similarly as above. We assume that the uniform planar array (UPA) structure is adopted at both BS and RIS. Consequently, the array response vector can be denoted as:


$$\begin{array}{c} {𝐚}_{z}(\phi ,\delta )=\frac{1}{\sqrt{A_{1}A_{2}}}[\begin{array}{c} 1\\ \vdots \\ e^{j\frac{2\pi }{\lambda }d_{1}(o\sin\phi \sin\delta +p\cos\delta )}\\ \vdots \\ e^{j\frac{2\pi }{\lambda }d_{1}\left[(A_{1}-1)\sin\phi \sin\delta +(A_{2}-1)\cos\delta \right]} \end{array}], \end{array}$$ (11)

where z∈{R,B}, λ is the signal wavelength, *d* is the antenna or unit cell spacing which is assumed to be half a wavelength, $0\leq o<A_{1}$ and $0\leq p<A_{2}$. *A*_1_ and *A*_2_ represent the number of rows and columns of the UPA in the 2D plane, respectively.

### 2.3 Problem formulation

Existing works in the literature often use the sum-rate method and the max-min method to quantify the quality of service (QoS) of the network, each of the formulation in favor of the overall rates or resource fairness across users, respectively. To balance this trade-off between total QoS and resource fairness, a proportional fairness constraint is introduced as an alternative method, at the cost of additional complexity of non-convex constraints [31]. Thus, the recent GM rate maximization method overcomes this challenge by considering a different objective function [24]. Our target is to tackle the following problem by jointly optimizing the digital beamforming matrix $𝐖=[{𝐰}_{1},{𝐰}_{2},...,{𝐰}_{K}]\in {𝒞}^{N\times K}$ and the analog beamforming matrix 𝐕 at the BS, as well as the overall response matrix Θ at the RIS, to maximize the geometric mean of users’ rates:


$$\begin{array}{cc} \max_{𝐖,𝐕,\Theta }\; & \prod_{k=1}^{K}(R_{k}(𝐖,𝐕,\Theta ){)}^{\frac{1}{K}} \end{array}$$ (12a)

$$\begin{array}{cc} \text{s.t.}\; & \sum_{k=1}^{K}\|𝐕{𝐰}_{k}{\|}^{2}\leq P_{0}, \end{array}$$ (12b)

$$\begin{array}{cc} & v_{n,d}\in {𝒮}_{a},\;\forall n\in 𝒩,\forall d\in 𝒟, \end{array}$$ (12c)

$$\begin{array}{cc} & b_{f}\in {𝒮}_{r},\;\forall f\in ℱ. \end{array}$$ (12d)

From problem (12), we can observe that the GM rate maximization problem is highly non-convex due to the nature of the geometric mean objective function (12a) and the coupling between the three variable matrices of the digital beamformer, RIS reflection coefficient, and the discrete phase shifters of the analog beamformer (12c). To circumvent the difficulty of finding a global optimal solution, we instead adopt a sub-optimal approach using the BCD method. This involves iteratively solving a relaxed version of (12) w.r.t. one variable matrix at a time, while keeping all others fixed.

## 3 RIS-assisted hybrid beamforming solution for GM rate optimization

In this section, we introduce an efficient methodology to handle the GM rate maximization problem (12) leveraging *two-block* iterative algorithms. Specifically, we first handle the discrete phase shifts by adopting the projection method that relaxes the discrete phase shifts of analog beamforming and RIS coefficients to continuous ones, then address the relaxed problem with the proposed algorithms, and project the obtained continuous solution back to the discrete set. We introduce an auxiliary variable $𝐐=[{𝐪}_{1},{𝐪}_{2},...,{𝐪}_{K}]$ with ${𝐪}_{i}=𝐕{𝐰}_{i},\forall i\in 𝒦$ and solve (12) iteratively w.r.t. 𝐐 and Θ, respectively while keeping the other fixed. Thus, we consider a relaxation version of (12) as


$$\begin{array}{cc} \max_{𝐐,\Theta }\; & G(𝐐,\Theta )=\prod_{k=1}^{K}(R_{k}(𝐐,\Theta ){)}^{\frac{1}{K}} \end{array}$$ (13a)

$$\begin{array}{cc} \text{s.t.}\; & \sum_{k=1}^{K}\|{𝐪}_{k}{\|}^{2}\leq P_{0}, \end{array}$$ (13b)

$$\begin{array}{cc} & \|b_{f}\|=1,\;\forall f\in ℱ. \end{array}$$ (13c)

We note that the maximize the objective function G(𝐐,Θ) of (13) is equivalent to minimize 1/G(𝐐,Θ) subject to (13b) and (13c). Notice that the function 1/G(𝐐,Θ) is the composition of the convex function $f(R_{1},...,R_{K})=1/(\prod_{k=1}^{K}R_{k}{)}^{1/K}$ and the non-convex functions $R_{k}(𝐐,\Theta ),\;k=1,...,K$. We then consider the linearization of 1/G(𝐐,Θ) around $\left({𝐐}^{\left(\kappa \right)},\Theta^{\left(\kappa \right)}\right)$, a feasible point that is found from the (κ−1) iteration. Thus, we apply a first-order Taylor approximation at each BCD iteration κ, transforming problem (13) into the form $2f\;\left(R_{1}^{\left(\kappa \right)},...,R_{K}^{\left(\kappa \right)}\right)-f\;\left(R_{1}^{\left(\kappa \right)},...,R_{K}^{\left(\kappa \right)}\right)\frac{1}{K}\sum_{k=1}^{K}\frac{R_{k}(𝐐,\Theta )}{R_{k}^{\left(\kappa \right)}},$ where $R_{k}^{\left(\kappa \right)},\;k=1,...,K$ denotes the rate of user *k* evaluated at $\left({𝐐}^{\left(\kappa \right)},\Theta^{\left(\kappa \right)}\right)$. Since we have $f\;\left(R_{1}^{\left(\kappa \right)},...,R_{K}^{\left(\kappa \right)}\right)>0$, we can generate the next feasible point $\left({𝐐}^{\left(\kappa +1\right)},\Theta^{\left(\kappa +1\right)}\right)$ from the following equivalent problem:


$$\begin{array}{cc} \max_{𝐐,\Theta }\; & \hat{G}(𝐐,\Theta )≜\sum_{k=1}^{K}\rho_{k}^{\left(\kappa \right)}R_{k}(𝐐,\Theta ) \end{array}$$ (14a)

$$\begin{array}{cc} \text{ s.t. } & \;\text{(13b)},\text{(13c)}, \end{array}$$ (14b)

where $\rho_{k}^{\left(\kappa \right)}≜1/(G^{\left(\kappa \right)}R_{k}^{\left(\kappa \right)}),\forall k\in 𝒦$. Here, $G^{\left(\kappa \right)}$ and $R_{k}^{\left(\kappa \right)}$ are the objective function of (13) and the achievable rate of user *k*, evaluated at $\left({𝐐}^{\left(\kappa \right)},\Theta^{\left(\kappa \right)}\right)$ – the feasible point of (13) generated by iteration κ, respectively. This first-order Taylor approximation reveals a key distinction between the two optimization approaches: in the maximizing sum rate problem, the weight coefficients $\rho_{k}$ remain fixed within each iteration, whereas in the geometric mean approach, the coefficients $\rho_{k}$ are dynamically modified after every variable update.

Moreover, we note that the linearized problem (14) is still not a convex optimization problem since the matrices (𝐐 and Θ) are still coupled in the objective function (14a) while the feasible domain remains non-convex due to the unit-modulus constraint (13c) of passive RIS. This motivates us to employ the BCD technique to decouple the optimization variables. Specifically, the overall optimization process, κ, is broken down into two sequential sub-iterations consisting of the FD beamforming iteration and the RIS phase update iteration.

### 3.1 FD beamforming iteration

At each FD Beamforming iteration κ+1, we generate a matrix ${𝐐}^{\left(\kappa +1\right)}$ such that the objective function improves: $\hat{G}({𝐐}^{\left(\kappa +1\right)},\Theta^{\left(\kappa \right)})>\hat{G}({𝐐}^{\left(\kappa \right)},\Theta^{\left(\kappa \right)})$. Following [32], the below inequality holds for all 𝐐 and Θ


$$\begin{array}{c} R_{k}\left(𝐐,\Theta^{\left(\kappa \right)}\right)\geq \;x_{k}^{\left(\kappa \right)}+2ℜ\left\{\left\langle y_{k}^{\left(\kappa \right)},{𝐪}_{k}\right\rangle \right\}-z_{k}^{\left(\kappa \right)}\sum_{j=1}^{K}{\left|{ℋ}_{k}^{\left(\kappa \right)}{𝐪}_{j}\right|}^{2}, \end{array}$$ (15)

with ${ℋ}_{k}^{\left(\kappa \right)}$, $x_{k}^{\left(\kappa \right)}$, $y_{k}^{\left(\kappa \right)}$ and $z_{k}^{\left(\kappa \right)}$ are determined by ${ℋ}_{k}^{\left(\kappa \right)}≜{𝐡}_{k}^{H}\Theta^{\left(\kappa \right)}𝐆$, $x_{k}^{\left(\kappa \right)}≜R_{k}\left({𝐐}^{\left(\kappa \right)},\Theta^{\left(\kappa \right)}\right)-\left({\left|{ℋ}_{k}^{\left(\kappa \right)}q_{k}^{\left(\kappa \right)}\right|}^{2}\right)/\mu_{k}^{\left(\kappa \right)}-\sigma_{k}^{2}z_{k}^{\left(\kappa \right)}$, $y_{k}^{\left(\kappa \right)}≜\left({\left({ℋ}_{k}^{\left(\kappa \right)}\right)}^{H}{ℋ}_{k}^{\left(\kappa \right)}q_{k}^{\left(\kappa \right)}\right)/\mu_{k}^{\left(\kappa \right)}$, $\mu_{k}^{\left(\kappa \right)}≜\sum_{j\neq k}{\left|{ℋ}_{j}^{\left(\kappa \right)}{𝐪}_{j}^{\left(\kappa \right)}\right|}^{2}+\sigma_{k}^{2}$, and $z_{k}^{\left(\kappa \right)}≜\left({\left|{ℋ}_{k}^{\left(\kappa \right)}q_{k}^{\left(\kappa \right)}\right|}^{2}\right)/\left(\mu_{k}^{\left(\kappa \right)}\left(\mu_{k}^{\left(\kappa \right)}+{\left|{ℋ}_{k}^{\left(\kappa \right)}q_{k}^{\left(\kappa \right)}\right|}^{2}\right)\right)$.

The lower bound of function $R_{k}\left(𝐐,\Theta^{\left(\kappa \right)}\right)$ given by (15) is now concave quadratic. Thus, we can replace each user’s rate $R_{k}$ in (14a) with its lower-bound counterpart. By using the Lagrangian multiplier method, we can derive the following closed-form solution of the FD Beamforming iteration as follows:


$$\begin{array}{c} {𝐐}^{\left(\kappa +1\right)}=\left\{ \begin{array}{cc} {\left(\Psi^{\left(\kappa \right)}\right)}^{-1}\rho_{k}^{\left(\kappa \right)}{𝐲}^{\left(\kappa \right)}, & \text{if }{\left\|{𝐐}^{\left(\kappa +1\right)}\right\|}^{2}\leq P\\ {\left(\Psi^{\left(\kappa \right)}+\mu I_{M}\right)}^{-1}\rho_{k}^{\left(\kappa \right)}{𝐲}^{\left(\kappa \right)}, & \text{otherwise} \end{array}\right. \end{array}$$ (16)

where ${𝐲}^{\left(\kappa \right)}≜[y_{1}^{\left(\kappa \right)},y_{2}^{\left(\kappa \right)},...,y_{K}^{\left(\kappa \right)}{]}^{T}$, $\Psi^{\left(k\right)}≜\sum_{k=1}^{K}\rho_{k}^{\left(k\right)}z_{k}^{\left(k\right)}{ℋ}_{k}^{H}{ℋ}_{k}$ and μ>0 is a constant selected to satisfy:


$$\begin{array}{c} \sum_{k=1}^{K}{\left\|{\left(\Psi^{\left(\kappa \right)}+\mu I_{M}\right)}^{-1}\rho_{k}^{\left(\kappa \right)}b_{k}^{\left(\kappa \right)}\right\|}^{2}=P. \end{array}$$

To compute μ, we apply the bisection method to solve the above nonlinear equation.

### 3.2 Programmable reflecting elements’ descent iteration

Similar to section 3.1, the goal is to determine the next iterative point, $\Theta^{\left(\kappa +1\right)}$, such that it satisfies the improvement condition: $\hat{G}({𝐐}^{\left(\kappa +1\right)},\Theta^{\left(\kappa +1\right)})>\hat{G}({𝐐}^{\left(\kappa +1\right)},\Theta^{\left(\kappa \right)})$. Since the diagonal matrix Θ contains unit-modulus elements, maximizing (13a) w.r.t. Θ is equivalent to maximizing w.r.t. the individual phase shift $\theta_{f}$. Following the approach in [24], the original objective function (14a) can be transformed into the following lower bound


$$\begin{array}{c} \hat{G}({𝐐}^{\left(\kappa +1\right)},\Theta )\geq {\tilde{x}}^{\left(\kappa \right)}+2ℜ\left\{\sum_{f=1}^{F}(\Delta_{f}^{\left(\kappa \right)})e^{j\theta_{f}}\right\}, \end{array}$$ (17)

where ${\tilde{x}}^{\left(\kappa \right)}$ and $\Delta_{f}^{\left(\kappa \right)}$ are constants that are independent of Θ, with $\Delta_{f}^{\left(\kappa \right)}$ can be calculated as $\Delta_{f}^{\left(\kappa \right)}≜{\tilde{y}}^{\left(\kappa \right)}(f)-\sum_{\hat{f}=1}^{F}\Gamma^{\left(\kappa \right)}(f,\hat{f})+\lambda_{\max}\left(\Phi^{\left(\kappa \right)}\right)e^{-j\theta_{f}^{\left(\kappa \right)}}$. We call ${\tilde{y}}^{\left(\kappa \right)}(f)$ is the “phase matrix decomposition” coefficient of (14a) at the point ${𝐪}_{j}^{\left(\kappa +1\right)}$, which is given as:


$$\begin{array}{cc} {\tilde{y}}^{\left(\kappa \right)}(f) & ≜\sum_{k=1}^{K}\rho_{k}^{\left(\kappa \right)}{\tilde{y}}_{k}^{\left(\kappa \right)}(f),\\ {\tilde{y}}_{k}^{\left(\kappa \right)}(f) & ≜\frac{\gamma_{k}^{\left(\kappa \right)}(f)}{\sum_{j\text{≠}k}{\left|{ℋ}_{k}^{\left(\kappa \right)}{𝐪}_{j}^{\left(\kappa +1\right)}\right|}^{2}+\sigma_{k}^{2}},\\ \gamma_{k}^{\left(\kappa \right)}(f) & ≜{\left({𝐪}_{k}^{\left(\kappa +1\right)}\right)}^{H}{ℋ}_{k}^{\left(\kappa \right)}{𝐡}_{k}\Upsilon_{f}𝐆{𝐪}_{k}^{\left(\kappa +1\right)}, \end{array}$$

where $\Upsilon_{f}$ is the F×F matrix with 1 on the main diagonal at entry (*f*,*f*), ${\tilde{y}}_{k}^{\left(\kappa \right)}(f)$ and $\gamma_{k}^{\left(\kappa \right)}(f)$ are coefficients at user *k* to compute ${\tilde{y}}^{\left(\kappa \right)}(f)$ accordingly. Note that we can represent Θ as $\Theta =\sum_{f=1}^{F}e^{j\theta_{f}}\Upsilon_{f}$, hence introducing the term “phase matrix decomposition.” Similarly, we call $\Phi^{\left(\kappa \right)}$ the “convex coefficient matrix,” as it results from coefficients of a convex approximation, which is calculated by:


$$\begin{array}{cc} \Phi^{\left(\kappa \right)} & ≜\sum_{k=1}^{K}\sum_{j=1}^{K}\gamma_{k}^{\left(\kappa \right)}{\tilde{z}}_{k}^{\left(\kappa \right)}{\left(\alpha_{k,j}^{\left(\kappa \right)}\right)}^{H}\alpha_{k,j}^{\left(\kappa \right)},\\ \alpha_{k,j}^{\left(\kappa \right)}(f) & ≜{𝐡}_{k}\Upsilon_{f}𝐆{𝐪}_{j}^{\left(\kappa +1\right)}, \end{array}$$

$$\begin{array}{cc} {\tilde{z}}_{k}^{\left(\kappa \right)} & ≜\frac{{\left|{ℋ}^{\left(\kappa \right)}{𝐪}_{k}^{\left(\kappa +1\right)}\right|}^{2}}{{\tilde{\mu }}_{k}^{\left(\kappa \right)}\left({\tilde{\mu }}_{k}^{\left(\kappa \right)}+{\left|{ℋ}_{k}^{\left(\kappa \right)}{𝐪}_{k}^{\left(\kappa +1\right)}\right|}^{2}\right)},\\ {\tilde{\mu }}_{k}^{\left(\kappa \right)} & ≜\sum_{j\neq k}{\left|{ℋ}_{j}^{\left(\kappa \right)}{𝐪}_{j}^{\left(\kappa +1\right)}\right|}^{2}+\sigma_{k}^{2}. \end{array}$$

Lastly, we introduce the “convex approximation matrix” $\Gamma^{\left(\kappa \right)}$, which is the convex lower bound of (14a) at the point $\left({𝐐}^{\left(\kappa +1\right)},\Theta^{\left(\kappa \right)}\right)$:


$$\begin{array}{c} \Gamma^{\left(\kappa \right)}(f,\hat{f})≜e^{-j\theta_{\hat{f}}^{\left(\kappa \right)}}\Phi^{\left(\kappa \right)}(f,\hat{f}). \end{array}$$

Noting that for any affine combination of functions of the form $ℜ\left\{ce^{j\theta_{n}}\right\}=|c|\cos\left(∠c+\theta_{n}\right)$, each function is maximized at $\theta_{n}=-∠c$ since cos(x)≤1. The closed-form solution of (17) is obtained by


$$\begin{array}{c} \theta_{f}^{\left(\kappa +1\right)}=-∠\Delta_{f}^{\left(\kappa \right)},f\in ℱ. \end{array}$$ (18)

In summary, by apply iteratively (16) and (18) until the objective function (13a) converges, we can find an sub-optimal point $\left({𝐐}_{\text{opt}},\Theta_{\text{opt}}\right)$ for problem (13). The summary of the iterative procedure is given in Alg. 1.

**Algorithm 1 Proposed Algorithm for problem (13)**

**Require:** ${𝐡}_{k}$, 𝐆, $\sigma_{k}$, $\Theta^{\left(0\right)}$, ${𝐐}^{\left(0\right)}$

1: **while** $\left|G^{\left(\kappa -1\right)}-G^{\left(\kappa \right)}\right|\geq \epsilon_{g}$ **do**

2:   Update ${𝐐}^{\left(\kappa \right)}$ based on (16)

3:   Update $\Theta^{\left(\kappa \right)}$ based on (18)

4:   κ←κ+1

5: **end while**

**Ensure:** Optimized variables ${𝐐}_{\text{opt}}$, $\Theta_{\text{opt}}$

### 3.3 Hybrid beamforming derivation by matrix factorization

In this section, we revisit (12) to derive its optimal solution from $\left({𝐐}_{\text{opt}},\Theta_{\text{opt}}\right)$. Notice that by relaxing the discrete constraints (12c) and (12d), $\Theta_{\text{opt}}$ is the optimal solution for (12). To find the optimal analog and digital beamforming matrices, $\left({𝐕}_{\text{opt}},{𝐖}_{\text{opt}}\right)$, from the optimal fully-digital beam ${𝐐}_{\text{opt}}$, a process known as splitting the fully-digital beam. We consider the following matrix factorization problem [33]


$$\begin{array}{cc} \min_{𝐖,𝐕}\; & \zeta ≜\|{𝐐}_{\text{opt}}-𝐕𝐖{\|}_{ℱ}^{2} \end{array}$$ (19a)

$$\begin{array}{c} \text{s.t.}\;\sum_{k=1}^{K}∥𝐕𝐖{∥}^{2}\leq P_{0}, \end{array}$$ (19b)

$$\begin{array}{cc} & \|v_{n,d}\|\leq 1,\;\forall n\in 𝒩,\forall d\in 𝒟. \end{array}$$ (19c)

Since the variables 𝐕 and 𝐖 are coupled in (19a), we again employ the BCD framework. This framework leverages an alternating procedure that, in each iteration η, solves for a sub-optimal solution to (19) by combining the Least Squares (LS) solution and Riemannian manifold optimization [34]. Specifically, ${𝐖}^{\left(\eta \right)}$ is determined based on ${𝐕}^{\left(\eta \right)}$ using the following normalized Least Square (LS) solution


$$\begin{array}{c} {𝐖}^{\left(\eta \right)}=\sqrt{P_{0}}\frac{({𝐕}^{\left(\eta \right)}{)}^{†}{𝐐}_{\text{opt}}}{{\left\|({𝐕}^{\left(\eta \right)}{)}^{†}{𝐐}_{\text{opt}}\right\|}_{ℱ}}, \end{array}$$ (20)

which satisfies the power constraint (19b). Consequently, the remaining task is to solve 𝐕 while keeping ${𝐖}^{\left(\eta \right)}$ fixed. Define


$$\begin{array}{cc} {𝐕}_{vec} & ≜[{\tilde{𝐯}}_{1}^{T},\;{\tilde{𝐯}}_{2}^{T},\;...,\;{\tilde{𝐯}}_{N}^{T}{]}^{T}\in {\mathbb{C}}^{M_{S}N\times 1}, \end{array}$$ (21a)

$$\begin{array}{cc} {𝐪}_{vec} & ≜[{𝐪}_{1}^{T},\;{𝐪}_{2}^{T},\;...,\;{𝐪}_{K}^{T}{]}^{T}\in {\mathbb{C}}^{KM\times 1}, \end{array}$$ (21b)

$$\begin{array}{cc} {𝐖}_{k,vec} & ≜[\begin{array}{cccc} {𝐰}_{k_{1},1}^{\left(\eta \right)} & & & \\ \vdots & {𝐰}_{k_{2},1}^{\left(\eta \right)} & & \\ {𝐰}_{k_{1},M_{S}}^{\left(\eta \right)} & \vdots & \ddots & {𝐰}_{k_{N},1}^{\left(\eta \right)}\\ & {𝐰}_{k_{2},M_{S}}^{\left(\eta \right)} & \cdots & \vdots \\ & & & {𝐰}_{k_{N},M_{S}}^{\left(\eta \right)} \end{array}], \end{array}$$ (21c)

$$\begin{array}{cc} {𝐖}_{vec}^{\left(\eta \right)} & ≜[{𝐖}_{1,vec}^{T},\;{𝐖}_{2,vec}^{T},\;...,\;{𝐖}_{K,vec}^{T}{]}^{T}\in {\mathbb{C}}^{KM\times M_{S}N}, \end{array}$$ (21d)

where ${\tilde{𝐯}}_{n}=[{\tilde{v}}_{n}(1),...,{\tilde{v}}_{n}(M_{S})]$ contains non-zero elements of column *n* of 𝐕 and ${𝐰}_{k_{n},1}^{\left(\eta \right)}$ is the row vector of a $M_{S}\times M_{S}$ matrix with zeros everywhere except digital beamforming element $w_{n,k}$ on its diagonal for all n∈𝒩 and k∈𝒦. We note that the shifted zeros of each column vector in ${𝐖}_{k,vec}$ are exactly of size $\Delta M_{S}$. Then, ${𝐯}_{\text{vec}}$ is the solution to the following problem:


$$\begin{array}{c} \underset{{𝐯}_{\text{vec}}\in V}{\mathrm{minimize}}\;\tilde{\zeta }={\left\|{𝐪}_{\text{vec}}-{𝐖}_{\text{vec}}^{\left(\eta \right)}{𝐯}_{\text{vec}}\right\|}^{2}, \end{array}$$ (22)

where $V≜\left\{𝐯\in {\mathbb{C}}^{M_{S}N\times 1}:\left|v_{m}\right|=1,\;m=1,...,M_{S}N\right\}$ is a manifold representing the feasible set of 𝐯. $v_{m}$ denotes the *m*-th element of 𝐯. Here, V is a complex circle manifold with the corresponding tangent space $T_{𝐯}V=\left\{𝐯\in {\mathbb{C}}^{M_{S}N\times 1}:ℜ\left(𝐯\circ {𝐯}^{*}\right)=0_{M_{S}N}\right\}$, where $0_{M_{S}N}$ represents the vector of $M_{S}N$ zeros. The problem (22) is known as a Riemannian manifold minimization problem which can be efficiently solved by the manifold optimization technique. Specifically, we adopt the Riemannian Conjugate Gradient (RCG) algorithm. In general, the procedure of the RCG algorithm is similar to the infamous Gradient Descent Algorithm [20,34], as we first need to calculate the gradient of the objective function:


$$\begin{array}{c} \nabla \zeta =-2({𝐖}_{\text{vec}}^{\left(\eta \right)}{)}^{H}({𝐪}_{\text{vec}}-{𝐖}_{\text{vec}}^{\left(\eta \right)}{𝐯}_{\text{vec}}). \end{array}$$ (23)

*Remark on discrete phase activation:* At each iteration of Algorithm 2, the continuous-phase solutions of both the RIS and the analog beamformer are immediately projected onto their corresponding discrete phase sets. Specifically, each RIS phase coefficient is mapped to the nearest element in ${𝒮}_{r}$, and each non-zero entry of the analog beamforming matrix is projected onto the discrete phase set ${𝒮}_{a}$. As a result, the discrete phase constraints are enforced per iteration, ensuring that all intermediate solutions remain hardware-feasible throughout the alternating optimization.

It is worth noting that the above discrete phase projection is applied after each continuous update obtained via Riemannian optimization. Each iteration of the RCG algorithm itself consists of four principal operations, namely, computing the Riemannian gradient, performing vector transport to map the previous search direction onto the tangent space at the new point, updating the conjugate search direction using the transported vector and current gradient, and projecting the updated point back to the manifold. With the key steps introduced above, the computation process for solving the problem (22) in each iteration *i* is expressed as follows:


$$\begin{array}{ccc} \mathrm{grad}\zeta_{i} & = & \nabla \zeta_{i}-ℜ\left(\nabla \zeta_{i}\circ {𝐯}_{\text{vec},i}^{*}\right)\circ {𝐯}_{\text{vec},i}, \end{array}$$ (24)

$$\begin{array}{c} {𝒯}_{i-1\to i}\left(\pi_{i-1}\right)=ℜ\left(\pi_{i-1}\circ {𝐯}_{\text{vec},i}^{*}\right)\circ {𝐯}_{\text{vec},i}, \end{array}$$ (25)

$$\begin{array}{c} \pi_{i}=-\mathrm{grad}\zeta_{i}+\mu_{i}\left(\pi_{i-1}-{𝒯}_{i-1\to i}\left(\pi_{i-1}\right)\right), \end{array}$$ (26)

$$\begin{array}{c} {𝐯}_{\text{vec},i+1}=\frac{{𝐯}_{\text{vec},i}+\delta_{i}\pi_{i}}{\left|{𝐯}_{\text{vec},i}+\delta_{i}\pi_{i}\right|}. \end{array}$$ (27)

**Fig 2:**
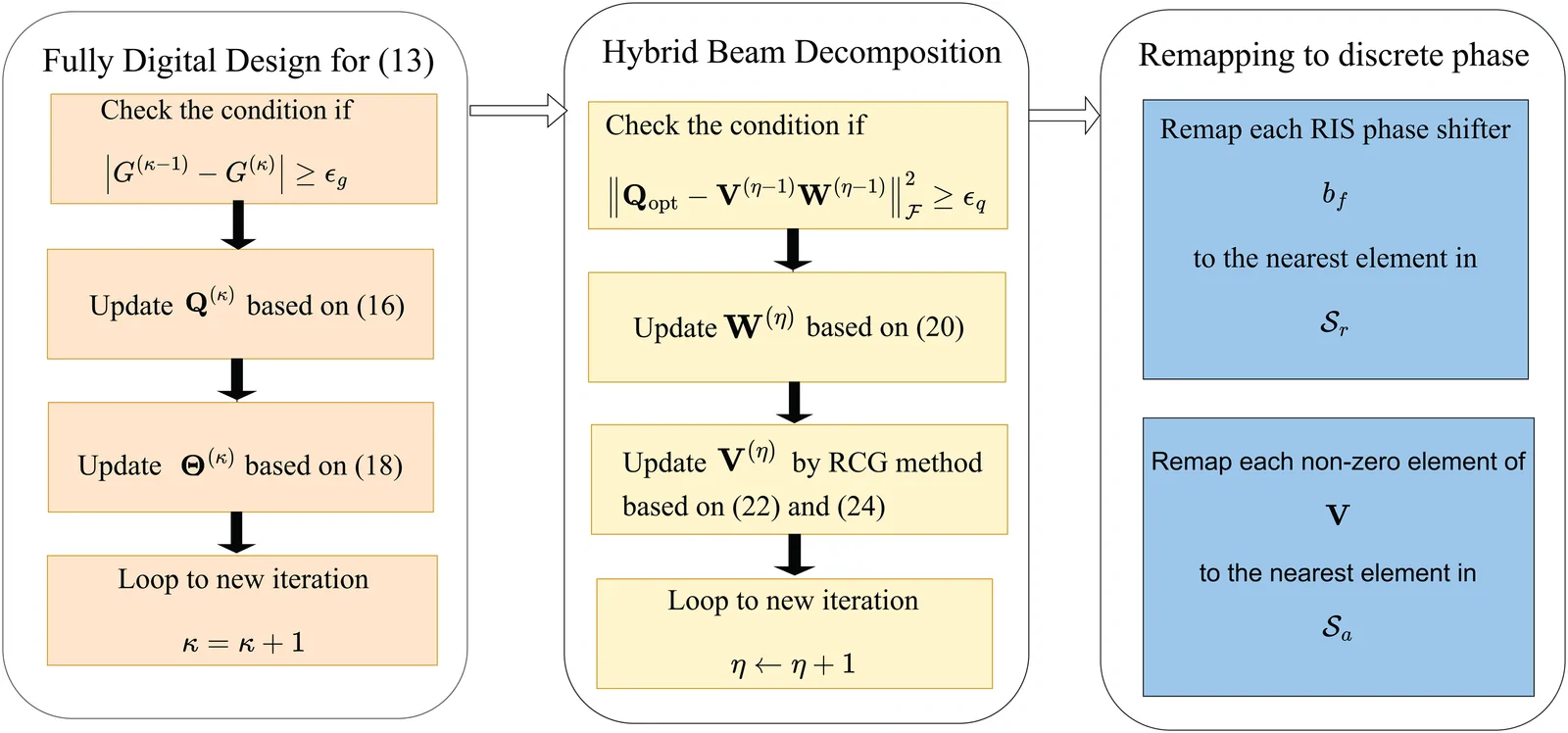
Flow diagram of the proposed algorithm for solving problem (12).

It is important to note that the proposed algorithm operates in an iterative manner, and without an appropriate initialization strategy, it may suffer from slow convergence and significant computational complexity. To overcome this, we initialize the analog beamforming ${𝐕}_{0}$ by


$$\begin{array}{c} {\left[{𝐕}_{0}\right]}_{i,j}=e^{∠[𝐔{]}_{i,j}}, \end{array}$$ (28)

where $𝐔\in {\mathbb{C}}^{M\times N}$ is taken from the first *N* eigenvectors of ${𝐐}_{\text{opt}}{𝐐}_{\text{opt}}^{H}$, and $∠[𝐔{]}_{i,j}$ represents the phase of the (*i*, *j*)-th entry of 𝐔. This initialization approach is based on the analog precoding scheme in [34]. Toward this end, the procedure for solving problem (12) is summarized in Alg. 2 and the summarizing flow-diagram is given in Fig 2. From the flow diagram, the overall optimization can be interpreted as an approximate decomposition of a highly coupled non-convex problem into a convex subproblem iteratively. In the fully digital design stage, the update for $\left({𝐐}^{\left(\kappa \right)},\Theta^{\left(\kappa \right)}\right)$ is based on Lagrangian duality and the optimal phase of an affine sum. In the hybrid beam decomposition stage, we try to minimize the Euclidean distance between the optimal FD beam and the actual HBF beam, leading to the LS solution for ${𝐖}^{\left(\eta \right)}$ and RCG solution for ${𝐕}^{\left(\eta \right)}$. Together, these steps progressively enforce unit-modulus constraints while preserving the performance-oriented objective, which results in a tractable optimal solution.

*Remark on the local optimum of proposed method:* We note that the solution obtained by the BCD technique always converges to a local optimum as the sequence created by iteratively optimizing the objective is a monotonic sequence. However, no guarantee of global optimality can be proved, as the original problem is strongly nonconvex and mixes integer variables. Hence, our proposed algorithm only guarantees obtaining a local optimum.

**Algorithm 2 Proposed Algorithm for problem (12)**

**Require:** ${𝐡}_{k}$, 𝐆, $\sigma_{k}$

1: Initialize $\Theta^{\left(0\right)}=I_{F\times F}$ and ${𝐐}^{\left(0\right)}$ by (29)

2: Acquire $\Theta_{\text{opt}}$ and ${𝐐}_{\text{opt}}$ based on Alg. 1

3: **while** ${\left\|{𝐐}_{\text{opt}}-{𝐕}^{\left(\eta -1\right)}{𝐖}^{\left(\eta -1\right)}\right\|}_{ℱ}^{2}\geq \epsilon_{q}$ **do**

4:   Update ${𝐖}^{\left(\eta \right)}$ by applying (20)

5:   Update ${𝐕}^{\left(\eta \right)}$ based on vectorization (22) and apply RCG algorithm (24) until convergence

6:   η←η+1

7: **end while**

8: Remap each RIS phase shifter $b_{f}$ to the nearest element in ${𝒮}_{r}$

9: Remap each non-zero element of 𝐕 to the nearest element in ${𝒮}_{a}$

**Ensure:** Optimized variables 𝐕 and 𝐖

*Complexity Analysis of Alg. 2: To analyze the computational complexity of the proposed joint hybrid beamforming and RIS phase shifts design, it is essential to compute the complexity of Alg. 1 along with the complexity of the RCG algorithm. We note that for any dense square matrix* $A\in {\mathbb{C}}^{n\times n}$*, the computational complexity to calculate its inverse is* $𝒪(n^{3})$ *[*35*]. We also observe that the matrix product between two matrices* $A\in {\mathbb{C}}^{n\times m}$ *and* $B\in {\mathbb{C}}^{m\times p}$ *is* 𝒪(mnp)*. Thus, the complexity of (16) is* $𝒪(M^{3}K)$ *for a fixed value of* μ*. However, to select the constant* μ*, we need an additional iteration complexity of* $𝒪(M^{3}K/\log(\epsilon_{\mu }))$*, where* $\epsilon_{\mu }$ *is the target accuracy of the bisection method, since this method is* $𝒪(\frac{1}{\log(\epsilon_{\mu })})$ *[*36*] and at each iteration we need to calculate the inverse and matrix multiplication of* $𝒪(M^{3}K)$*. Next, we consider the complexity of updating* $\Theta^{\left(\kappa \right)}$*. We recall the definition of* $\Delta_{f}^{\left(\kappa \right)}$ *as (18), which is* $𝒪(K^{2}F^{2}+FM)$*. The complexity of a two-block BCD algorithm has a complexity of* $𝒪(\frac{1}{\sqrt{\epsilon_{g}}})$ *[*37*]. Thus, the complexity of Alg. 1 is* $𝒪((\frac{M^{3}K}{\log(\epsilon_{\mu })}+K^{2}F^{2}+FM)\frac{1}{\sqrt{\epsilon_{g}}})$*. Similarly, we show the complexity to compute* ${𝐖}^{\left(\eta \right)}$ *to be* 𝒪(MNK) *while the RCG algorithm for update* ${𝐕}^{\left(\eta \right)}$ *is* $𝒪(\epsilon_{v}^{-2})$*, where* $\epsilon_{v}^{-2}$ *is the target accuracy of RCG. Since our beam decomposition leverages two-block BCD, its complexity is* $𝒪((MNK+\epsilon_{v}^{-2})\frac{1}{\sqrt{\epsilon_{q}}})$*. Hence, the total complexity of Alg. 2 is* $𝒪((\frac{M^{3}K}{\log(\epsilon_{\mu })}+K^{2}F^{2}+FM)\frac{1}{\sqrt{\epsilon_{g}}}+(MNK+\epsilon_{v}^{-2})\frac{1}{\sqrt{\epsilon_{q}}})$.

### 3.4 Effective initialization for GM rate optimization problem

Given (16), we assume that the parameter μ can be found by applying the bisection method, which requires prior knowledge of the interval of opposite sign, i.e., the interval $\left[0;\mu_{0}\right]$ that the function $\sum_{k=1}^{K}{\left\|{\left(\Psi^{\left(\kappa \right)}+\mu I_{M}\right)}^{-1}\rho_{k}^{\left(\kappa \right)}b_{k}^{\left(\kappa \right)}\right\|}^{2}-P_{0}$ exhibits a sign change. As such, we wish to find an appropriate initial value $\left({𝐐}^{\left(0\right)},\Theta^{\left(0\right)}\right)$ so that we could ensure $\mu_{0}$ is within acceptable limits. We first initialize $\Theta^{\left(0\right)}=I_{F\times F}$, which corresponds to configuring the RIS as a passive reflector, changing only the direction of the incoming signals without conducting any phase shift. Consequently, the initialization of ${𝐐}^{\left(0\right)}$ is given in (29) such that


$$\begin{array}{c} {𝐐}_{0}=\xi_{P_{0}}\sqrt{P_{0}}(\frac{({𝐇}^{H}\Theta 𝐆{)}^{†}}{{\left\|{𝐇}^{H}\Theta 𝐆{)}^{†}\right\|}_{ℱ}}. \end{array}$$ (29)

Here, we denote $𝐇≜[{𝐡}_{1},{𝐡}_{2},...,{𝐡}_{K}]$. ${𝐐}_{per}$ is a K×M matrix with each element independently drawn from the circularly symmetric complex Gaussian distribution, i.e., $\left[{𝐐}_{per}{]}_{\left(i,j\right)}\sim CN(0,1\right)$, and $\xi_{P_{0}}<1$ is a parameter to guarantee constraint (13b). The primary motivation for this formulation is to achieve uniform power allocation across users while simultaneously suppressing multi-user interference. In particular, considering the linear independence of the user channels ${𝐡}_{k}$, the matrix operation simplifies such that ${𝐇}^{H}\Theta 𝐆({𝐇}^{H}\Theta 𝐆{)}^{†}={𝐈}_{K\times K}$. This condition effectively nullifies all inter-user interference (IUI), achieving perfect isolation between the *K* users.

## 4 Performance evaluation

**Fig 3:**
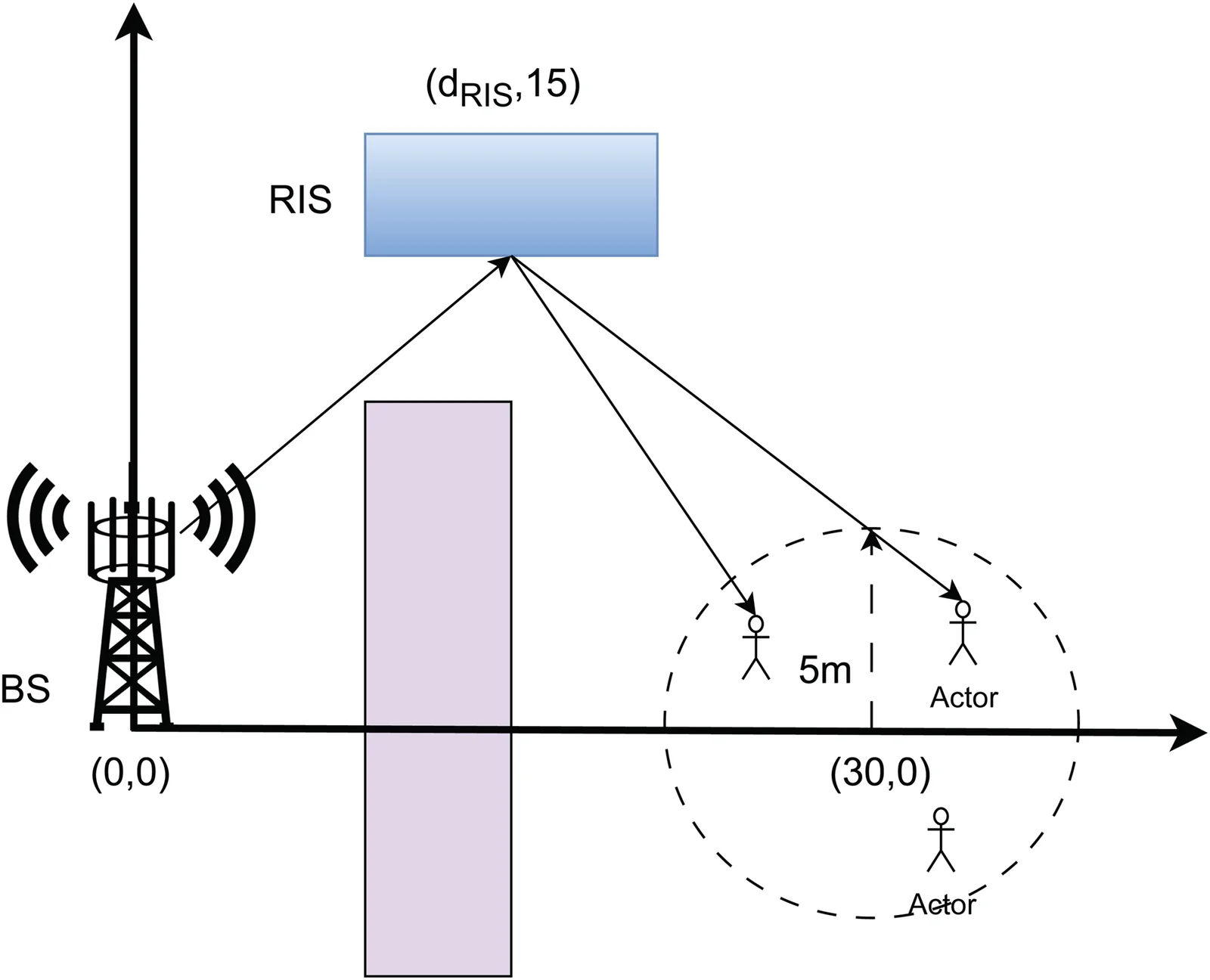
Simulated RIS-assisted hybrid beamforming (HBF) scenario.

In this section, we present the performance evaluation of our proposed algorithms through system-level simulations. We model an RIS-aided multiuser millimeter-wave (mmWave) communication system operating at 28 GHz [20]. The BS is located at (0,0) in the two-dimensional (2D) Cartesian coordinate system and is equipped with a 10×10 UPA structure, totaling *M* = 100 antennas and *N* = 10 RF chains. The thermal noise power is set to $\sigma_{k}^{2}=-174$ dBm. The overall system layout is illustrated in Fig 3. The system incorporates an RIS, positioned at (*d*_RIS_, 15). This surface is equipped with a uniform 10×10 rectangular array, totaling 100 reflective unit cells. The *K* users are modeled as being uniformly and randomly distributed in a circular area with a 5-meter radius, centered at (50,0). For the mmWave channel model, we adopt a clustered channel approach with $N_{cl1}=N_{cl2}=5$ clusters and $N_{\text{ray}1}=N_{\text{ray}2}=10$ rays per cluster [20]. The azimuth and elevation angles of arrival and departure are modeled using the Laplacian distribution with an angle spread of 10 degrees. The complex gains $\alpha_{il}$ and $\beta_{il}$ follow a complex Gaussian distribution $CN(0,10^{-0.1PL(d)})$, where the path loss factor *PL*(*d*) is defined by [20]


$$\begin{array}{c} PL(d)=\varphi_{a}+10\varphi_{b}\log_{10}(d)+\varphi_{c}\;(\text{dB}), \end{array}$$ (30)

where $\varphi_{a}=72.0$, $\varphi_{b}=2.92$, $\varphi_{c}\sim 𝒩(0,\sigma^{2})$, and σ=8.7 dB. Unless specified otherwise, the remaining system parameters are set as follows: *K* = 3, $d_{RIS}=15$, $\epsilon_{g}=\epsilon_{q}=10^{-3}$, $\gamma_{k}=10$ dB, ∀k∈𝒦. We perform simulations based on averaging over 500 independent channel realizations.

**Fig 4:**
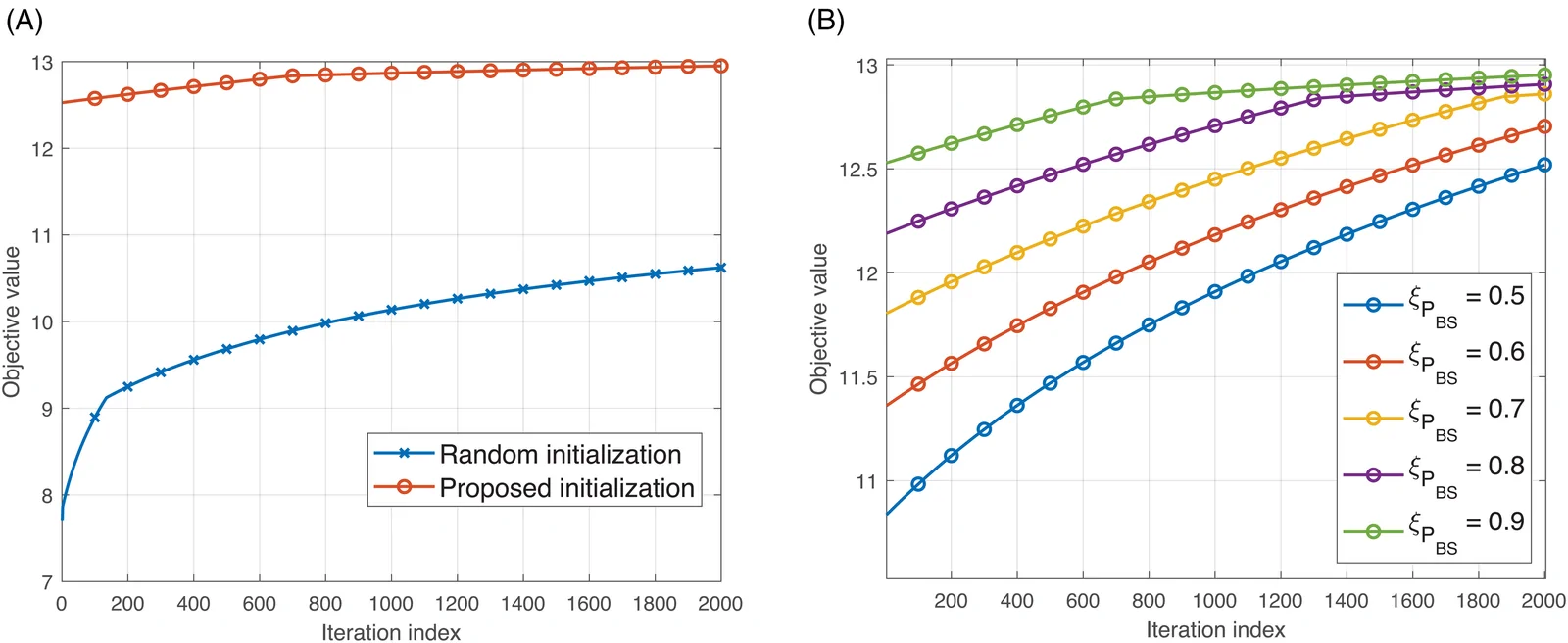
Convergence behavior of Algorithm 1. (A) Random initialization versus proposed initialization. (B) Effect of the parameter $\xi_{P_{0}}$.

Fig 4 illustrates the convergence behavior of the proposed Alg. 1. Specifically, Fig 4(A) compares the convergence rate achieved using our proposed initialization against a random scheme. For the random baseline, we initialized the matrix ${𝐐}_{ran}$ using complex normal distribution CN(0,1) for each element, then normalized it to set the starting point as ${𝐐}_{0}=\xi_{P_{0}}\sqrt{P_{0}}\frac{{𝐐}_{ran}}{{\left\|{𝐐}_{ran}\right\|}_{ℱ}}$. As shown in Fig 4(A), the random scheme starts with a very poor geometric mean value and takes more than 2000 iterations to converge to a stationary point. At the same time, the proposed initialization outperforms the initial value from the beginning of the algorithm, while achieving a faster convergence rate within the first 800 iterations and improving the objective value by 18.18%. This significant difference arises because the limit point of the random scheme is merely a random stationary point with no guarantee of global optimality, whereas our proposed approach is designed to effectively cancel interference degradation, thus achieving a superior solution. Fig 4(B) illustrates the convergence speed of the different power usage parameters $\xi_{P_{0}}$. The convergence speed is directly proportional to $\xi_{P_{0}}$, which is expected since the power resource is utilized from the initial step of the iterative procedure. Critically, we observe that the iterative procedure converges to the same limit point irrespective of the initial value of $\xi_{P_{0}}$.

**Fig 5:**
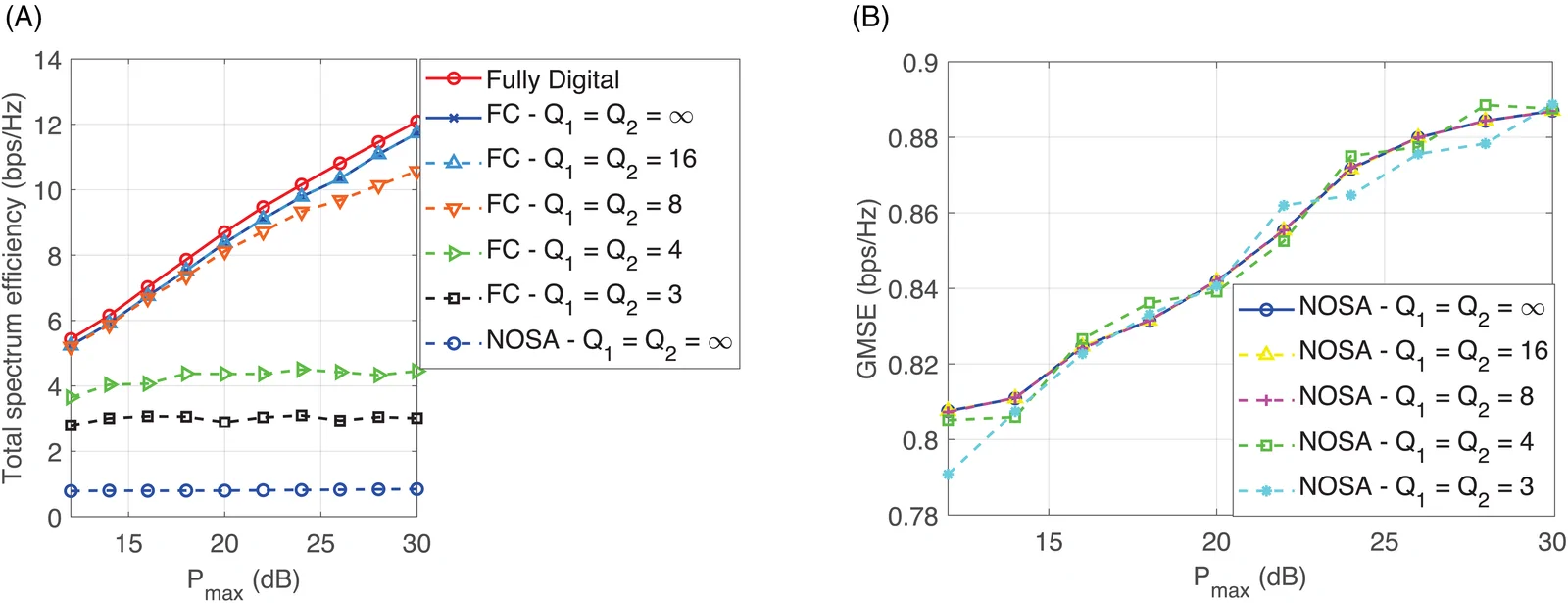
GMSE versus transmit power $P_{\max}$. (A) Fully connected (FC) hybrid beamforming. (B) Non-overlapped subarray (NOSA) hybrid beamforming.

Fig 5 illustrates the overall performance across different beamforming configurations, where we assume $Q_{1}=Q_{2}=Q$. Specifically, Fig 5(A) shows that the FD scheme achieves the highest performance gains. This result is expected because the FD configuration dedicates one RF chain to each BS antenna, thereby fully exploiting the spatial degrees of freedom and maximizing the system capacity. Furthermore, the FC hybrid architecture consistently outperforms the NOSA approach even when the number of quantization bits is limited. This advantage arises from the higher density of non-zero elements in the FC connection matrix, which provides greater flexibility for the hybrid beamforming decomposition to approximate the optimal FD beamformer. The results indicate that approximately 8 bits of phase quantization are sufficient to achieve a favorable trade-off between hardware complexity and performance for the FC architecture.

Fig 5(B) presents the corresponding results for the NOSA architecture. In this case, the performance is noticeably lower due to the reduced spatial flexibility caused by the subarray structure. When the number of RF chains N≪M, each RF chain simultaneously controls the phases of multiple antennas, which restricts the beamforming resolution and limits the achievable beam decomposition gain. Consequently, the NOSA architecture experiences a larger performance gap compared with the FD benchmark. Nevertheless, as the number of quantization bits increases, the system performance gradually saturates and approaches the continuous phase-shift case $Q_{1}=Q_{2}=\infty$. These results suggest that using approximately 3 bits of phase quantization at both the BS and RIS provides a practical trade-off between hardware implementation complexity and achievable performance for the NOSA architecture.

**Fig 6:**
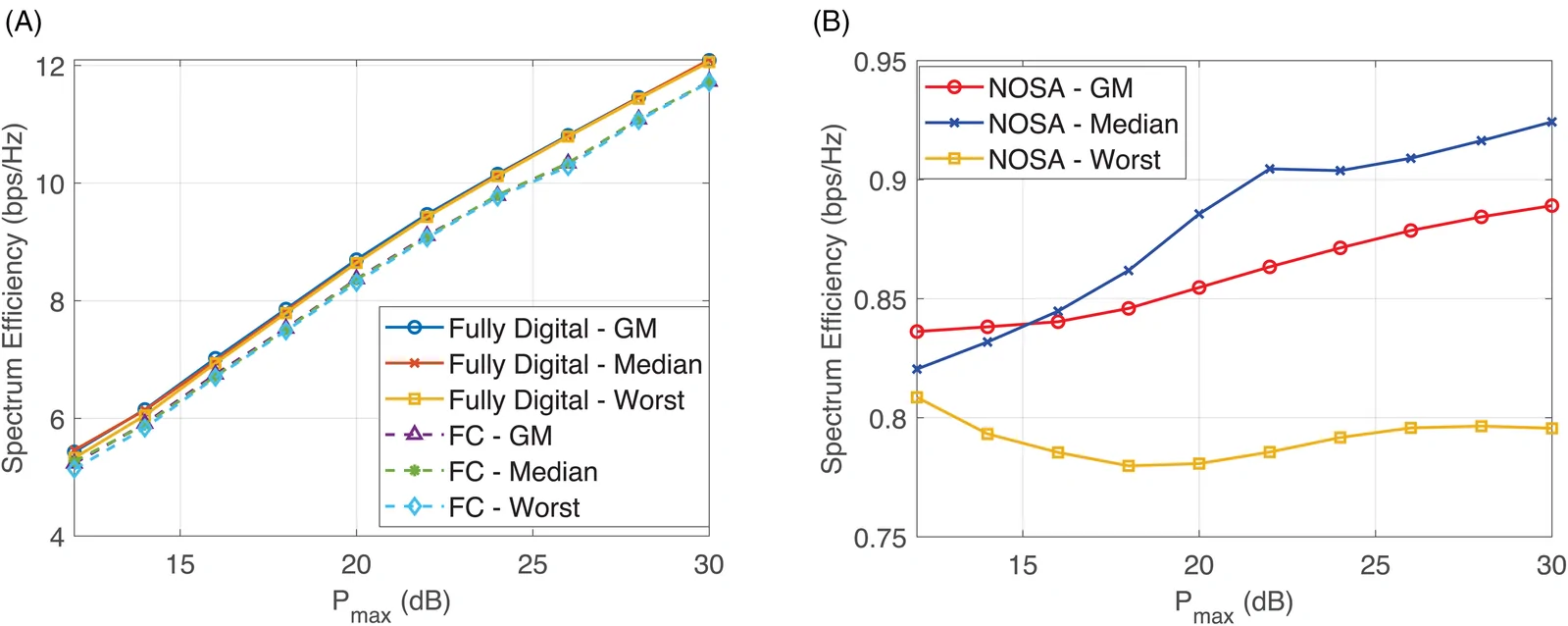
Rate distribution comparison between geometric mean (GM) and max–min designs. (A) Fully connected (FC) architecture. (B) Non-overlapped subarray (NOSA) architecture.

Fig 6 illustrates the relationship between the surrogate objective and user fairness. Specifically, we evaluate the worst-user rate and the median-user rate under the same simulation settings to verify that the GM design effectively improves the performance of the weakest user relative to FC and NOSA. As shown in Fig 6(A), the performance curve of each target user aligns with the geometric mean curve for both FD and FC architectures, confirming the fairness traits of the GM design. However, Fig 6(B) shows a considerable separation between the three benchmarks. This phenomenon arises from the trade-off between architectural complexity and QoS performance in the NOSA scheme, which provides a limited degree of freedom for analog beamforming to suppress inter-user interference. Nevertheless, the GM objective significantly improves the worst-user rate compared to median and dominant users for the NOSA case in the low SNR region while striking a balance between overall performance and maintaining an acceptable performance for the worst-user rate in the high SNR region, demonstrating the ability to reduce rate disparity across users. In detail, the worst-user rate maintains a stable performance around 0.8 bps/Hz for increasing levels of *P*_max_, while the median-user rate and GM rate increase from 0.85 to 0.9 bps/Hz. Nevertheless, the GM objective significantly improves the worst-user rate compared to the median and dominant users for the NOSA case in the low SNR region, while striking a balance between overall performance and maintaining an acceptable worst-user rate in the high SNR region, thereby demonstrating its ability to reduce rate disparity across users. Concretely, the worst-user rate remains stable at approximately 0.8 bps/Hz as *P*_max_ increases, whereas the median-user rate and the GM rate increase from 0.85 to 0.9 bps/Hz. Thus, even in a low spatial degree of freedom structure such as NOSA, maximizing the GM rate prevents user-rate collapse due to unfair resource distribution. Consequently, we note that a restricted spatial OSA configuration intensifies the trade-off between overall rate and user fairness, leading to a misalignment between the GM rate and the individual user rates compared to a more flexible configuration.

**Fig 7:**
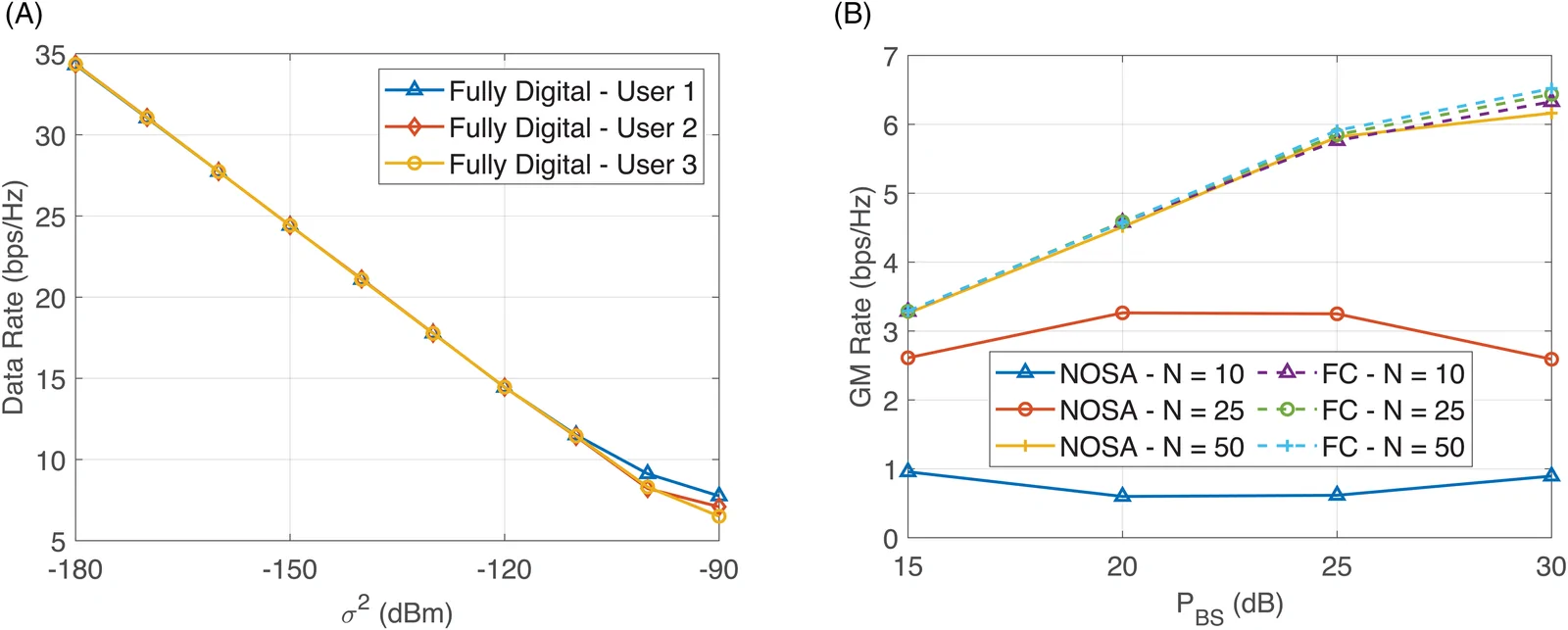
Performance evaluation under different system parameters. (A) Spectral efficiency of individual users versus noise power. (B) Spectral efficiency under different numbers of RF chains.

Fig 7(A) shows the inverse relationship between noise power and spectrum efficiency. As the noise power $\sigma_{k}^{2}$ increases, the spectrum efficiency for all users declines exponentially. Specifically, efficiency drops sharply from approximately 35 bps/Hz at a very low noise level, i.e., $\sigma_{k}^{2}=-180$ dBm to just under 5 bps/Hz at a high noise level, i.e., $\sigma_{k}^{2}=-90$ dBm. This decline confirms that, in the optimized beamforming scheme, Gaussian noise is the dominant factor limiting the achievable SINR and thus the spectrum efficiency. Considering the absence of LoS, the channel condition can significantly attenuate the signal power by a factor of −13 or −14 dB. This loss necessitates that the noise power $\sigma_{k}^{2}$ be kept below a threshold, typically −120 dBm, to ensure every user maintains a target achievable data rate.

The impact of the number of RF chains on the spectral efficiency of the hybrid beamforming system is illustrated in Fig 7(B). To isolate this effect, the noise power is fixed at $\sigma_{k}^{2}=-90$ dBm. As the number of RF chains increases, the spectral efficiency of the NOSA configuration improves monotonically due to the additional degrees of freedom available in the digital precoding stage. In the extreme case where *N* = *M* = 100, the NOSA architecture reduces to the FD configuration with $𝐕={𝐈}_{M\times N}$. Moreover, the results indicate that when the number of RF chains becomes sufficiently large, the NOSA architecture approaches the performance of the FC architecture. Our simulations show that this convergence typically occurs around *N* = *M*/2 = 50.

**Fig 8:**
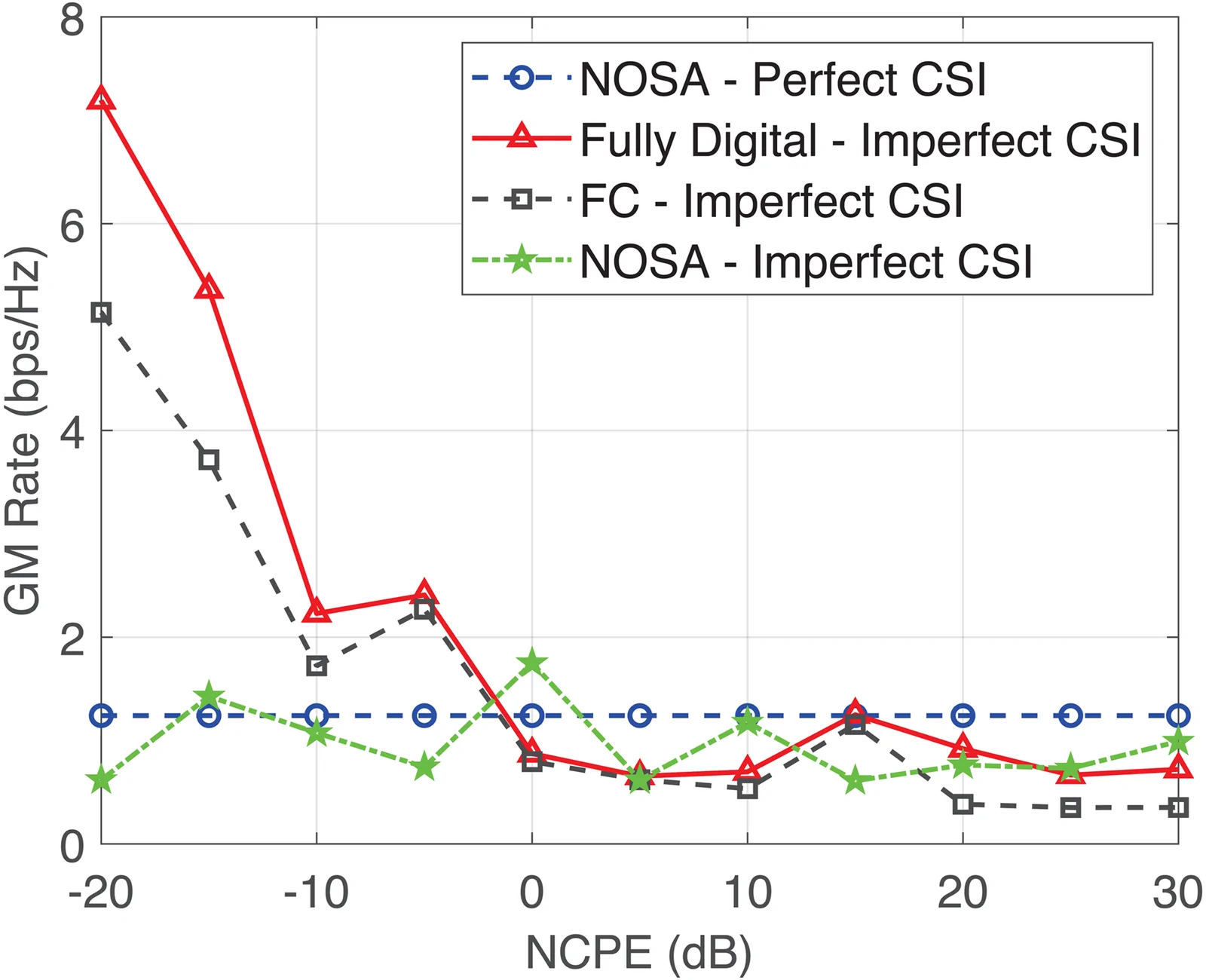
Geometric mean (GM) rate performance under imperfect channel estimation conditions.

Fig 8 presents a robustness comparison of different beamforming schemes under imperfect channel state information (CSI), where the system parameters are set to *N* = 50 and $Q_{1}=Q_{2}=\infty$. The considered hybrid beamforming configurations are consistent with those evaluated in Fig 5. Imperfect CSI may arise from several practical factors, including channel estimation errors, quantization during CSI feedback, and outdated CSI caused by transmission delay. In this work, the CSI imperfection is modeled as an additive perturbation noise. Specifically, the estimated channel of user *k* is expressed as


$$\begin{array}{c} {\hat{ℋ}}_{k}={ℋ}_{k}+{𝐍}_{k}, \end{array}$$

where ${ℋ}_{k}$ denotes the actual channel matrix and ${𝐍}_{k}$ represents the channel perturbation. The perturbation matrix is assumed to follow a complex Gaussian distribution ${𝐍}_{k}\sim CN(0,e_{k}^{2})$. Accordingly, the normalized channel perturbation error (NCPE) is defined as [29]


$$\begin{array}{c} {NCPE}_{k}=\frac{{\left\|{\hat{ℋ}}_{k}-{ℋ}_{k}\right\|}_{F}^{2}}{{\left\|{ℋ}_{k}\right\|}_{F}^{2}},\;k\in 𝒦. \end{array}$$ (31)

For simplicity, we assume identical perturbation levels for all users, i.e., $e_{1}=e_{2}=\cdots =e_{K}$. As illustrated in Fig 8, the performance of all beamforming schemes degrades as the channel estimation error increases. In particular, the GM-rate performance of the FD and FC configurations decreases rapidly with increasing NCPE and eventually falls below that of the NOSA architecture. When the NCPE level reaches 0 dB, meaning that the perturbation power is comparable to the channel power, the achievable GM rate saturates at approximately 2 bps/Hz. These results highlight the strong sensitivity of hybrid beamforming systems to CSI mismatch and indicate that accurate CSI estimation is essential for maintaining the desired quality-of-service (QoS).

## 5 Conclusions

In this paper, we investigated the spectral efficiency of an RIS-aided scalable hybrid beamforming system operating over the mmWave channel. Given the adopted OSA hybrid architecture at BS, we proposed a joint optimization algorithm to maximize the GM of users’ achievable rates. Based on existing works, we divide the hybrid beamforming problem into the FD beamforming sub-problem and the matrix decomposition problem. We then adopt the iterative BCD method and RCG to solve the resulting optimization problems. An effective initialization method is proposed to enhance the convergence rate of the algorithm. Numerical results demonstrate the effectiveness of the proposed framework in improving fairness-oriented spectral efficiency, convergence behavior, and architectural trade-offs under practical hardware constraints. Specifically, we observed an 18.18% objective value improvement compared to existing methods and identified suitable hardware phase resolutions for RIS-assisted hybrid beamforming systems.

The proposed framework is specifically designed to optimize fairness-oriented spectral efficiency under hardware constraints. Accordingly, the conclusions of this work are restricted to the evaluated aspects, including GM-rate performance, convergence behavior, fairness characteristics, architectural trade-offs, and sensitivity to CSI uncertainty under the considered system model. While the impact of CSI uncertainty has been partially examined through CSI perturbation analysis, energy-efficiency optimization, latency-aware design, and robust formulations for highly dynamic environments remain topics for further investigation.
