# Supplementary material for: Scalable RIS-aided hybrid beamforming for mmWave systems enables multiple sub-array architectures: A Geometric Mean Approach
Source: PLoS One. 2026 Aug 21;21(8):e0354965. doi: 10.1371/journal.pone.0354965 (PMC13496462; doi:10.1371/journal.pone.0354965)
Supplement: Supplementary file 1 [file pone.0354965.s001.pdf]

Comments to PONE-D-25-56680

This paper investigated a RIS-aided hybrid beamforming system designed for scalable operation in the millimeter wave channel. Generally, the authors have done an interesting work, however, some parts need to be further improved. The reviewer has the following concerns:

1. This paper mentions using geometric mean to enhance user fairness but fails to deeply analyze specific failure cases of existing sum-rate maximization methods in dynamic environments, such as not detailing how geometric mean handles channel fluctuations or user mobility.

2. The adopted the BCD algorithm and manifold optimization are essentially an extension of traditional optimization frameworks, and it does not explain why this framework has inherent advantages over traditional methods in convergence speed or resource efficiency.

3. The method design relies on ideal channel assumptions, such as perfect CSI or static interference, but does not account for practical estimation errors, feedback delays, or hardware nonlinearities.

4. It is suggested to introduce the following highly-impact recent works in RIS [R1]-[R4] field to highlight the state-of-art of this paper:

[R1] "Exploiting multi-layer refracting RIS-assisted receiver for HAP-SWIPT networks," *IEEE Transactions on Wireless Communications*, vol. 23, no. 10, pp. 12638-12657, Oct. 2024.

[R2] "Self-powered absorptive reconfigurable intelligent surfaces for securing satellite-terrestrial integrated networks," *China Communications*, vol. 21, no. 9, pp. 276-291, Sep. 2024.

[R3] "Refracting RIS aided hybrid satellite-terrestrial relay networks: Joint beamforming design and optimization," *IEEE Transactions on Aerospace and Electronic Systems*, vol. 58, no. 4, pp. 3717-3724, Aug. 2022.

[R4] "AI-driven seamless and massive access in space-air-ground integrated networks," *IEEE Wireless Communications*, vol. 32, no. 3, pp. 72-79, Jun. 2025.

5. The scheme does not discuss computational complexity or real-time processing requirements, which may cause high latency on resource-constrained base stations.

6. The performance evaluation only focuses on geometric mean rate and convergence, neglecting key metrics like energy efficiency, complexity, or anti-interference resilience.

7. The writing is somewhat abrupt in algorithm flow and symbol definitions, such as not clearly defining all abbreviations or optimization variables.
