# Supplementary material for: Scalable RIS-aided hybrid beamforming for mmWave systems enables multiple sub-array architectures: A Geometric Mean Approach
Source: PLoS One. 2026 Aug 21;21(8):e0354965. doi: 10.1371/journal.pone.0354965 (PMC13496462; doi:10.1371/journal.pone.0354965)
Supplement: Supplementary file 2 [file pone.0354965.s003.pdf]

Response to the Reviewers

Original Manuscript ID: PONE-D-25-56680
Original Article Title: Scalable RIS-aided Hybrid Beamforming for mmWave Systems enables Multiple Sub-array Architectures: A Geometric Mean Approach
Authors: Hoang M. Tran, Tung V. Dinh, Hoa T. Nguyen, Huy T. Nguyen

Dear Editor and Reviewers,

We appreciate the time you have dedicated to handling our manuscript and for giving us the opportunity to revise and resubmit it. In this revision, we have made the following updates:

- We have revised and clarified the system setup, along with the application of the considered system.
- We have updated and improved the description of the signal detection process at the target to make it more readable to readers.
- We have improved the discussion about the non-convexity and challenges in solving the problem of maximizing the mutual information.
- We have added a discussion on the convergence characteristic and clarified the accuracy of the result achieved by the proposed algorithm in solving the problem of maximizing the mutual information.
- We have rechecked and proofread the whole manuscript.

In the following, we provide point-to-point responses to the comments and actions that we have taken to address those issues. The raised comments and our answers are described in *italic* font and roman font, respectively. Unless stated otherwise, all the numbered items (figures, equations, etc.) in this response letter refer to those in the revised manuscript.

Furthermore, in the revised manuscript, the major changed parts, such as updated related works, more detailed explanations of the proposed system model, new numerical results, and more insightful discussions have been highlighted in blue color. Other minor corrections and rearrangements have not been highlighted. Please also note that in this response, we provide many relevant references to strongly support the correctness of some of our arguments. However, highly correlated references are cited in the revised manuscript.

Further, we would like the Editor to assign our article to the same set of Reviewers for further review. We do hope that the Editor and Reviewers find our explanations/justifications and revisions/improvements satisfactory.

Yours sincerely,

Executive Editor

General comments: *Thank you for submitting your manuscript on RIS-aided mmWave hybrid beamforming with overlapped subarray architecture. The paper addresses a timely topic and proposes a technically sound optimization framework combining geometric mean rate maximization, block coordinate descent, and Riemannian manifold-based hybrid decomposition. The Reviewers acknowledge the mathematical correctness of the approach and the promising numerical gains over existing methods.*

However, Reviewers have raised several substantial concerns regarding motivation, novelty clarification, contextualization with recent literature, fairness interpretation, algorithmic clarity, and experimental validation. These issues must be addressed thoroughly before the manuscript can be reconsidered for publication. A major revision is therefore required. Below, we summarize the required revisions by theme. You are expected to respond point-by-point to all Reviewer comments in a detailed rebuttal and revise the manuscript accordingly.

Authors' Response: We would like to thank the Editor for giving us an opportunity to revise and resubmit our manuscript. We have carefully addressed all the concerns raised by the Reviewers, revised and improved the paper accordingly. Details of these are specified in each Reviewer's response.

Consider 1 – *Motivation and Fairness Justification of the Geometric Mean Objective: A central concern raised by both Reviewers is that the choice of geometric mean (GM) rate maximization is insufficiently motivated. You must clearly explain what specific fairness or reliability problem GM rate addresses in RIS-aided hybrid beamforming systems that conventional sum-rate or proportional-fair objectives do not.*

Authors' Response: We appreciate the Editor for this useful comment. We have addressed all these concerns by each Reviewer's comment and included appropriate information in the revised manuscript accordingly.

(i) **Motivation and Fairness Justification of the Geometric Mean Objective**: The novelty behind choosing the geometric mean (GM) of data rates as the objective function lies in the trade-off of rate fairness among users with the overall network performance. Specifically, the overall network performance is often formulated as the weighted sum rate (SR) optimization problem in the literature [1][2]. However, this approach has the deficiency of allocating a large fraction of the sum-rate to a few users having good channel conditions, while leaving the rest of the users with almost zero rates. This motivates the formulation of fairness based on GM rate due to several key aspects:

- The geometric mean over $\prod_{k=1}^{K}(1+\mathrm{SINR}_k)$ is equivalent to the unweighted sum rate formulation, i.e. $\sum_{k=1}^{K} R_k$, since $\sum_{k=1}^{K}\log_2(1+\mathrm{SINR}_k)=\log_2\left(\prod_{k=1}^{K}(1+\mathrm{SINR}_k)\right)$. As such, the objective function $\max(\sum_{k=1}^{K} R_k) = \max\log_2\left(\prod_{k=1}^{K}(1+\mathrm{SINR}_k)\right)$ is equivalent to $\max\left(\prod_{k=1}^{K}(1+\mathrm{SINR}_k)\right)$.

- To achieve the fairness target, one can employ the max-min rate objective or proportional rate constraint for maximizing the rate of the users having poor channel conditions. However, the max-min rate approach leads to an undesirable degradation in the overall sum rate (SR) of the system while the proportional rate constraint introduces additional nonconvexity and computation complexity to the hybrid beamforming scheme. Furthermore, the authors in [3] pointed out that with suitable choice of parameters, the standard proportional fairness can be regarded as a max-min optimization problem. As an alternative solution to balance fairness and high sum rate while introduce little computation difficulty, the geometric mean (GM) rate maximization is proposed as a promising solution in the literature [4][5][6].

(ii) **Failure cases of existing sum-rate maximization methods**: In this paper, we consider the problem of jointly design hybrid beamforming and RIS phase shift under static channel condition. In particular, we consider the channel matrices $\mathbf{G}$ and $\mathbf{h}_k$ to be unchanged during the HBF design process. Thus, the consideration of geometric mean for channel fluctuations or user mobility falls outside the scope of this paper.

However, even with static channel condition, several papers in literature have addressed the imbalance of sum-rate maximization in ensuring QoS for users with poor channel condition. Specifically, the authors in [6] have shown the existence of zero-rate users, i.e. users with data rate equals to zero, when jointly design hybrid beamforming and RIS phase based on sum-rate maximization methods for the case of $K > M$. Furthermore, this paper demonstrates that the GM rate formulation ensures that no user experiences a zero rate under the same settings while maintains a considerable overall QoS. Hence, it can be seen that for the case of $K > M$, GM formulation can guarantee sufficient resource for user with inferior channel condition, while enhancing the overall rate of the network.

(iii) **Why GM-rate provides a necessary improvement.** Finally, the revised Introduction clarifies why GM-rate is not merely an alternative fairness metric, but a *necessary design choice* under the above conditions. Unlike sum-rate objectives, GM-rate explicitly penalizes vanishing user rates and enforces balanced performance within each transmission interval, thereby preventing service outage for blocked or cell-edge users.

This role of GM-rate is now explicitly articulated as follows:

> Geometric mean (GM) rate maximization provides a principled mechanism to address this fairness and reliability gap by explicitly balancing individual user rates [28]. Unlike sum-rate objectives, which are insensitive to individual rate collapse, GM-rate penalizes vanishing user rates and enforces balanced performance across all users within a single transmission interval. This property is particularly important in quasi-static RIS-assisted mmWave systems, where limited scheduling diversity and reliance on reflected paths prevent fairness from being achieved through long-term averaging. Under OSA-based hybrid beamforming, GM-rate naturally aligns with reduced spatial degrees of freedom by prioritizing robustness and service continuity over peak throughput.

These revisions clarify that GM-rate optimization is introduced to address a concrete reliability and fairness gap in RIS-assisted mmWave hybrid beamforming, particularly in blockage-prone or cell-edge scenarios where sum-rate-based designs fail to guarantee service continuity.

We have added the following discussion in the revised manuscript:

> Existing works in the literature often use the sum-rate method and the max-min method to quantify the quality of service (QoS) of the network, each of the formulation in favor the overall rates or resource fairness across users, respectively. To balance this trade-off between total QoS and resource fairness, a proportional fairness constraint is introduced as an alternative method, at the cost of additional complexity of non-convex constraints [33]. Thus, the recent GM rate maximization method overcomes this challenge by considering a different objective function [28].

Consider 2 – *Novelty Clarification and Contributions: While the contribution list is extensive, it lacks clarity regarding what is fundamentally new versus incremental. Please rewrite the contribution list so that each item reflects a distinct technical innovation, rather than a procedural step.*

Authors' Response: We appreciate the Editor for this useful comment. We have addressed all these concerns by each Reviewer's comment and included appropriate information in the revised manuscript accordingly.

In the revised manuscript, we streamlined the contribution list and rewrote each item to state a distinct technical advancement (rather than a sequence of steps). The revised contribution list is *highlighted in blue* in the Introduction and now reads:

> 1. We formulate a fairness-driven GM-rate maximization problem for RIS-assisted mmWave systems with OSA-based hybrid beamforming and discrete RIS phases, thereby explicitly addressing the reliability gap inherent in sum-rate-centric designs.
> 2. We propose an OSA-consistent Riemannian manifold factorization that maps the fully-digital GM-optimal precoder benchmark into a realizable hybrid analog–digital structure without resorting to matrix lifting or semidefinite relaxation.
> 3. We develop an interference-aware warm-start strategy that stabilizes the power-constrained precoder update and accelerates convergence by up to 18.18% compared with random initialization.

We also revised the **Novelty Statement** to explicitly separate the journal contributions from the prior conference paper. The following text has been added and *highlighted in blue*:

> This journal article substantially extends the authors' preliminary [30] through several conceptual and methodological advancements rather than incremental refinements. Specifically, the journal version introduces: (i) a refined fairness-oriented optimization formulation that explicitly targets user-rate imbalance instead of aggregate throughput maximization, which was not considered in the conference version; (ii) a novel OSA-consistent Riemannian manifold factorization mechanism that rigorously maps the fully-digital optimum into a realizable hybrid analog–digital structure with discrete phase constraints; and (iii) an explicit theoretical and practical positioning with respect to emerging AI- and DRL-based RIS optimization approaches.

Consider 3 – *Related Work and Missing Literature (Including AI-Based Methods): The Introduction and related work sections are currently incomplete. Please include recent RIS literature, including but not limited to: Basar et al., IEEE Access, 2019, Yildirim et al., IEEE TCOM, 2020, Ghavidel Aghdam et al., IEEEMECOM, 2024, Tang et al., IEEE TCOM, 2022, Recent RIS statistical and path-loss modeling works (e.g., indoor/outdoor, mmWave) Expand the discussion on channel modeling and path-loss assumptions, which are critical in RIS systems. Clearly justify the adopted model and parameters with references. Importantly, the manuscript must discuss AI-based and reinforcement-learning-based RIS and hybrid beamforming approaches, which are now major competing methodologies.*

Authors' Response: We appreciate the Editor for this useful comment. We have addressed all these concerns by each Reviewer's comment and included appropriate information in the revised manuscript accordingly.

Following this recommendation, we have revised the Introduction to incorporate the suggested recent RIS works in order to better reflect the latest developments in advanced RIS architectures and space–air–ground integrated networks.

Specifically, we have added a new discussion paragraph in the Introduction (see the revised manuscript, highlighted in blue) that now cites the recommended works and positions them within the broader context of multi-layer RIS, self-powered RIS, refracting RIS, and RIS-enabled massive access. The added text reads:

> Reconfigurable intelligent surfaces (RISs) have emerged as a key enabler of this paradigm shift by transforming the wireless environment into a programmable entity [3]. Comprehensive surveys have established RIS as a fundamental building block of smart radio environments, capable of reshaping wireless propagation to support emerging 6G services [4, 5]. Early RIS research primarily focused on enhancing signal strength and spectral efficiency. More recent studies reposition RISs as multi-functional and reliability-critical platforms that integrate communication, sensing, and wireless power transfer [6, 7]. In parallel, advanced RIS architectures, including multi-layer, refracting, and self-powered designs, have been investigated to support satellite–terrestrial and space–air–ground integrated networks [8 – 10]. For instance, multi-layer stacking RIS receivers have been studied for high-altitude platform (HAP)-enabled simultaneous wireless information and power

> transfer (SWIPT) networks [11], while self-powered adaptive RIS designs have been considered for secure satellite-terrestrial integration [12]. Refracting RIS has also been investigated for hybrid satellite-terrestrial relay systems [13]. More recently, air-driven seamless and massive access has been discussed for space-air-ground integrated networks, where aerial platforms (e.g., unmanned aerial vehicle (UAV) and HAP tiers) play a coordinating role to enable wide-area continuity of access and large-scale user connectivity across heterogeneous layers [14]. These developments are consistent with broader 6G visions that emphasize scalable and intelligent connectivity across heterogeneous network layers [15].

(i) Fairness-related performance gap. The revised Introduction now explicitly states that, in RIS-assisted mmWave systems, user-rate imbalance is often *structural rather than incidental*. Due to severe blockage sensitivity, geometry-dependent path loss, and the passive nature of RIS reflections, users with unfavorable propagation conditions may experience near-zero instantaneous rates. However, conventional sum-rate maximization tolerates such rate collapse as long as the aggregate throughput is increased, leading to unreliable service for vulnerable users.

To make this gap explicit, we have added the following discussion (highlighted in blue in the revised Introduction):

> Recent path-loss measurements and channel modeling studies reveal that RIS-assisted mmWave links exhibit pronounced geometry-dependent attenuation and user-specific disparities, particularly in indoor and outdoor deployments [16,17]. Furthermore, statistical characterizations of RIS-assisted channels demonstrate non-trivial SNR distributions across near- and far-field regimes, which further intensify rate imbalance among users [18]. These model- and measurement-driven observations indicate that channel disparity in RIS-assisted mmWave systems is often structural rather than incidental, making fairness and service reliability first-order design challenges rather than secondary performance metrics.

(ii) When this gap becomes critical. The revised Introduction further clarifies that this fairness gap becomes particularly critical in *blockage-prone and quasi-static RIS-assisted mmWave scenarios*, where scheduling diversity is limited and fairness cannot be achieved through long-term averaging. In such settings, sum-rate-based designs may repeatedly favor users with strong composite channels, effectively starving blocked or cell-edge users.

This limitation is explicitly highlighted in the following added passage:

> Although these throughput-centric approaches improve aggregate spectral efficiency, they systematically favor users with strong composite channels and may severely degrade the service quality of cell-edge or blocked users. This limitation is particularly pronounced in RIS-assisted mmWave systems, where blockage and path-loss disparities are inherently amplified by passive reflection mechanisms.

We agree that the original Introduction did not sufficiently contextualize our model-based framework against recent AI/DRL directions for RIS and hybrid beamforming:

(1) Added AI/DRL discussion and positioning: In the revised manuscript, we added a dedicated discussion in the Introduction to acknowledge AI/DRL-based RIS/precoding methods and to state, in concrete terms, why we adopt a model-based approach for fairness-critical designs. The added text is *highlighted in blue* and reads:

> In parallel, artificial intelligence (AI) and deep reinforcement learning (DRL) techniques have been actively explored for RIS and hybrid beamforming optimization [21,29]. Although these data-driven approaches exhibit strong adaptability in complex environments, they typically require large training datasets, lack interpretability, and do not provide explicit fairness guarantees. Model-based optimization frameworks therefore remain attractive for fairness-critical systems, as they offer transparent performance control, stable convergence behavior, and direct incorporation of hardware constraints. Accordingly, this work adopts a model-based design philosophy that complements, rather than replaces, AI-based RIS optimization.

(2) Added a comparison table: Following the Reviewer's suggestion, we have added:

> Table 1 highlights how the proposed framework uniquely addresses fairness and service reliability under practical hardware constraints when compared with classical and AI/DRL-based methods

Consider 4 – *Computational Complexity and Convergence Claims: Claims of improved efficiency and convergence must be better substantiated. Provide a computational complexity analysis in Big- notation and/or in terms of dominant multiplications/additions.*

Authors' Response: We appreciate the Editor for this useful comment. We have addressed all these concerns by each Reviewer's comment and included appropriate information in the revised manuscript accordingly.

To analyze the computational complexity of the proposed joint hybrid beamforming and RIS phase shifts design, we note that the total runtime of the optimization procedure is given by sum of computational costs of the proposed Algorithm. 1 and Algorithm. 2. We will analyse the complexity of each algorithm as follows:

- **Algorithm.** 1: We consider the pseudo code of Algorithm 1:

  We recall the update formulation of $\mathbf{Q}^{(\kappa)}$ is given by using the Lagrangian multiplier method as follows:

$$\mathbf{q}_k^{(\kappa+1)} = \begin{cases} \left(\Psi^{(\kappa)}\right)^{-1} \rho_k^{(\kappa)} \mathbf{y}^{(\kappa)}, & \text{if } \left\|\mathbf{Q}^{(\kappa+1)}\right\|^2 \leq P \\ \left(\Psi^{(\kappa)} + \mu I_M\right)^{-1} \rho_k^{(\kappa)} \mathbf{y}^{(\kappa)}, & \text{otherwise} \end{cases} \tag{1}$$

  with $\mu > 0$ is a constant selected to satisfy the following equality by the bisection method:

$$\sum_{k=1}^{K} \left\| \left(\Psi^{(\kappa)} + \mu I_M\right)^{-1} \rho_k^{(\kappa)} b_k^{(\kappa)} \right\|^2 = P. \tag{2}$$

Algorithm 1 Proposed Algorithm for problem (13)

Require: h_k, $\mathbf{G}$, σ_k, $\mathbf{\Theta}^{(0)}$, $\mathbf{Q}^{(0)}$

while $\left|G^{(\kappa-1)} - G^{(\kappa)}\right| \geq \epsilon_g$ **do**

  Update $\mathbf{Q}^{(\kappa)}$ based on (16)

  Update $\mathbf{\Theta}^{(\kappa)}$ based on (18)

  $\kappa \leftarrow \kappa + 1$

end while

Ensure: Optimized variables $\mathbf{Q}_{\text{opt}}$, $\mathbf{\Theta}_{\text{opt}}$

We note that for any dense square matrix $A \in \mathbb{C}^{n\times n}$ the computation complexity to calculate its inverse is $\mathcal{O}(n^3)$ [7]. We also observe that the matrix product between two matrices $A \in \mathbb{C}^{n\times m}$ and $B \in \mathbb{C}^{m\times p}$ is $\mathcal{O}(mnp)$. Thus, the complexity to compute $\mathbf{Q}^{(\kappa+1)}$ is $\mathcal{O}(M^3K)$ for a fixed value of μ. However, to select the constant μ that satisfies the given condition, we need an additional iteration complexity of $\mathcal{O}(M^3K/\log(\epsilon_\mu))$, where ϵ_μ is the target accuracy of the bisection method, since this method is $\mathcal{O}(\frac{1}{\log(\epsilon_\mu)})$ [8] and at each iteration we need to calculate the inverse and matrix multiplication of $\mathcal{O}(M^3K)$. Consider the complexity to update $\mathbf{\Theta}^{(\kappa)}$, we recall the equation:

$$\theta_f^{(\kappa+1)} = -\angle\Delta_f^{(\kappa)}, f \in \mathcal{F}. \tag{3}$$

By the definition of $\Delta_f^{(\kappa)}$, the computation complexity is $\mathcal{O}(K^2F^2 + FM)$. The complexity of a two-block BCD algorithm can be shown to have a complexity of $\mathcal{O}(\frac{1}{\sqrt{\epsilon_g}})$ [9]. Thus, the complexity of Algorithm. 1 is $\mathcal{O}((\frac{M^3K}{\log(\epsilon_\mu)} + K^2F^2 + FM)\frac{1}{\sqrt{\epsilon_g}})$.

- **Algorithm.** 2: We then consider the pseudo code of Algorithm 2:

  Similar to the analysis for Algorithm. 1, we show the complexity to compute $\mathbf{W}^{(\eta)}$ to be $\mathcal{O}(MNK)$ while the RCG algorithm for update $\mathbf{V}^{(\eta)}$ is $\mathcal{O}(\epsilon_v^{-2})$ where ϵ_v^{-2} is the target accuracy of RCG. Since the matrix decomposition procedure leverage two-block BCD, its complexity is $\mathcal{O}((MNK + \epsilon_v^{-2})\frac{1}{\sqrt{\epsilon_q}})$. Hence, the total complexity of Algorithm. 2 is $\mathcal{O}((\frac{M^3K}{\log(\epsilon_\mu)} + K^2F^2 + FM)\frac{1}{\sqrt{\epsilon_g}} + (MNK + \epsilon_v^{-2})\frac{1}{\sqrt{\epsilon_q}})$.

We have added the following discussion in the revised manuscript:

> *Complexity Analysis of Alg. 2: To analyze the computational complexity of the proposed joint hybrid beamforming and RIS phase shifts design, it is essential to compute the complexity of Alg. 1 along with the complexity of RCG algorithm. We note that for any dense square matrix $A \in \mathbb{C}^{n\times n}$ the computation complexity to calculate its inverse is $\mathcal{O}(n^3)$ [37]. We also observe that the matrix product between two matrices $A \in \mathbb{C}^{n\times m}$ and $B \in \mathbb{C}^{m\times p}$*

Algorithm 2 Proposed Algorithm for problem (12)

Require: $\mathbf{h}_k$, $\mathbf{G}$, σ_k

1: Initialize $\mathbf{\Theta}^{(0)} = I_{F\times F}$ and $\mathbf{Q}^{(0)}$ by (32)
2: Acquire $\mathbf{\Theta}_{\text{opt}}$ and $\mathbf{Q}_{\text{opt}}$ based on Alg.1
3: **while** $\left\|\mathbf{Q}_{\text{opt}} - \mathbf{V}^{(\eta-1)}\mathbf{W}^{(\eta-1)}\right\|_{\mathcal{F}}^2 \geq \epsilon_q$ **do**
4: Update $\mathbf{W}^{(\eta)}$ by applying (20)
5: Update $\mathbf{V}^{(\eta)}$ based on vectorization (25) and apply RCG algorithm (27) until convergence
6: $\eta \leftarrow \eta + 1$
7: **end while**
8: Remap each RIS phase shifter b_f to the nearest element in $\mathcal{S}_r$
9: Remap each non-zero element of $\mathbf{V}$ to the nearest element in $\mathcal{S}_a$

Ensure: Optimized variables $\mathbf{V}$ and $\mathbf{W}$

is $\mathcal{O}(mnp)$. Thus, the complexity of (16) is $\mathcal{O}(M^3K)$ for a fixed value of μ. However, to select the constant μ, we need an additional iteration complexity of $\mathcal{O}(M^3K/\log(\epsilon_\mu))$, where ϵ_μ is the target accuracy of the bisection method, since this method is $\mathcal{O}(\frac{1}{\log(\epsilon_\mu)})$ [38] and at each iteration we need to calculate the inverse and matrix multiplication of $\mathcal{O}(M^3K)$. Next, we consider the complexity of updating $\mathbf{\Theta}^{(\kappa)}$. We recall the definition of $\Delta_f^{(\kappa)}$ as (18), which is $\mathcal{O}(K^2F^2 + FM)$. The complexity of a two-block BCD algorithm has a complexity of $\mathcal{O}(\frac{1}{\sqrt{\epsilon_g}})$ [39]. Thus, the complexity of Alg. 1 is $\mathcal{O}((\frac{M^3K}{\log(\epsilon_\mu)} + K^2F^2 + FM)\frac{1}{\sqrt{\epsilon_g}})$. Similarly, we show the complexity to compute $\mathbf{W}^{(\eta)}$ to be $\mathcal{O}(MNK)$ while the RCG algorithm for update $\mathbf{V}^{(\eta)}$ is $\mathcal{O}(\epsilon_v^{-2})$, where ϵ_v^{-2} is the target accuracy of RCG. Since our beam decomposition leverages two-block BCD, its complexity is $\mathcal{O}((MNK + \epsilon_v^{-2})\frac{1}{\sqrt{\epsilon_q}})$. Hence, the total complexity of Alg. 2 is $\mathcal{O}((\frac{M^3K}{\log(\epsilon_\mu)} + K^2F^2 + FM)\frac{1}{\sqrt{\epsilon_g}} + (MNK + \epsilon_v^{-2})\frac{1}{\sqrt{\epsilon_q}})$.

Consider 5 – *Practical Relevance and Deployment Considerations: The manuscript motivates OSA and low-resolution RIS phases but lacks concrete system-level interpretation.*

Authors' Response: We appreciate the Editor for this useful comment. We have addressed all these concerns by each Reviewer's comment and included appropriate information in the revised manuscript accordingly.

(1) OSA-based RIS hybrid beamforming is particularly attractive in realistic mmWave deployment scenarios where hardware cost, energy efficiency, and robustness to blockage are primary concerns. One representative example is indoor hotspot expansion, such as shopping malls, airports, and large office buildings, where mmWave links suffer from severe penetration loss and frequent human blockage. In such environments, OSA hybrid architectures significantly reduce the number of phase shifters and RF interconnections compared to fully-connected structures, thereby lowering hardware complexity and power consumption while preserving sufficient data rate [10, 11]. When combined with RIS panels deployed

on walls or ceilings, controllable reflected paths can be created to enhance coverage in non-line-of-sight (NLoS) regions, which aligns with standardized indoor hotspot channel models defined in 3GPP TR 38.901 standard. Another practical deployment scheme is for industrial automation and smart factories, where metallic structures, moving robots, and machinery frequently block line-of-sight links. In such settings, reliability and low latency are more critical than extreme spectral efficiency. As such, OSA hybrid beamforming provides a hardware-efficient solution with reduced calibration complexity, while RIS-assisted propagation control can dynamically redirect signals around obstacles to maintain link robustness. Thus, this deployment perspective is consistent with IMT-2020 requirements and industrial 5G use case scenarios.

(2) From a practical implementation perspective, the control overhead and calibration requirements of the proposed OSA-based RIS hybrid architecture remain manageable. For RIS phase updates, configuration signaling is typically performed over a low-rate control link and updated at the channel coherence time scale. Since practical RIS implementations employ discrete phase shifts with limited resolution, the control payload scales linearly with the number of elements and phase quantization bits, which helps constrain signaling overhead [12, 13]. Moreover, element grouping or codebook-based reflection design can further reduce configuration complexity.

For the OSA analog precoder, the subarray structure significantly reduces the number of phase shifters and RF interconnections compared to fully-connected architectures, thereby simplifying RF calibration and hardware synchronization [14, 11]. Because each RF chain only drives a subset of antennas, the dimensionality of analog beam training and feedback is also reduced, leading to lower beam-search overhead. Consequently, the combined OSA-RIS architecture achieves a favorable trade-off between performance and implementation complexity, making it suitable for practical mmWave deployments.

We have added the following discussion in the Introduction of the revised manuscript:

> Meanwhile, OSA-based RIS hybrid beamforming is particularly suitable for practical mmWave deployments where blockage is a major concern, such as indoor hotspots in shopping malls, airports, and large office buildings. In these scenarios, severe penetration loss and frequent human blockage degrade mmWave links. Compared to fully connected architectures, the OSA structure reduces the number of phase shifters and RF interconnections, thereby lowering hardware complexity and power consumption while maintaining satisfactory data rates. Moreover, the control and calibration overhead remains manageable, as RIS phase updates are performed over a low-rate control link at the channel coherence time scale, and with discrete phase shifts of limited resolution, the signaling payload scales linearly with the number of elements and quantization bits.

Consider 6 – *Notation, Organization, and English Quality*

Authors' Response: We appreciate the Editor for this useful comment. We have addressed all these concerns by each Reviewer's comment and included appropriate information in the revised manuscript accordingly.

(1) The manuscript has been thoroughly revised to improve clarity and readability. The revised passages are explicitly **highlighted in blue** in the submitted manuscript to clearly indicate where sentence structures have been simplified and split for improved clarity.

(2) All symbols and technical terms are now explicitly defined at their first occurrence. In addition, ambiguous pronouns such as "this," "that," and "these" have been replaced with precise references to the corresponding variables, equations, or algorithmic steps to eliminate unclear antecedents.

(3) A careful language pass has been conducted across the entire manuscript to correct minor grammatical inconsistencies, including article usage and subject–verb agreement, and to ensure consistent technical terminology and formatting (e.g., "fully-digital," "hybrid beamforming," and "phase-shifter").

We believe that these revisions substantially improve the clarity, precision, and overall readability of the manuscript.

Once again, we would like to thank the Editor for your thoughtful comments, which are particularly useful to enhance the quality of this paper. We hope that you can find our explanations/justifications and revisions/improvements satisfactory. Thank you!

Reviewer 1

General comments: *The paper studies a reconfigurable intelligent surface aided downlink with hybrid beamforming under an overlapped sub array architecture, and it maximizes the geometric mean of user rates by alternating between a fully digital surrogate precoder and RIS phases, while enforcing unit modulus and OSA connectivity. The method uses a pseudoinverse based warm start for precoder, closed form RIS updates, and a normalized least squares update followed by Riemannian conjugate gradient steps. I have the following major comments:*

Authors' Response: We thank the Reviewer for the thorough evaluation of our manuscript and for the precise synthesis of the proposed framework. We appreciate the Reviewer's recognition of the core algorithmic contributions, including the interference-aware, pseudoinverse-based warm-start initialization, the closed-form optimization of the RIS phase shifts, and the use of Riemannian conjugate gradient (RCG) manifold optimization to enforce unit-modulus and overlapped sub-array (OSA) constraints. We fully concur with the Reviewer's characterization of these methodological components. In the following, we provide detailed responses to the major comments, with particular emphasis on clarifying the practical scalability of the OSA architecture and on substantiating the effectiveness of the geometric mean (GM) rate objective in promoting user fairness in the considered RIS-assisted downlink system.

Reviewer Point 1.1 — *The introduction lacks some coherence. Please solidify. Moreover, there are many previous related RIS works such as [REF01,REF02]. Kindly include.*

Authors' Response: We thank Reviewer 1 for pointing out the omission of several relevant RIS-related works in the Introduction. In response, we have explicitly incorporated additional references on multi-functional RIS and RIS-enabled integrated sensing and communication (ISAC), including [REF01] and [REF02], to strengthen the contextual background and ensure better coverage of recent RIS research directions.

[6] "Multi-Functional RIS for a Multi-Functional System: Integrating Sensing, Communication, and Wireless Power Transfer," in IEEE Network.

[7] "RIS-Enabled Integrated Sensing and Communication for 6G Systems," 2024 IEEE Wireless Communications and Networking Conference (WCNC), Dubai, United Arab Emirates, 2024, pp. 1-6.

Specifically, the following revised passage has been added to the Introduction (highlighted in blue in the manuscript):

> Reconfigurable intelligent surfaces (RISs) have emerged as a key enabler of this paradigm shift by transforming the wireless environment into a programmable entity [3]. Comprehensive surveys have established RIS as a fundamental building block of smart radio environments, capable of reshaping wireless propagation to support emerging 6G services [4, 5]. Early RIS research primarily focused on enhancing signal strength and

> spectral efficiency. More recent studies reposition RISs as multi-functional and reliability-critical platforms that integrate communication, sensing, and wireless power transfer [6, 7]. In parallel, advanced RIS architectures, including multi-layer, refracting, and self-powered designs, have been investigated to support satellite–terrestrial and space–air–ground integrated networks [8 – 10]. For instance, multi-layer stacking RIS receivers have been studied for high-altitude platform (HAP)-enabled simultaneous wireless information and power transfer (SWIPT) networks [11], while self-powered adaptive RIS designs have been considered for secure satellite-terrestrial integration [12]. Refracting RIS has also been investigated for hybrid satellite-terrestrial relay systems [13]. More recently, air-driven seamless and massive access has been discussed for space-air-ground integrated networks, where aerial platforms (e.g., unmanned aerial vehicle (UAV) and HAP tiers) play a coordinating role to enable wide-area continuity of access and large-scale user connectivity across heterogeneous layers [14]. These developments are consistent with broader 6G visions that emphasize scalable and intelligent connectivity across heterogeneous network layers [15].

Reviewer Point 1.2 — *Please discuss the novelty behind the optimization of the linear transmit beamformers and the RIS reflecting coefficients.*

Authors' Response: Thank you for requesting clarification on the novelty of our optimization design for (i) the linear transmit beamformers and (ii) the RIS reflecting coefficients. We would like to clarify that the corresponding novelty discussions have been explicitly revised and highlighted in blue in the Introduction, as detailed below.

(i) Fairness-driven optimization of linear transmit beamformers. The motivation for departing from sum-rate maximization and adopting the geometric mean (GM) rate objective to mitigate blockage- and path-loss-induced rate imbalance is explicitly discussed in the revised Introduction. In particular, the following blue-highlighted passages directly reflect this contribution:

> Geometric mean (GM) rate maximization provides a principled mechanism to address this fairness and reliability gap by explicitly balancing individual user rates [28]. Unlike sum-rate objectives, which are insensitive to individual rate collapse, GM-rate penalizes vanishing user rates and enforces balanced performance across all users within a single transmission interval. This property is particularly important in quasi-static RIS-assisted mmWave systems, where limited scheduling diversity and reliance on reflected paths prevent fairness from being achieved through long-term averaging.

In addition, the interference-aware warm-start strategy and its impact on convergence are explicitly highlighted in the contributions list of the Introduction:

> We develop an interference-aware warm-start strategy that stabilizes the power-constrained precoder update and accelerates convergence by up to 18.18% compared with random initialization.

These passages collectively reflect the novelty of the proposed precoder optimization in terms of both fairness-driven objective design and stabilized interference-aware initialization.

ii) Hardware-constrained optimization of RIS reflecting coefficients. The novelty of the RIS phase optimization and its realization under practical hardware constraints is also explicitly framed in the revised Introduction. In particular, the following blue-highlighted contribution statement directly corresponds to the second item raised by the Reviewer:

> We propose an OSA-consistent Riemannian manifold factorization that maps the fully-digital GM-optimal precoder benchmark into a realizable hybrid analog–digital structure without resorting to matrix lifting or semidefinite relaxation.

Furthermore, the necessity of enforcing unit-modulus, discrete phase control, and OSA connectivity as part of the system design is emphasized earlier in the Introduction through repeated references to OSA-based hybrid beamforming and discrete RIS phases, ensuring that the RIS optimization is positioned as a hardware-feasible realization rather than an idealized fully-digital solution.

> *We hope that the revised and highlighted discussions in the Introduction clarify the novelty of the proposed framework to the Reviewer's satisfaction.*

Reviewer Point 1.3 — *The choice of geometric mean of rates is not well motivated. In particular, the objective is to maximize* $\left(\prod_{k=1}^{K} R_k\right)$ *with* $R_k = \log_2(1 + \mathrm{SINR}_k)$ *in (12). Why is the geometric mean taken over the rates and not over* $1 + \mathrm{SINR}_k$ *? The objective* $\sum_k \log R_k$ *weights users through* $\log\log(1 + \mathrm{SINR}_k)$*, which compresses differences more than* $\sum_k \log(1 + \mathrm{SINR}_k)$*. Explain this choice better aligns with your fairness target than the standard proportional fairness form or other types of fairness objectives.*

Authors' Response: We would like to thank Reviewer 1 for this comment.

The novelty behind choosing the geometric mean (GM) of data rates as the objective function lies in the trade-off of rate fairness among users with the overall network performance. Specifically, the overall network performance is often formulated as the weighted sum rate (SR) optimization problem in the literature [1][2]. However, this approach has the deficiency of allocating a large fraction of the sum-rate to a few users having good channel conditions, while leaving the rest of the users with almost zero rates. This motivates the formulation of fairness based on GM rate due to several key aspects:

- The geometric mean over $\prod_{k=1}^{K}(1 + \mathrm{SINR}_k)$ is equivalent to the unweighted sum rate formulation, i.e. $\sum_{k=1}^{K} R_k$, since $\sum_{k=1}^{K} \log_2(1 + \mathrm{SINR}_k) = \log_2\left(\prod_{k=1}^{K}(1 + \mathrm{SINR}_k)\right)$. As such, the objective function $\max(\sum_{k=1}^{K} R_k) = \max \log_2\left(\prod_{k=1}^{K}(1 + \mathrm{SINR}_k)\right)$ is equivalent to $\max\left(\prod_{k=1}^{K}(1 + \mathrm{SINR}_k)\right)$.

- To achieve the fairness target, one can employ the max-min rate objective or proportional rate constraint for maximizing the rate of the users having poor channel conditions. However, the max-min rate approach leads to an undesirable degradation in the overall sum rate (SR) of the system while the proportional rate constraint introduces additional nonconvexity and computation complexity to the hybrid beamforming scheme. Furthermore, the authors in [3] pointed out that with suitable choice of parameters, the standard proportional fairness can be regarded as a max-min optimization problem. As an alternative solution to balance fairness and high sum rate while introduce little computation difficulty, the geometric mean (GM) rate maximization is proposed as a promising solution in the literature [4][5][6].

We have added the following discussion in the revised manuscript:

> Existing works in the literature often use the sum-rate method and the max-min method to quantify the quality of service (QoS) of the network, each of the formulation in favor the overall rates or resource fairness across users, respectively. To balance this trade-off between total QoS and resource fairness, proportional fairness constraint is introduced as an alternative method, at the cost of additional complexity of non-convex constraints [33]. Thus, the recent GM rate maximization method overcomes this challenge by considering a different objective function [28].

> *We hope that the revised and highlighted discussions on the choice of geometric mean clarify the proposed framework to the Reviewer's satisfaction.*

Reviewer Point 1.4 — *Explain how discrete phases are enforced consistently across both blocks and how per iteration the discrete phases are activated.*

Authors' Response:

We thank the Reviewer for this comment regarding the enforcement of discrete phase constraints. As stated in Algorithm 2 of the manuscript, the discrete phases of both the RIS and the analog beamformer are enforced through a projection step following each continuous optimization update. Specifically, at each iteration, we first obtain the digital beamformer $\mathbf{W}$ and the continuous-valued analog beamformer $\mathbf{V}$ by solving the matrix factorization problem using the Riemannian conjugate gradient (RCG) algorithm. The resulting continuous-phase solutions are then mapped to their discrete counterparts.

For the RIS, after obtaining the optimized continuous coefficient b_f^{opt} of $\mathbf{\Theta}_{\text{opt}}$, we compute the distance between b_f^{opt} and each candidate phase in the discrete set $\mathcal{S}_r$, and project b_f^{opt} onto the element in $\mathcal{S}_r$ with the smallest distance. The discrete analog beamforming coefficients are obtained in the same manner by projecting the continuous-valued entries of $\mathbf{V}$ onto the corresponding discrete phase set. To make this procedure explicit, we have added the following discussion and highlighted it in blue after **Algorithm 2 (page 14)** of the revised manuscript:

Remark on discrete phase activation: At each iteration of Algorithm 2, the continuous-phase solutions of both the RIS and the analog beamformer are immediately projected onto their corresponding discrete phase sets. Specifically, each RIS phase coefficient is mapped to the nearest element in $\mathcal{S}_r$, and each non-zero entry of the analog beamforming matrix is projected onto the discrete phase set $\mathcal{S}_a$. As a result, the discrete phase constraints are enforced per iteration, ensuring that all intermediate solutions remain hardware-feasible throughout the alternating optimization.

This added remark clarifies that the discrete phases are activated at every iteration and are enforced consistently across both optimization blocks, rather than being applied only after convergence.

We hope that this clarification and the corresponding revision satisfactorily address the Reviewer's concern.

Reviewer Point 1.5 — *In (14), please provide the exact inequality or tangent majorizer that turns $\sum_k \log R_k$ into this weighted sum at each step that can guarantee all regularity conditions.*

Authors' Response: We would like to thank Reviewer 1 for this comment.

To transform the geometric mean objective into the weighted sum rate at each step of the algorithm, we adopt multiple steps of approximation as follows [6]. Consider the objective function:

$$\max_{\mathbf{Q},\mathbf{\Theta}} \quad G(\mathbf{Q},\mathbf{\Theta}) = \prod_{k=1}^{K} (R_k(\mathbf{Q},\mathbf{\Theta}))^{\frac{1}{K}} \tag{4}$$

It is plausible to equivalently transform (4) to the following one:

$$\min_{\mathbf{Q},\mathbf{\Theta}} f(R_1(\mathbf{Q},\mathbf{\Theta})),\ldots,R_K(\mathbf{Q},\mathbf{\Theta}))) \triangleq \frac{1}{\left(\prod_{k=1}^{K} R_k(\mathbf{Q},\mathbf{\Theta})\right)^{\frac{1}{K}}} \tag{5}$$

Notice the function $f(R_1(\mathbf{Q},\mathbf{\Theta})),\ldots,R_K(\mathbf{Q},\mathbf{\Theta})))$ is the composition of the convex function $f(R_1,\ldots,R_K) = 1/(\prod_{k=1}^{K} R_k)^{1/K}$ and the non-convex functions $R_k(\mathbf{Q},\mathbf{\Theta})$, $k = 1,\ldots,K$. Let $(\mathbf{Q}^{(\kappa)},\mathbf{\Theta}^{(\kappa)})$ be a feasible point that is found from the $(\kappa-1)$ iteration. We then introduce the linearized function of (5) at $(\mathbf{Q}^{(\kappa)},\mathbf{\Theta}^{(\kappa)})$ by the Taylor's expansion as:

$$2f\left(R_1^{(\kappa)},\ldots,R_K^{(\kappa)}\right) - f\left(R_1^{(\kappa)},\ldots,R_K^{(\kappa)}\right)\frac{1}{K}\sum_{k=1}^{K}\frac{R_k(\mathbf{Q},\mathbf{\Theta})}{R_k^{(\kappa)}}, \tag{6}$$

where $R_k^{(\kappa)}$, $k = 1,\ldots,K$ denotes the rate of user k evaluated at $(\mathbf{Q}^{(\kappa)},\mathbf{\Theta}^{(\kappa)})$. Since we have $f\left(R_1^{(\kappa)},\ldots,R_K^{(\kappa)}\right) > 0$, we can generate the next feasible point $(\mathbf{Q}^{(\kappa+1)},\mathbf{\Theta}^{(\kappa+1)})$ from the following equivalent problem:

$$\max_{\mathbf{Q},\mathbf{\Theta}} \frac{1}{K}\sum_{k=1}^{K}\frac{R_k(\mathbf{Q},\mathbf{\Theta})}{R_k^{(\kappa)}} f\left(R_1^{(\kappa)},\ldots,R_K^{(\kappa)}\right) \tag{7}$$

which is equivalent to the following problem:

$$\max_{\mathbf{Q},\mathbf{\Theta}} \hat{G}(\mathbf{Q},\mathbf{\Theta}) \triangleq \sum_{k=1}^{K}\rho_k^{(\kappa)} R_k(\mathbf{Q},\mathbf{\Theta}) \tag{8}$$

for $\rho_k^{(\kappa)} \triangleq \frac{f\left(R_1^{(\kappa)},\ldots,R_K^{(\kappa)}\right)}{R_k^{(\kappa)}}, \quad k=1,\ldots,K$ is the weight for each user's rate at the iteration κ.

We have added the following discussion in the revised manuscript:

> We note that the maximize the objective function $G(\mathbf{Q},\mathbf{\Theta})$ of (13) is equivalent to minimize $1/G(\mathbf{Q},\mathbf{\Theta})$ subject to (13b) and (13c). Notice that the function $1/G(\mathbf{Q},\mathbf{\Theta})$ is the composition of the convex function $f(R_1,\ldots,R_K) = 1/(\prod_{k=1}^{K} R_k)^{1/K}$ and the non-convex functions $R_k(\mathbf{Q},\mathbf{\Theta}),\ k=1,\ldots,K$. We then consider the linearization of $1/G(\mathbf{Q},\mathbf{\Theta})$ around $(\mathbf{Q}^{(\kappa)},\mathbf{\Theta}^{(\kappa)})$, a feasible point that is found from the $(\kappa-1)$ iteration. Thus, we apply a first-order Taylor approximation at each BCD iteration κ, transforming problem (13) into the form $2f\left(R_1^{(\kappa)},\ldots,R_K^{(\kappa)}\right) - f\left(R_1^{(\kappa)},\ldots,R_K^{(\kappa)}\right)\frac{1}{K}\sum_{k=1}^{K}\frac{R_k(\mathbf{Q},\mathbf{\Theta})}{R_k^{(\kappa)}}$, where $R_k^{(\kappa)},\ k=1,\ldots,K$ denotes the rate of user k evaluated at $(\mathbf{Q}^{(\kappa)},\mathbf{\Theta}^{(\kappa)})$. Since we have $f\left(R_1^{(\kappa)},\ldots,R_K^{(\kappa)}\right) > 0$, we can generate the next feasible point $(\mathbf{Q}^{(\kappa+1)},\mathbf{\Theta}^{(\kappa+1)})$ from the following equivalent problem:
>
> $$\max_{\mathbf{Q},\mathbf{\Theta}} \quad \hat{G}(\mathbf{Q},\mathbf{\Theta}) \triangleq \sum_{k=1}^{K}\rho_k^{(\kappa)} R_k(\mathbf{Q},\mathbf{\Theta}) \tag{14a}$$
>
> $$\text{s.t.} \quad (13b), (13c), \tag{14b}$$

> *We hope that this clarification and the corresponding revision satisfactorily address the Reviewer's concern.*

Reviewer Point 1.6 — *Discuss the relation between the surrogate objective and fairness across users. In particular, Fig. 4 to Fig. 7 plot the GM Rate. Since GM can remain high even when one user is small, it would be useful to add a plot of the worst user rate and the median user rate for the same simulation to confirm that your GM design indeed lifts the weakest user relative to FC and NOSA.*

Authors' Response: We would like to thank Reviewer 1 for this comment.

We have added the plot of the worst user rate and the median user rate for the GM hybrid beamforming simulation relative to FC and NOSA configuration. For simplicity, we consider

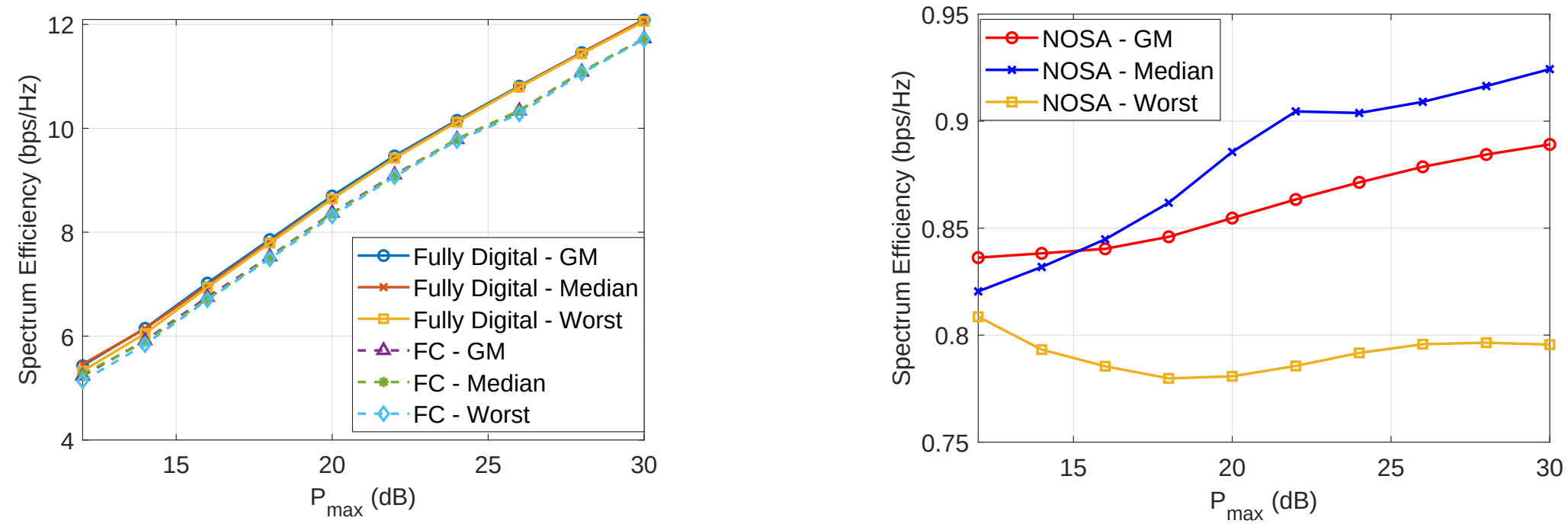

(a) Rate Distribution of NOSA architecture. (b) Rate Distribution of NOSA architecture.

Figure 1: Median Rate vs Worst Rate comparison for maximum GM design

the limited-bit parameters set up of $Q_1 = Q_2 = \infty$ for both FC and NOSA design. The simulation results can be observed in Fig. 1:

As shown in Fig. 1a, the geometric mean rate aligns closely with the median user rate and worst user rate for the fully digital and FC schemes, verifying the fairness objective across users. However, Fig. 1b shows a considerable separation between the three benchmarks. This phenomenon results from the trade-off between architecture complexity and QoS performance of NOSA, which introduces little degree of freedom for the analog beamforming. Hence, the nature of the NOSA scheme strengthens the dilemma between the overall rate and fairness across user, leading to the non-alignment between the GM rate and individual rate of users.

We have added the following discussion in the revised manuscript:

> Fig. 6 illustrates the relationship between the surrogate objective and user fairness. Specifically, we evaluate the worst-user rate and the median-user rate under the same simulation settings to verify that the GM design effectively improves the performance of the weakest user relative to FC and NOSA. As shown in Fig. 6a, the performance curve of each target user aligns to the geometric mean curve for both FD and FC architectures, confirming the fairness traits of GM design. However, Fig. 6b shows a considerable separation between the three benchmarks. This phenomenon arises from the trade-off between architectural complexity and QoS performance in the NOSA scheme, which provides a limited degree of freedom for analog beamforming to suppress inter-user interference. Consequently, the inherent structure of NOSA intensifies the trade-off between overall rate and user fairness, leading to a misalignment between the GM rate and the individual user rates.

> *We hope that this clarification and the corresponding revision satisfactorily address the Reviewer's concern.*

Reviewer Point 1.7 — *The computational complexity should be analyzed in terms of number of additions and multiplications or in terms of big-Oh notation.*

Authors' Response: We would like to thank Reviewer 1 for this interesting comment.

To analyze the computational complexity of the proposed joint hybrid beamforming and RIS phase shifts design, we note that the total runtime of the optimization procedure is given by sum of computational costs of the proposed Algorithm. 1 and Algorithm. 2. We will analyse the complexity of each algorithm as follows:

- **Algorithm.** 1: We consider the pseudo code of Algorithm 1:

Algorithm 1 Proposed Algorithm for problem (13)

Require: $\mathbf{h}_k$, $\mathbf{G}$, σ_k, $\mathbf{\Theta}^{(0)}$, $\mathbf{Q}^{(0)}$

1: **while** $\left|G^{(\kappa-1)} - G^{(\kappa)}\right| \geq \epsilon_g$ **do**

2: Update $\mathbf{Q}^{(\kappa)}$ based on (16)

3: Update $\mathbf{\Theta}^{(\kappa)}$ based on (18)

4: $\kappa \leftarrow \kappa + 1$

5: **end while**

Ensure: Optimized variables $\mathbf{Q}_{\text{opt}}$, $\mathbf{\Theta}_{\text{opt}}$

We recall the update formulation of $\mathbf{Q}^{(\kappa)}$ is given by using the Lagrangian multiplier method as follows:

$$\mathbf{q}_k^{(\kappa+1)} = \begin{cases} \left(\Psi^{(\kappa)}\right)^{-1} \rho_k^{(\kappa)} \mathbf{y}^{(\kappa)}, & \text{if } \left\|\mathbf{Q}^{(\kappa+1)}\right\|^2 \leq P \\ \left(\Psi^{(\kappa)} + \mu I_M\right)^{-1} \rho_k^{(\kappa)} \mathbf{y}^{(\kappa)}, & \text{otherwise} \end{cases} \tag{10}$$

with $\mu > 0$ is a constant selected to satisfy the following equality by the bisection method:

$$\sum_{k=1}^{K} \left\| \left(\Psi^{(\kappa)} + \mu I_M\right)^{-1} \rho_k^{(\kappa)} b_k^{(\kappa)} \right\|^2 = P. \tag{11}$$

We note that for any dense square matrix $A \in \mathbb{C}^{n\times n}$ the computation complexity to calculate its inverse is $\mathcal{O}(n^3)$ [7]. We also observe that the matrix product between two matrices $A \in \mathbb{C}^{n\times m}$ and $B \in \mathbb{C}^{m\times p}$ is $\mathcal{O}(mnp)$. Thus, the complexity to compute $\mathbf{Q}^{(\kappa+1)}$ is $\mathcal{O}(M^3K)$ for a fixed value of μ. However, to select the constant μ that satisfies the given condition, we need an additional iteration complexity of $\mathcal{O}(M^3K/\log(\epsilon_\mu))$, where ϵ_μ is the target accuracy of the bisection method, since this method is $\mathcal{O}(\frac{1}{\log(\epsilon_\mu)})$ [8] and at each iteration we need to calculate the inverse and matrix multiplication of $\mathcal{O}(M^3K)$. Consider the complexity to update $\mathbf{\Theta}^{(\kappa)}$, we recall the equation:

$$\theta_f^{(\kappa+1)} = -\angle\Delta_f^{(\kappa)}, f \in \mathcal{F}. \tag{12}$$

By the definition of $\Delta_f^{(\kappa)}$, the computation complexity is $\mathcal{O}(K^2F^2 + FM)$. The complexity of a two-block BCD algorithm can be shown to have a complexity of $\mathcal{O}(\frac{1}{\sqrt{\epsilon_g}})$ [9]. Thus, the complexity of Algorithm. 1 is $\mathcal{O}((\frac{M^3K}{\log(\epsilon_\mu)} + K^2F^2 + FM)\frac{1}{\sqrt{\epsilon_g}})$.

- **Algorithm.** 2: We then consider the pseudo code of Algorithm 2:

Algorithm 2 Proposed Algorithm for problem (12)

Require: $\mathbf{h}_k$, $\mathbf{G}$, σ_k

Initialize $\mathbf{\Theta}^{(0)} = I_{F\times F}$ and $\mathbf{Q}^{(0)}$ by (32)

Acquire $\mathbf{\Theta}_{\text{opt}}$ and $\mathbf{Q}_{\text{opt}}$ based on Alg.1

while $\left\|\mathbf{Q}_{\text{opt}} - \mathbf{V}^{(\eta-1)}\mathbf{W}^{(\eta-1)}\right\|_{\mathcal{F}}^2 \geq \epsilon_q$ **do**

  Update $\mathbf{W}^{(\eta)}$ by applying (20)

  Update $\mathbf{V}^{(\eta)}$ based on vectorization (25) and apply RCG algorithm (27) until convergence

  $\eta \leftarrow \eta + 1$

end while

Remap each RIS phase shifter b_f to the nearest element in $\mathcal{S}_r$

Remap each non-zero element of $\mathbf{V}$ to the nearest element in $\mathcal{S}_a$

Ensure: Optimized variables $\mathbf{V}$ and $\mathbf{W}$

Similar to the analysis for Algorithm. 1, we show the complexity to compute $\mathbf{W}^{(\eta)}$ to be $\mathcal{O}(MNK)$ while the RCG algorithm for update $\mathbf{V}^{(\eta)}$ is $\mathcal{O}(\epsilon_v^{-2})$ where ϵ_v^{-2} is the target accuracy of RCG. Since the matrix decomposition procedure leverage two-block BCD, its complexity is $\mathcal{O}((MNK + \epsilon_v^{-2})\frac{1}{\sqrt{\epsilon_q}})$. Hence, the total complexity of Algorithm. 2 is $\mathcal{O}((\frac{M^3K}{\log(\epsilon_\mu)} + K^2F^2 + FM)\frac{1}{\sqrt{\epsilon_g}} + (MNK + \epsilon_v^{-2})\frac{1}{\sqrt{\epsilon_q}})$.

We have added the following discussion in the revised manuscript:

Complexity Analysis of Alg. 2: To analyze the computational complexity of the proposed joint hybrid beamforming and RIS phase shifts design, it is essential to compute the complexity of Alg. 1 along with the complexity of RCG algorithm. We note that for any dense square matrix $A \in \mathbb{C}^{n\times n}$ the computation complexity to calculate its inverse is $\mathcal{O}(n^3)$ [37]. We also observe that the matrix product between two matrices $A \in \mathbb{C}^{n\times m}$ and $B \in \mathbb{C}^{m\times p}$ is $\mathcal{O}(mnp)$. Thus, the complexity of (16) is $\mathcal{O}(M^3K)$ for a fixed value of μ. However, to select the constant μ, we need an additional iteration complexity of $\mathcal{O}(M^3K/\log(\epsilon_\mu))$, where ϵ_μ is the target accuracy of the bisection method, since this method is $\mathcal{O}(\frac{1}{\log(\epsilon_\mu)})$ [38] and at each iteration we need to calculate the inverse and matrix multiplication of $\mathcal{O}(M^3K)$. Next, we consider the complexity of updating $\mathbf{\Theta}^{(\kappa)}$. We recall the definition of $\Delta_f^{(\kappa)}$ as (18), which is $\mathcal{O}(K^2F^2 + FM)$. The complexity of a two-block BCD algorithm has a complexity of $\mathcal{O}(\frac{1}{\sqrt{\epsilon_g}})$ [39]. Thus, the complexity of Alg. 1 is $\mathcal{O}((\frac{M^3K}{\log(\epsilon_\mu)} + K^2F^2 + FM)\frac{1}{\sqrt{\epsilon_g}})$. Similarly, we show the complexity to compute $\mathbf{W}^{(\eta)}$ to be $\mathcal{O}(MNK)$ while the RCG algorithm for update $\mathbf{V}^{(\eta)}$ is $\mathcal{O}(\epsilon_v^{-2})$, where ϵ_v^{-2} is the target accuracy of RCG. Since our beam decomposition leverages two-block BCD, its complexity is $\mathcal{O}((MNK + \epsilon_v^{-2})\frac{1}{\sqrt{\epsilon_q}})$. Hence, the total complexity of Alg. 2 is $\mathcal{O}((\frac{M^3K}{\log(\epsilon_\mu)} + K^2F^2 + FM)\frac{1}{\sqrt{\epsilon_g}} + (MNK + \epsilon_v^{-2})\frac{1}{\sqrt{\epsilon_q}})$.

> *We hope that the revised and highlighted discussions on the computational complexity clarify the proposed framework to the Reviewer's satisfaction.*

Reviewer Point 1.8 — *Please explain whether the proposed algorithm gives the local or global optimum, even though there is a discussion on no guarantee of global optimality.*

Authors' Response: We would like to thank Reviewer 1 for this interesting comment.

As pointed out in the previous manuscript, the RIS-assisted hybrid beamforming problem for GM rate optimization is a mixed-integer nonlinear programming (MINLP) problem with a highly nonconvex objective function. Consequently, the original joint hybrid beamforming and RIS design problem does not guarantee the existence of an **unique** global optimal solution. Moreover, the proposed algorithm approximate the optimal solution as a sequence of BCD technique solution while treat the integer variables as continuous counterpart. It can be shown that the solution obtained by the BCD technique always converges to a local optimum. However, no guarantee of global optimality can be provided as the quality of solution particularly depends on the initial value of variables. Hence our proposed algorithm only guarantee to obtain a local optimum.

We have added the following discussion in the revised manuscript:

> *Remark on the local optimum of proposed method:* We note that the solution obtained by the BCD technique always converges to a local optimum as the sequence created by iteratively optimizing the objective is a monotonic sequence. However, no guarantee of global optimality can be proved, as the original problem is strongly nonconvex and mixes integer variables. Hence, our proposed algorithm only guarantees obtaining a local optimum.

> *We hope that this clarification and the corresponding revision satisfactorily address the Reviewer's concern.*

Reviewer Point 1.9 — *In Fig. 4(b), how can the GM rate of Q = 4 perform better than that of 16 and infinity ? Perhaps you may need to average over more Monte Carlo simulations.*

Authors' Response: We would like to thank Reviewer 1 for this comment.

In the revised manuscript, we have performed the Monte Carlo simulations for Fig. 4 with the number of samples $S = 500$. Specifically, we randomly generated 500 samples of $\mathbf{G}$ and $\mathbf{h}_k$, and computed the optimal GM rate for each sample under our joint design hybrid beamforming and RIS algorithm for each bit-limited configuration. We then take the average GM rate of all samples to obtain the Monte Carlo simulation result.

As shown in Fig. 2, the simulation results exhibit a smoother performance curve with respect to $P_{\max}$ for the FC sub-array. This behavior contrasts with the fluctuations observed in Fig. 4(a) of the previous manuscript, which are attributed to the interference amplification characteristics of the beam decomposition and the quantization effects caused by the limited

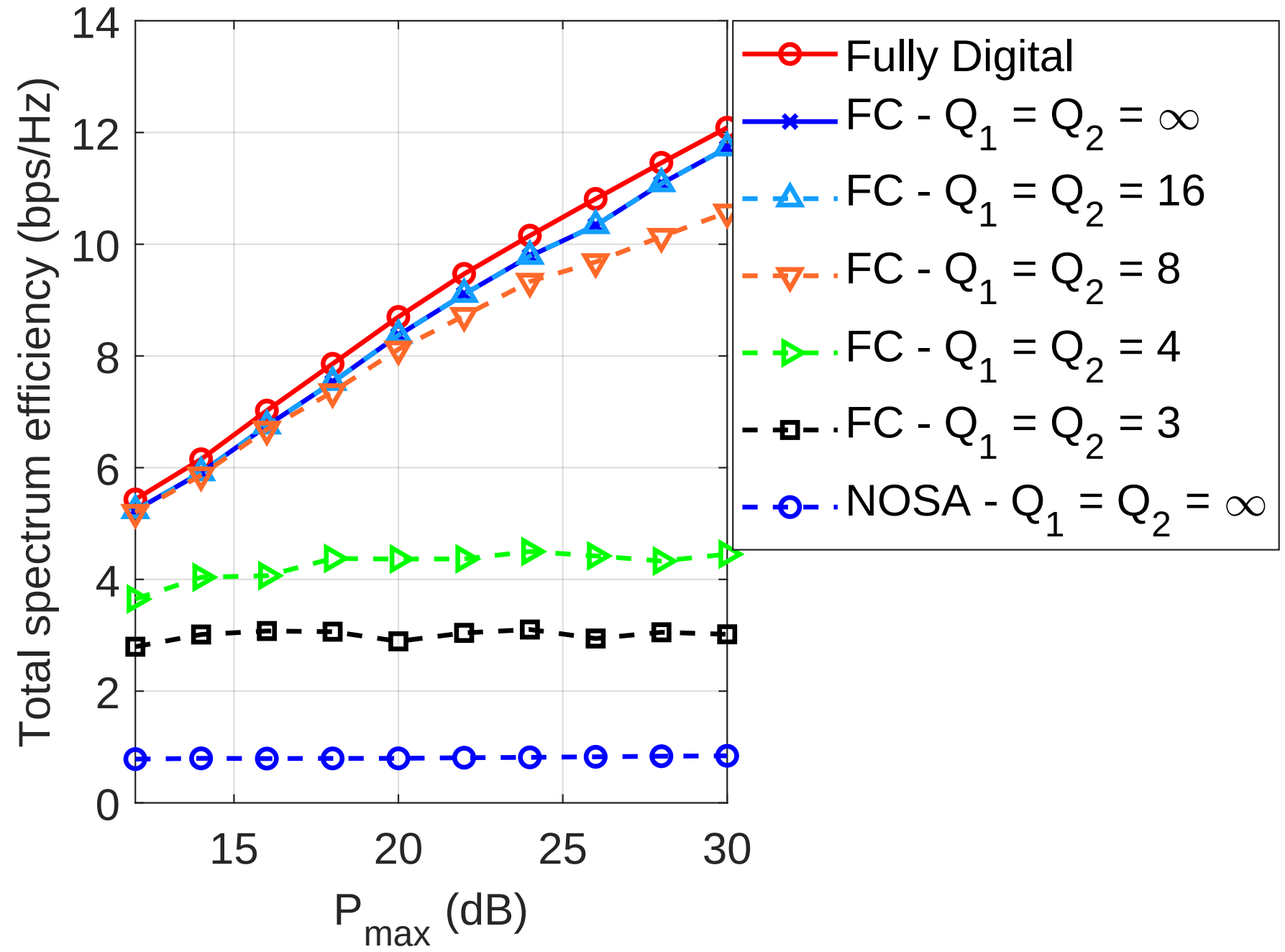

Figure 2: GMSE on different levels of $P_{\max}$ of FC beamforming.

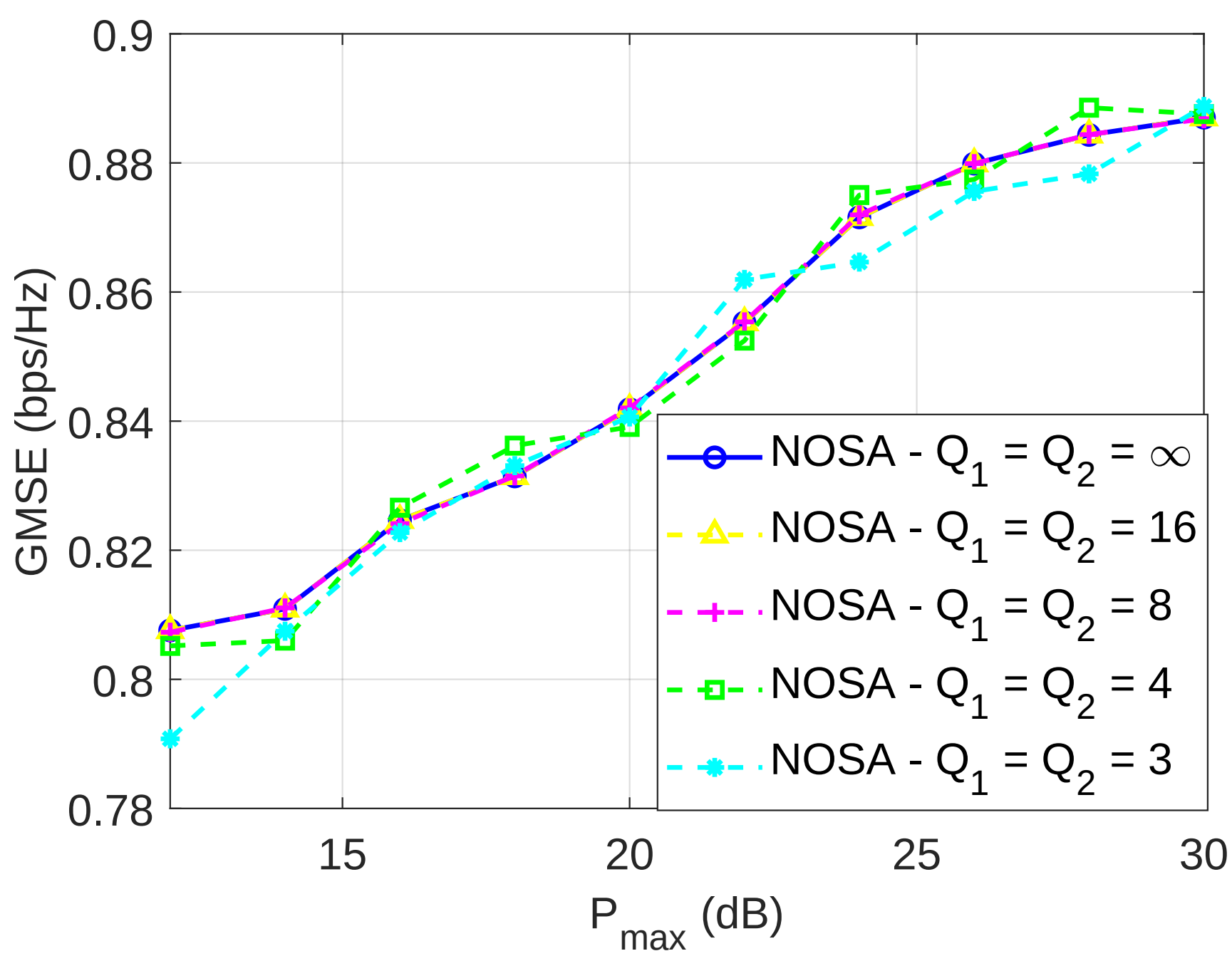

Figure 3: GMSE on different levels of $P_{\max}$ of NOSA beamforming.

number of control bits when operating around a suboptimal solution. This phenomenon suggests that our proposed algorithm can leverage the degree of freedom of FC and improve the overall performance on average in proportional to $P_{\max}$ levels.

However, as observed in Fig. 3, a small level of quantization-induced fluctuation remains for $Q_1 = Q_2 = 3$ and $Q_1 = Q_2 = 4$ in the NOSA configuration. This behavior can be attributed to the reduced degrees of freedom of the NOSA architecture compared to the FC case, which leads to increased inter-user interference in the desired signals. Consequently, the suboptimal solution obtained under the NOSA configuration is more pronounced than that of the FC configuration. As a result, when the system is controlled using a limited number of quantization bits, the GM rate can still be observed to slightly outperform the cases with 16 and infinite-resolution control, as the finite-bit quantization may partially suppress the interference introduced by the NOSA beam decomposition.

We have included the Monte Carlo simulation results in the revised manuscript along with the following discussion:

> Fig. 4 illustrates the overall performance across beamforming configurations, which we assume $Q_1 = Q_2 = Q$. Specifically, Fig. 4 demonstrates that the FD scheme yields the highest performance gains. This result is expected, as the FD configuration is equivalent to dedicating one RF chain to each BS antenna, thereby maximizing the system's capacity. Furthermore, using the FC sub-array architecture results in a better overall spectrum efficiency than the NOSA approach, even when the number of controller bits is limited. This is because the FC architecture's connection matrix has a higher density of non-zero elements. This inherent sparsity reduction grants the optimization process more degrees of freedom to precisely map the desired FD beam into its constituent analog and digital components, leading to a more optimal beamforming solution. This scenario suggests that 8 bits of quantization is sufficient to achieve a favorable trade-off between architecture complexity and performance for FC. Conversely, in the NOSA architecture, employing a small number of RF chains $N << M$ necessitates that each RF chain controls the phase of D antennas simultaneously. This coupling severely hampers the achievable beam decomposition gain, leading to a substantial performance loss. Specifically, this behavior can be attributed to the significantly lower density of non-zero elements of the NOSA architecture compared to the FC case, which leads to increased inter-user interference in the desired signals. Nevertheless, we observe that the system's performance saturates as the number of controller bits becomes sufficiently large, effectively matching the ideal continuous phase shift case $Q_1 = Q_2 = \infty$. This practical finding suggests that 3 bits of quantization for the discrete phase shifts at both the BS and RIS offer an adequate trade-off between implementation complexity and performance for NOSA.

> *We hope that this clarification and the corresponding revision satisfactorily address the Reviewer's concern.*

Reviewer Point 1.10 — *The CSI error model and Fig. 7 are unclear. You define NCPE in (34) but the x axis uses NMSE in dB and the caption says imperfect channel estimation, which is confusing.*

Authors' Response: We would like to thank Reviewer 1 for this comment.

We have changed the NMSE in Fig. 7 of previous manuscript to NCPE and changed the caption to "GM Rate Performance under imperfect channel estimation condition".

> *We hope that this clarification and the corresponding revision satisfactorily address the Reviewer's concern.*

Again, we would like to express our sincere appreciation to Reviewer 1 for the valuable time spent reviewing our work. We hope that Reviewer 1 will find our improvements satisfactory and that the revised version of our work is acceptable for publication in the PloS One.

Reviewer 2

General comments: *This manuscript studies an RIS-aided mmWave hybrid beamforming system that adopts an overlapped subarray architecture at the base station to improve spatial flexibility and hardware efficiency. The authors formulate a geometric mean rate maximization problem and develop a two-step BCD framework to optimize a fully digital equivalent precoder together with the RIS reflection phases. A Riemannian manifold-based factorization is applied to decompose the fully digital solution into realizable analog and digital parts under OSA constraints. The proposed initialization and optimization strategy improves convergence and achieves an 18.18 percent objective gain over previous methods. Simulation results show that 3-bit phase control is sufficient to approach continuous phase performance and that OSA can approach the performance of fully connected structures. However, several issues still need to be resolved in the following:*

Reviewer Point 2.1 — *The introduction explains the advantages of RIS and OSA architectures, but the manuscript does not clearly identify the specific fairness or reliability problem that geometric mean maximization uniquely addresses. Since GM rate optimization is the key point to your formulation, this manuscript needs a more concrete description of when and why the GM rate provides a necessary improvement over conventional sum rate objectives.*

Please clarify the fairness-related performance gap observed in existing RIS hybrid beamforming methods and provide a concrete scenario, such as blockage-prone or cell-edge users, where GM rate offers distinct advantages.

Authors' Response: We thank Reviewer 2 for this important comment regarding the motivation and necessity of the geometric mean (GM) rate objective.

We agree that the original version of the Introduction did not sufficiently articulate the *specific fairness and reliability problem* that GM-rate maximization is designed to address. In response, we have revised the Introduction to explicitly clarify (i) the fairness-related performance gap in existing RIS-assisted hybrid beamforming methods, (ii) the operating conditions under which this gap arises, and (iii) why GM-rate provides a necessary improvement over conventional sum-rate objectives.

(i) Fairness-related performance gap. The revised Introduction now explicitly states that, in RIS-assisted mmWave systems, user-rate imbalance is often *structural rather than incidental*. Due to severe blockage sensitivity, geometry-dependent path loss, and the passive nature of RIS reflections, users with unfavorable propagation conditions may experience near-zero instantaneous rates. However, conventional sum-rate maximization tolerates such rate collapse as long as the aggregate throughput is increased, leading to unreliable service for vulnerable users.

To make this gap explicit, we have added the following discussion (highlighted in blue in the revised Introduction):

> Recent path-loss measurements and channel modeling studies reveal that RIS-assisted mmWave links exhibit pronounced geometry-dependent attenuation and user-specific disparities, particularly in indoor and outdoor deployments [16,17]. Furthermore, statistical characterizations of RIS-assisted channels demonstrate non-trivial SNR distributions across near- and far-field regimes, which further intensify rate imbalance among users [18]. These model- and measurement-driven observations indicate that channel disparity in RIS-assisted mmWave systems is often structural rather than incidental, making fairness and service reliability first-order design challenges rather than secondary performance metrics.

(ii) When this gap becomes critical. The revised Introduction further clarifies that this fairness gap becomes particularly critical in *blockage-prone and quasi-static RIS-assisted mmWave scenarios*, where scheduling diversity is limited and fairness cannot be achieved through long-term averaging. In such settings, sum-rate-based designs may repeatedly favor users with strong composite channels, effectively starving blocked or cell-edge users.

This limitation is explicitly highlighted in the following added passage:

> Although these throughput-centric approaches improve aggregate spectral efficiency, they systematically favor users with strong composite channels and may severely degrade the service quality of cell-edge or blocked users. This limitation is particularly pronounced in RIS-assisted mmWave systems, where blockage and path-loss disparities are inherently amplified by passive reflection mechanisms.

(iii) Why GM-rate provides a necessary improvement. Finally, the revised Introduction clarifies why GM-rate is not merely an alternative fairness metric, but a *necessary design choice* under the above conditions. Unlike sum-rate objectives, GM-rate explicitly penalizes vanishing user rates and enforces balanced performance within each transmission interval, thereby preventing service outage for blocked or cell-edge users.

This role of GM-rate is now explicitly articulated as follows:

> Geometric mean (GM) rate maximization provides a principled mechanism to address this fairness and reliability gap by explicitly balancing individual user rates [28]. Unlike sum-rate objectives, which are insensitive to individual rate collapse, GM-rate penalizes vanishing user rates and enforces balanced performance across all users within a single transmission interval. This property is particularly important in quasi-static RIS-assisted mmWave systems, where limited scheduling diversity and reliance on reflected paths prevent fairness from being achieved through long-term averaging. Under OSA-based hybrid beamforming, GM-rate naturally aligns with reduced spatial degrees of freedom by prioritizing robustness and service continuity over peak throughput.

These revisions clarify that GM-rate optimization is introduced to address a concrete reliability and fairness gap in RIS-assisted mmWave hybrid beamforming, particularly in blockage-prone or cell-edge scenarios where sum-rate-based designs fail to guarantee service continuity.

> *We hope that these clarifications and the revised Introduction satisfactorily address the Reviewer's concern.*

Reviewer Point 2.2 — *The contribution list is long but does not clearly emphasize what is fundamentally new versus what is incremental. Some contributions are descriptive rather than conceptual.*

(1) Please refine the contribution list so that each point states a unique technical advancement rather than a procedural step.

(2) Please explicitly identify what part of the algorithm or framework is new relative to the conference version referenced in the manuscript.

Authors' Response: We thank Reviewer 2 for this comment. We agree that the original contribution list did not sufficiently separate what is fundamentally new from incremental details.

In the revised manuscript, we streamlined the contribution list and rewrote each item to state a distinct technical advancement (rather than a sequence of steps). The revised contribution list is *highlighted in blue* in the Introduction and now reads:

> 1. We formulate a fairness-driven GM-rate maximization problem for RIS-assisted mmWave systems with OSA-based hybrid beamforming and discrete RIS phases, thereby explicitly addressing the reliability gap inherent in sum-rate-centric designs.
> 2. We propose an OSA-consistent Riemannian manifold factorization that maps the fully-digital GM-optimal precoder benchmark into a realizable hybrid analog–digital structure without resorting to matrix lifting or semidefinite relaxation.
> 3. We develop an interference-aware warm-start strategy that stabilizes the power-constrained precoder update and accelerates convergence by up to 18.18% compared with random initialization.

We also revised the **Novelty Statement** to explicitly separate the journal contributions from the prior conference paper. The following text has been added and *highlighted in blue*:

> This journal article substantially extends the authors' preliminary conference paper presented at ATC 2025 [30] through several conceptual and methodological advancements rather than incremental refinements. Specifically, the journal version introduces: (i) a refined fairness-oriented optimization formulation that explicitly targets user-rate imbalance instead of aggregate throughput maximization, which was not considered in the conference version; (ii) a novel OSA-consistent Riemannian manifold factorization mechanism that rigorously maps the fully-digital optimum into a realizable hybrid analog–digital structure with discrete phase constraints; and (iii) an explicit theoretical and practical positioning with respect to emerging AI- and DRL-based RIS optimization approaches.

> We hope these revisions make the core contributions and the journal–conference differences clear.

Reviewer Point 2.3 — *The Introduction section covers conventional optimization approaches but does not discuss AI-based precoding or reinforcement learning-based RIS control, which have become major competing directions in this field. Avoiding these methods creates a gap in the contextualization of your contribution, because AI techniques are often applied to the same type of non-convex joint beamforming problems.*

(1) Please add a subsection in the introduction or related work that reviews AI-based hybrid beamforming and RIS optimization frameworks, including deep reinforcement learning approaches. This discussion should highlight the advantages and disadvantages (or limitations) of AI methods relative to your fully model-based optimization framework.

(2) Please include a comparison table summarizing classical optimization, your proposed method, and AI-based methods, highlighting differences in complexity, interpretability, data requirements, and scalability.

(3) Please cite and compare with the following work in your Introduction and Performance Evaluation (experiments):

P. -H. Chou, B. -R. Zheng, W. -J. Huang, W. Saad, Y. Tsao, and R. Y. Chang, "Deep Reinforcement Learning-Based Precoding for Multi-RIS-Aided Multiuser Downlink Systems With Practical Phase Shift," in IEEE Wireless Communications Letters, vol. 14, no. 1, pp. 23-27, Jan. 2025, doi: 10.1109/LWC.2024.3482720.

Since your system involves a single RIS, it is a simpler configuration than a multi-RIS system in the above work. Therefore, it should be included a comparison of performances, algorithmic complexity, fairness behavior, and practicality.

Authors' Response: We thank Reviewer 2 for raising this point. We agree that the original Introduction did not sufficiently contextualize our model-based framework against recent AI/DRL directions for RIS and hybrid beamforming.

(1) Added AI/DRL discussion and positioning: In the revised manuscript, we added a dedicated discussion in the Introduction to acknowledge AI/DRL-based RIS/precoding methods and to state, in concrete terms, why we adopt a model-based approach for fairness-critical designs. The added text is *highlighted in blue* and reads:

> In parallel, artificial intelligence (AI) and deep reinforcement learning (DRL) techniques have been actively explored for RIS and hybrid beamforming optimization [21,29]. Although these data-driven approaches exhibit strong adaptability in complex environments, they typically require large training datasets, lack interpretability, and do not provide explicit fairness guarantees. Model-based optimization frameworks therefore remain attractive for fairness-critical systems, as they offer transparent performance control, stable convergence behavior, and direct incorporation of hardware constraints. Accordingly, this work adopts a model-based design philosophy that complements, rather than replaces, AI-based RIS optimization.

(2) Added a comparison table: Following the Reviewer's suggestion, we have added:

> Table 1 highlights how the proposed framework uniquely addresses fairness and service reliability under practical hardware constraints when compared with classical and AI/DRL-based methods

The inserted table is:

Table 1: Comparative analysis of HBF optimization frameworks for RIS systems.

**Feature**	**Classical (SDR/WMMSE)**	**AI/DRL-based**	**Proposed work**
**Representative refs.**	[19,20,21]	[4,21,29]	This work
**Objective**	Aggregate sum-rate	Policy / reward-driven adaptation	**Fairness (GM-rate)**
**Complexity**	High (matrix lifting, iterative solvers)	Training-heavy (offline learning + online inference)	**Moderate (BCD + RCG)**
**Hardware realizability**	Idealized / relaxed constraints	Scenario-specific implementations	**OSA-consistent hybrid structure**
**Phase resolution**	Continuous (ideal assumption)	Discrete (heuristic or learned)	**Discrete (optimization-driven)**
**Mechanism**	Analytical optimization	Black-box policy learning	**Riemannian manifold optimization**
**Data dependency**	None	Massive datasets required	**CSI-only (model-based)**
**Service reliability**	Implicit (no rate-collapse control)	Not guaranteed	**Explicit (instantaneous GM-rate)**

(3) Added citation and clarified comparison to Chou et al. *(2025):* We have explicitly cited the suggested work [15] in the Introduction. We also added a brief technical comparison in the revised manuscript to clarify that [15] considers a multi-RIS downlink and targets DRL-based precoding under practical phase shifts, whereas our work focuses on a single-RIS setting but addresses a different system-level gap: fairness/reliability under blockage via GM-rate, together with an OSA-consistent hybrid realization under discrete phase constraints. This comparison text is also highlighted in blue in the revised manuscript.

> We hope these additions close the contextual gap noted by the Reviewer and clearly position our model-based, fairness-oriented framework relative to AI/DRL-based alternatives.

Reviewer Point 2.4 — *The two-step BCD procedure, the RIS phase update, and the Riemannian manifold factorization are mathematically correct but difficult for readers to follow on first reading. Several steps involve complex transformations that would benefit from intuition and a structural overview.*

(1) Please provide a high-level flow diagram summarizing the full optimization pipeline, showing the interaction between Q updates, RIS phase updates, and the hybrid decomposition stage.

(2) Please add brief intuitive explanations after major derivation steps to help us understand why each approximation or update rule is mathematically and conceptually reasonable.

Authors' Response: We would like to thank Reviewer 2 for this comment.

We have included a high-level flow diagram to capture the essence of our proposed algorithm in the revised manuscipt:

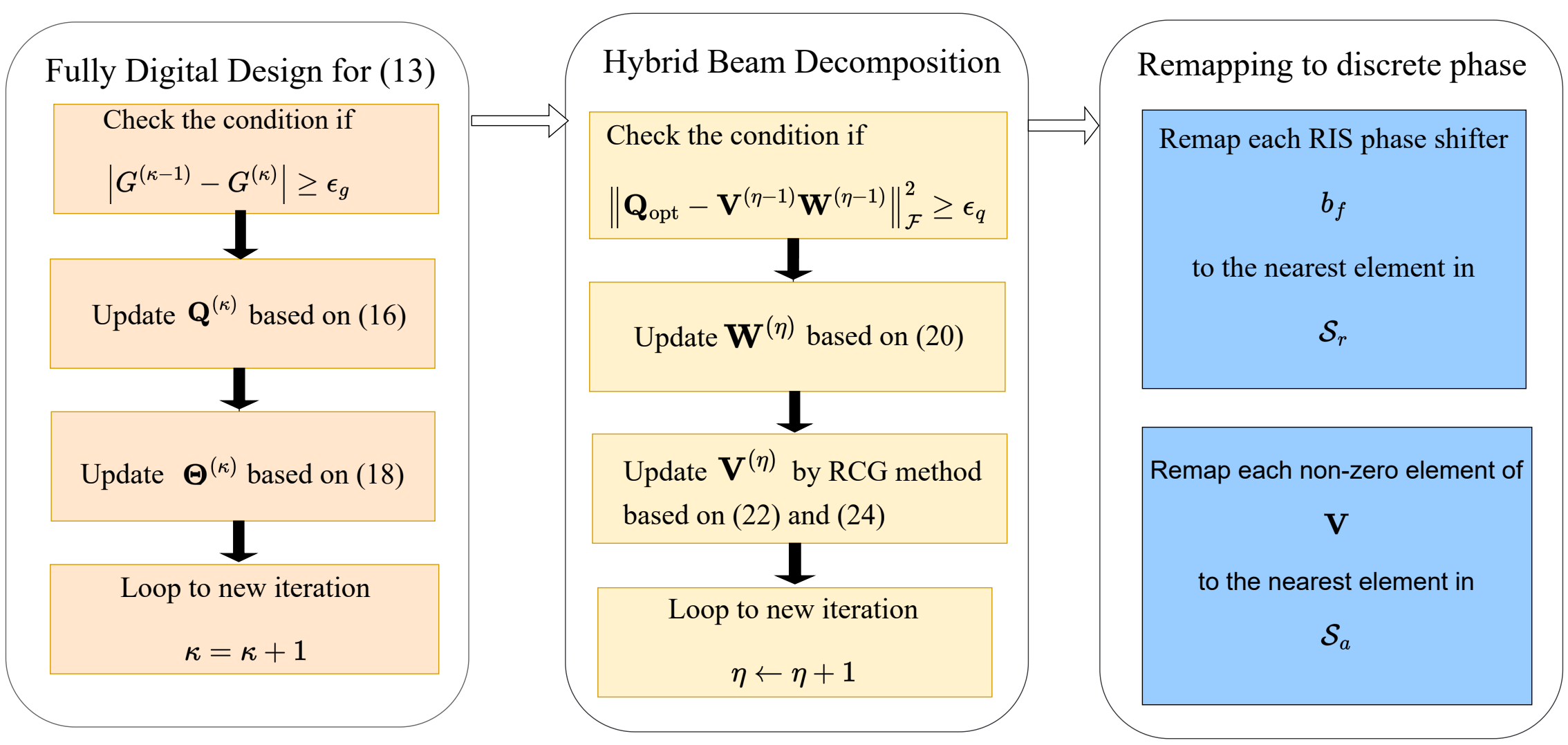

Figure 4: Diagram for proposed algorithm of problem (12)

We have added the following discussion in the revised manuscript:

> Toward this end, the procedure for solving problem (12) is summarized in Alg. 2 and the summarizing flow-diagram is given in Fig. 2. From the flow diagram, the overall optimization can be interpreted as an approximate decomposition of a highly coupled non-convex problem into a convex subproblem iteratively. In the fully digital design stage, the update for $(\mathbf{Q}^{(\kappa)}, \mathbf{\Theta}^{(\kappa)})$ is based on Lagrangian duality and optimal phase of affine sum. In the hybrid beam decomposition stage, we try to minimize the Euclidean distance between the optimal FD beam and the actual HBF beam, leading to the LS solution for $\mathbf{W}^{(\eta)}$ and RCG solution for $\mathbf{V}^{(\eta)}$. Together, these steps progressively enforce unit-modulus constraints while preserving the performance-oriented objective, which results in a tractable optimal solution.

We hope that the revised and highlighted discussions on the algorithm diagram clarify the proposed framework to the Reviewer's satisfaction.

Reviewer Point 2.5 — *Since the manuscript claims improved efficiency and faster convergence, such evidence should be quantified.*

(1) Please include a runtime or algorithmic complexity comparison to support the claims of computational efficiency.

(2) Please add two additional baselines, including WMMSE-based hybrid beamforming and a DDPG-based RIS control method, to contextualize the performance gains of your method.

Authors' Response: We would like to thank Reviewer 2 for this interesting comment.

(1) To analyze the computational complexity of the proposed joint hybrid beamforming and RIS phase shifts design, we note that the total runtime of the optimization procedure is given by sum of computational costs of the proposed Algorithm. 1 and Algorithm. 2. We will analyse the complexity of each algorithm as follows:

- **Algorithm.** 1: We consider the pseudo code of Algorithm 1. We recall that the update formulation of $\mathbf{Q}^{(\kappa)}$ is given by using the Lagrangian multiplier method as follows:

$$\mathbf{q}_k^{(\kappa+1)} = \begin{cases} \left(\Psi^{(\kappa)}\right)^{-1} \rho_k^{(\kappa)} \mathbf{y}^{(\kappa)}, & \text{if } \left\|\mathbf{Q}^{(\kappa+1)}\right\|^2 \leq P \\ \left(\Psi^{(\kappa)} + \mu I_M\right)^{-1} \rho_k^{(\kappa)} \mathbf{y}^{(\kappa)}, & \text{otherwise} \end{cases} \tag{13}$$

with $\mu > 0$ is a constant selected to satisfy the following equality by the bisection method:

$$\sum_{k=1}^{K} \left\| \left(\Psi^{(\kappa)} + \mu I_M\right)^{-1} \rho_k^{(\kappa)} b_k^{(\kappa)} \right\|^2 = P. \tag{14}$$

We note that for any dense square matrix $A \in \mathbb{C}^{n\times n}$ the computation complexity to calculate its inverse is $\mathcal{O}(n^3)$ [7]. We also observe that the matrix product between two matrices $A \in \mathbb{C}^{n\times m}$ and $B \in \mathbb{C}^{m\times p}$ is $\mathcal{O}(mnp)$. Thus, the complexity to compute $\mathbf{Q}^{(\kappa+1)}$ is $\mathcal{O}(M^3K)$ for a fixed value of μ. However, to select the constant μ that satisfies the given condition, we need an additional iteration complexity of $\mathcal{O}(M^3K/\log(\epsilon_\mu))$, where ϵ_μ is the target accuracy of the bisection method, since this method is $\mathcal{O}(\frac{1}{\log(\epsilon_\mu)})$ [8] and at each iteration we need to calculate the inverse and matrix multiplication of $\mathcal{O}(M^3K)$. Consider the complexity to update $\mathbf{\Theta}^{(\kappa)}$, we recall the equation:

$$\theta_f^{(\kappa+1)} = -\angle\Delta_f^{(\kappa)}, f \in \mathcal{F}. \tag{15}$$

By the definition of $\Delta_f^{(\kappa)}$, the computation complexity of is $\mathcal{O}(K^2F^2+FM)$. The complexity of a two-block BCD algorithm can be shown to have a complexity of $\mathcal{O}(\frac{1}{\sqrt{\epsilon_g}})$ [9]. Thus, the complexity of Algorithm. 1 is $\mathcal{O}((\frac{M^3K}{\log(\epsilon_\mu)} + K^2F^2 + FM)\frac{1}{\sqrt{\epsilon_g}})$.

- **Algorithm.** 2: We then consider the pseudo code of Algorithm 2. Similar to the analysis for Algorithm. 1, we show the complexity to compute $\mathbf{W}^{(\eta)}$ to be $\mathcal{O}(MNK)$ while the RCG algorithm for update $\mathbf{V}^{(\eta)}$ is $\mathcal{O}(\epsilon_v^{-2})$ where ϵ_v^{-2} is the target accuracy of RCG. Since the matrix decomposition procedure leverage two-block BCD, its complexity is $\mathcal{O}((MNK+\epsilon_v^{-2})\frac{1}{\sqrt{\epsilon_q}})$. Hence, the total complexity of Algorithm. 2 is $\mathcal{O}((\frac{M^3K}{\log(\epsilon_\mu)}+K^2F^2+FM)\frac{1}{\sqrt{\epsilon_g}}+(MNK+\epsilon_v^{-2})\frac{1}{\sqrt{\epsilon_q}})$.

We have added the following discussion in the revised manuscript:

> *Complexity Analysis of Alg. 2: To analyze the computational complexity of the proposed joint hybrid beamforming and RIS phase shifts design, it is essential to compute the complexity of Alg. 1 along with the complexity of RCG algorithm. We note that for any dense square matrix $A \in \mathbb{C}^{n\times n}$ the computation complexity to calculate its inverse is $\mathcal{O}(n^3)$ [37]. We also observe that the matrix product between two matrices $A \in \mathbb{C}^{n\times m}$ and $B \in \mathbb{C}^{m\times p}$ is $\mathcal{O}(mnp)$. Thus, the complexity of (16) is $\mathcal{O}(M^3K)$ for a fixed value of μ. However, to select the constant μ, we need an additional iteration complexity of $\mathcal{O}(M^3K/\log(\epsilon_\mu))$, where ϵ_μ is the target accuracy of the bisection method, since this method is $\mathcal{O}(\frac{1}{\log(\epsilon_\mu)})$ [38] and at each iteration we need to calculate the inverse and matrix multiplication of $\mathcal{O}(M^3K)$. Next, we consider the complexity of updating $\mathbf{\Theta}^{(\kappa)}$. We recall the definition of $\Delta_f^{(\kappa)}$ as (18), which is $\mathcal{O}(K^2F^2+FM)$. The complexity of a two-block BCD algorithm has a complexity of $\mathcal{O}(\frac{1}{\sqrt{\epsilon_g}})$ [39]. Thus, the complexity of Alg. 1 is $\mathcal{O}((\frac{M^3K}{\log(\epsilon_\mu)}+K^2F^2+FM)\frac{1}{\sqrt{\epsilon_g}})$. Similarly, we show the complexity to compute $\mathbf{W}^{(\eta)}$ to be $\mathcal{O}(MNK)$ while the RCG algorithm for update $\mathbf{V}^{(\eta)}$ is $\mathcal{O}(\epsilon_v^{-2})$, where ϵ_v^{-2} is the target accuracy of RCG. Since our beam decomposition leverages two-block BCD, its complexity is $\mathcal{O}((MNK+\epsilon_v^{-2})\frac{1}{\sqrt{\epsilon_q}})$. Hence, the total complexity of Alg. 2 is $\mathcal{O}((\frac{M^3K}{\log(\epsilon_\mu)}+K^2F^2+FM)\frac{1}{\sqrt{\epsilon_g}}+(MNK+\epsilon_v^{-2})\frac{1}{\sqrt{\epsilon_q}})$.*

(2) In the context of this paper, we consider the setting of RIS-aided hybrid beamforming with a general OSA architecture. There are several papers in the literature that leverage baselines mentioned by the Reviewer, including WMMSE-based hybrid beamforming method [16, 17, 18] and DDPG-based RIS control method [19]. However, to the best of our knowledge, most of the existing works that consider DDPG-based RIS control with WMMSE-based HBF are only suitable for the HBF fully connected architecture, while the general OSA architecture has not yet been considered. As such, these existing baselines serve a different system model and are not appropriate to objectively compare the performance gains of our method.

> *We hope that these discussions and the corresponding revision satisfactorily address the Reviewer's concern.*

Reviewer Point 2.6 — *The manuscript motivates OSA hardware feasibility and low phase resolution, but the practical context of these choices is not illustrated. Since RIS and hybrid*

architectures face real-world constraints, the relevance of the proposed method would be clearer with concrete examples.

(1) Please describe one or two realistic deployment scenarios where OSA-based RIS hybrid beamforming is beneficial, such as indoor hotspot expansion, factory automation, or blockage mitigation.

(2) Please briefly discuss control overhead, calibration, or signaling requirements for RIS phase updates and OSA analog precoding.

Authors' Response: We would like to thank Reviewer 2 for this interesting comment.

(1) OSA-based RIS hybrid beamforming is particularly attractive in realistic mmWave deployment scenarios where hardware cost, energy efficiency, and robustness to blockage are primary concerns. One representative example is indoor hotspot expansion, such as shopping malls, airports, and large office buildings, where mmWave links suffer from severe penetration loss and frequent human blockage. In such environments, OSA hybrid architectures significantly reduce the number of phase shifters and RF interconnections compared to fully-connected structures, thereby lowering hardware complexity and power consumption while preserving sufficient data rate [10, 11]. When combined with RIS panels deployed on walls or ceilings, controllable reflected paths can be created to enhance coverage in non-line-of-sight (NLoS) regions, which aligns with standardized indoor hotspot channel models defined in 3GPP TR 38.901 standard. Another practical deployment scheme is for industrial automation and smart factories, where metallic structures, moving robots, and machinery frequently block line-of-sight links. In such settings, reliability and low latency are more critical than extreme spectral efficiency. As such, OSA hybrid beamforming provides a hardware-efficient solution with reduced calibration complexity, while RIS-assisted propagation control can dynamically redirect signals around obstacles to maintain link robustness. Thus, this deployment perspective is consistent with IMT-2020 requirements and industrial 5G use case scenarios.

(2) From a practical implementation perspective, the control overhead and calibration requirements of the proposed OSA-based RIS hybrid architecture remain manageable. For RIS phase updates, configuration signaling is typically performed over a low-rate control link and updated at the channel coherence time scale. Since practical RIS implementations employ discrete phase shifts with limited resolution, the control payload scales linearly with the number of elements and phase quantization bits, which helps constrain signaling overhead [12, 13]. Moreover, element grouping or codebook-based reflection design can further reduce configuration complexity.

For the OSA analog precoder, the subarray structure significantly reduces the number of phase shifters and RF interconnections compared to fully-connected architectures, thereby simplifying RF calibration and hardware synchronization [14, 11]. Because each RF chain only drives a subset of antennas, the dimensionality of analog beam training and feedback is also reduced, leading to lower beam-search overhead. Consequently, the combined OSA-RIS architecture achieves a favorable trade-off between performance and implementation complexity, making it suitable for practical mmWave deployments.

We have added the following discussion in the Introduction of the revised manuscript:

Meanwhile, OSA-based RIS hybrid beamforming is particularly suitable for practical mmWave deployments where blockage is a major concern, such as indoor hotspots in shopping malls, airports, and large office buildings. In these scenarios, severe penetration loss and frequent human blockage degrade mmWave links. Compared to fully connected architectures, the OSA structure reduces the number of phase shifters and RF interconnections, thereby lowering hardware complexity and power consumption while maintaining satisfactory data rates. Moreover, the control and calibration overhead remains manageable, as RIS phase updates are performed over a low-rate control link at the channel coherence time scale, and with discrete phase shifts of limited resolution, the signaling payload scales linearly with the number of elements and quantization bits.

We hope that these discussions and the corresponding revision satisfactorily address the Reviewer's concern.

Reviewer Point 2.7 — *Several notation-related issues in the manuscript affect readability.*

(1) Please prepare a notation table placed either at the end of the Introduction or at the beginning of the System Model.

The table should include:

(a) All variables, vectors, matrices, and sets

(b) A concise description of their roles

(c) Dimensions where appropriate

(d) Constraints (unit modulus, discrete phase set, manifold space, etc.)

(e) Iteration indices with a clear explanation of which algorithmic stage they belong to

(2) Please unify the superscript and subscript conventions so that iteration indices, user indices, antenna indices, and RIS indices follow a consistent ordering and formatting.

(3) Please ensure each symbol is explicitly defined at its first occurrence and that symbols reused for different purposes (such as μ variations) are renamed or clarified to avoid ambiguity.

Authors' Response: We thank the Reviewer for the helpful comments regarding notation clarity and consistency.

(1) We have added a dedicated *notation table* at the beginning of the System Model section to summarize all variables, vectors, matrices, and sets, together with their dimensions, roles, and associated constraints, as well as iteration indices and their corresponding algorithmic stages.

The table 2 summarizes all key symbols, including variables, vectors, matrices, and sets, together with their dimensions and concise descriptions.

(2) We have unified the superscript and subscript conventions throughout the manuscript so that user indices (k), RF-chain indices (n), RIS element indices (f), and iteration indices are consistently ordered and formatted across all algorithmic stages.

Table 2: List of symbols and notations

Symbol	Dimension / Set	Description
$k \in \mathcal{K}$	$\{1, \ldots, K\}$	User index and user set
$n \in \mathcal{N}$	$\{1, \ldots, N\}$	RF-chain index
$f \in \mathcal{F}$	$\{1, \ldots, F\}$	RIS element index
κ	$\mathbb{Z}_+$	Iteration index of BCD optimization
η	$\mathbb{Z}_+$	Iteration index of hybrid beamforming factorization
M	$\mathbb{Z}_+$	Number of BS antennas
N	$\mathbb{Z}_+$	Number of RF chains
F	$\mathbb{Z}_+$	Number of RIS reflecting elements
P_0	$\mathbb{R}_+$	Maximum transmit power at the BS
σ_k^2	$\mathbb{R}_+$	Noise variance at user k
$\mathbf{w}_k$	$\mathbb{C}^{N \times 1}$	Digital beamforming vector for user k
$\mathbf{W}$	$\mathbb{C}^{N \times K}$	Digital beamforming matrix
$\mathbf{V}$	$\mathbb{C}^{M \times N}$	Analog beamforming matrix with OSA structure
$\boldsymbol{\Theta}$	$\mathbb{C}^{F \times F}$	RIS reflection coefficient matrix
b_f	$\mathbb{C}$	Reflection coefficient of RIS element f
$\mathbf{G}$	$\mathbb{C}^{F \times M}$	BS–RIS channel matrix
$\mathbf{h}_k$	$\mathbb{C}^{F \times 1}$	RIS–user k channel vector
R_k	$\mathbb{R}_+$	Achievable rate of user k
$\mathbf{Q}$	$\mathbb{C}^{M \times K}$	Fully-digital equivalent precoder
$G(\cdot)$	$\mathbb{R}_+$	Geometric mean (GM) rate objective
$\rho_k^{(\kappa)}$	$\mathbb{R}_+$	GM linearization weight for user k at iteration κ
μ	$\mathbb{R}_+$	Lagrange multiplier associated with the BS transmit power constraint
$\mu_k^{(\kappa)}$	$\mathbb{R}_+$	SINR-related auxiliary variable for user k at iteration κ
$\mathscr{V}$	Manifold	Feasible manifold of unit-modulus analog beamforming coefficients

(3) We have ensured that every symbol is explicitly defined at its first occurrence, and symbols reused in different contexts have been clarified to avoid ambiguity. In particular, the previously overloaded symbol μ has been explicitly disambiguated. Specifically, μ denotes the Lagrange multiplier associated with the BS transmit power constraint, whereas $\mu_k^{(\kappa)}$ represents the SINR-related auxiliary variable for user k at iteration κ. Both definitions are explicitly distinguished and summarized in the notation table 2.

We believe that these revisions resolve the notation-related issues and significantly improve the readability of the manuscript.

Reviewer Point 2.8 — *Comments on the Quality of English.*

(1) Sentences are often too long and contain multiple nested clauses, which makes several technical descriptions difficult to follow. Please break long sentences into shorter units and simplify clause structure to improve clarity, especially in Sections 2 and 3.

(2) Some symbols and technical terms appear before being clearly defined, and several pronouns ("this," "that," "these") refer to ambiguous antecedents. Please ensure that each variable is explicitly defined at first use and replace vague pronouns with specific referents to reduce ambiguity.

(3) Minor grammatical inconsistencies appear throughout the manuscript, including article usage, subject-verb agreement, and inconsistent compound noun formatting (e.g., "fully digital" vs "fully-digital," "phase shifter" vs "phase-shifter"). Please perform a careful language pass to correct these recurring issues and maintain consistent technical terminology.

Authors' Response: We thank the Reviewer for the detailed comments on the quality of English.

(1) The manuscript has been thoroughly revised to improve clarity and readability. The revised passages are explicitly **highlighted in blue** in the submitted manuscript to clearly indicate where sentence structures have been simplified and split for improved clarity.

(2) All symbols and technical terms are now explicitly defined at their first occurrence. In addition, ambiguous pronouns such as "this," "that," and "these" have been replaced with precise references to the corresponding variables, equations, or algorithmic steps to eliminate unclear antecedents.

(3) A careful language pass has been conducted across the entire manuscript to correct minor grammatical inconsistencies, including article usage and subject–verb agreement, and to ensure consistent technical terminology and formatting (e.g., "fully-digital," "hybrid beamforming," and "phase-shifter").

We believe that these revisions substantially improve the clarity, precision, and overall readability of the manuscript.

Again, we would like to express our sincere appreciation to Reviewer 2 for the valuable time spent reviewing our work. We hope that Reviewer 2 will find our improvements satisfactory and that the revised version of our work is acceptable for publication in the PloS One.

Reviewer 3

General comments: *This paper investigated a RIS-aided hybrid beamforming system designed for scalable operation in the millimeter wave channel. The authors proposed a framework that jointly optimizes the linear transmit beamformers and the programmable reflecting coefficients of the RIS elements. Generally, the authors have done an interesting work, however, some parts need to be further improved. The Reviewer has the following concerns:*

Reviewer Point 3.1 — *This paper mentions using geometric mean to enhance user fairness but fails to deeply analyze specific failure cases of existing sum-rate maximization methods in dynamic environments, such as not detailing how geometric mean handles channel fluctuations or user mobility.*

Authors' Response: We would like to thank Reviewer 3 for this interesting comment.

In this paper, we consider the problem of jointly design hybrid beamforming and RIS phase shift under static channel condition. In particular, we consider the channel matrices $\mathbf{G}$ and $\mathbf{h}_k$ to be unchanged during the HBF design process. Thus, the consideration of geometric mean for channel fluctuations or user mobility falls outside the scope of this paper.

However, even with static channel condition, several papers in literature have addressed the imbalance of sum-rate maximization in ensuring QoS for users with poor channel condition. Specifically, the authors in [6] have shown the existence of zero-rate users, i.e. users with data rate equals to zero, when jointly design hybrid beamforming and RIS phase based on sum-rate maximization methods for the case of $K > M$. Furthermore, this paper demonstrates that the GM rate formulation ensures that no user experiences a zero rate under the same settings while maintains a considerable overall QoS. Hence, it can be seen that for the case of $K > M$, GM formulation can guarantee sufficient resource for users with inferior channel condition, while enhancing the overall rate of the network.

> *We hope that the discussion on drawback of existing sum-rate maximization methods and the corresponding revision satisfactorily address the Reviewer's concern.*

Reviewer Point 3.2 — *The adopted BCD algorithm and manifold optimization are essentially an extension of traditional optimization frameworks, and it does not explain why this framework has inherent advantages over traditional methods in convergence speed or resource efficiency.*

Authors' Response: We would like to thank Reviewer 3 for this interesting comment.

The proposed BCD framework combined with manifold optimization is not intended as a mere reformulation of traditional alternating optimization methods, but rather as a structure-exploiting strategy tailored to the specific constraints of the considered problem. First, the block coordinate descent (BCD) framework decomposes the high-dimensional joint optimization into smaller subproblems with closed-form or efficiently solvable updates, which

significantly reduces per-iteration computational burden compared to solving the full problem jointly. Second, the RIS phase design naturally involves unit-modulus constraints, which form a complex circle manifold. Manifold optimization explicitly exploits this geometric structure, enabling gradient updates that remain on the feasible set without requiring projection or penalty relaxation. Compared to conventional Euclidean optimization with projection steps, manifold-based updates typically exhibit improved numerical stability and avoid constraint violation accumulation. Therefore, while the overall framework is rooted in classical optimization principles, the adopted formulation leverages problem structure to improve computational efficiency and convergence behavior for RIS-assisted hybrid beamforming systems.

> *We hope that this discussions and the corresponding revision satisfactorily address the Reviewer's concern.*

Reviewer Point 3.3 — *The method design relies on ideal channel assumptions, such as perfect CSI or static interference, but does not account for practical estimation errors, feedback delays, or hardware nonlinearities.*

Authors' Response: We would like to thank Reviewer 3 for this interesting comment.

We appreciate this observation. While we agree that obtaining perfect Channel State Information (CSI) in real-world communication systems is challenging, we would like to clarify the main region behind our assumption, which is also widely adopted in the literature, as follows:

- **Establishing Performance Benchmarks:** Assuming perfect CSI allows researchers to derive theoretical upper bounds (e.g., channel capacity, spectral efficiency). These bounds serve as benchmarks for evaluating practical systems.
- **Guiding System and Algorithm Design:** Optimal performance analysis under perfect CSI provides valuable insights for designing modulation schemes, coding strategies, and beamforming algorithms that aim to approach ideal performance.
- **Isolating System Limitations:** By eliminating uncertainties in channel estimation, one can focus on other system constraints such as power budgets, hardware impairments, or algorithmic complexity.
- **Quantifying the Impact of Imperfect CSI:** Comparing ideal and practical performance reveals how much is lost due to channel estimation errors, helping motivate better estimation and feedback techniques.
- **Benchmarking for Learning-based Methods:** Perfect CSI scenarios are often used as training environments for machine learning models, enabling them to learn optimal strategies before generalizing to realistic cases.

- **Simplifying Theoretical Analysis:** Perfect CSI leads to tractable mathematical models, allowing derivation of closed-form expressions and analytical insights that inform system-level design decisions.

Upon these, we examine that CSI can be perfectly estimated or statistically acquired via standard pilot-based techniques [20] under condition of slowly varying channels. However, incorporating these factors in our work would require additional modeling layers, including delay-aware control mechanisms, and hardware impairment-aware beamforming design. Such extensions would significantly increase analytical and computational complexity and are therefore beyond the scope of the present work. Nevertheless, extending the proposed OSA-based RIS hybrid beamforming framework to robust optimization formulations under imperfect CSI and hardware nonidealities constitutes an important direction for future research. We will consider these practical aspects in subsequent studies to further enhance the applicability of the proposed method in realistic deployment scenarios.

We have included the following discussion in the revised manuscript:

> It is important to highlight that the assumption of CSI at the base station is widely used in HBF for mmWave communication studies [24,28,31], as it enables tractable optimization and serves as a benchmark for performance evaluation. In this work, CSI refers to either perfect or reliably estimated values obtained through standard pilot-based training methods [32], which are practical in slow-fading or quasi-static conditions. Although this assumption simplifies the analysis, it provides meaningful upper bounds for system design.

> *We hope that the revised and highlighted discussions on the perfect CSI assumption clarify the proposed framework to the Reviewer's satisfaction.*

Reviewer Point 3.4 — *It is suggested to introduce the following highly-impact recent works in RIS [R1]–[R4] related to highlight the state-of-art of this paper:*

R1 "Exploiting multi-layer stacking RIS-assisted receiver for HAP-SWIPT networks," IEEE Transactions on Wireless Communications, vol. 23, no. 10, pp. 12638–12657, Oct. 2024.

R2 "Self-powered adaptive reconfigurable intelligent surfaces for securing satellite-terrestrial integrated networks," China Communications, vol. 21, no. 9, pp. 276–291, Sep. 2024.

R3 "Refracting RIS aided hybrid satellite-terrestrial relay networks: Joint beamforming design and optimization," IEEE Transactions on Aerospace and Electronic Systems, vol. 58, no. 4, pp. 3717–3724, Aug. 2022.

R4 "Air-driven seamless and massive access in space-air-ground integrated networks," IEEE Wireless Communications, vol. 32, no. 3, pp. 72–79, Jun. 2025.

Authors' Response:

We sincerely thank the Reviewer for the helpful suggestion. Following this recommendation, we have revised the Introduction to incorporate the suggested recent RIS works [R1]–[R4] in order to better reflect the latest developments in advanced RIS architectures and space–air–ground integrated networks.

Specifically, we have added a new discussion paragraph in the Introduction (see the revised manuscript, highlighted in blue) that now cites the recommended works and positions them within the broader context of multi-layer RIS, self-powered RIS, refracting RIS, and RIS-enabled massive access. The added text reads:

> Reconfigurable intelligent surfaces (RISs) have emerged as a key enabler of this paradigm shift by transforming the wireless environment into a programmable entity [3]. Comprehensive surveys have established RIS as a fundamental building block of smart radio environments, capable of reshaping wireless propagation to support emerging 6G services [4, 5]. Early RIS research primarily focused on enhancing signal strength and spectral efficiency. More recent studies reposition RISs as multi-functional and reliability-critical platforms that integrate communication, sensing, and wireless power transfer [6, 7]. In parallel, advanced RIS architectures, including multi-layer, refracting, and self-powered designs, have been investigated to support satellite–terrestrial and space–air–ground integrated networks [8 – 10]. For instance, multi-layer stacking RIS receivers have been studied for high-altitude platform (HAP)-enabled simultaneous wireless information and power transfer (SWIPT) networks [11], while self-powered adaptive RIS designs have been considered for secure satellite-terrestrial integration [12]. Refracting RIS has also been investigated for hybrid satellite-terrestrial relay systems [13]. More recently, air-driven seamless and massive access has been discussed for space-air-ground integrated networks, where aerial platforms (e.g., unmanned aerial vehicle (UAV) and HAP tiers) play a coordinating role to enable wide-area continuity of access and large-scale user connectivity across heterogeneous layers [14]. These developments are consistent with broader 6G visions that emphasize scalable and intelligent connectivity across heterogeneous network layers [15].

> We believe that these revisions adequately address the Reviewer's comment and improve the completeness of the state-of-the-art discussion.

Reviewer Point 3.5 — *The scheme does not discuss computational complexity or real-time processing requirements, which may cause high latency on resource-constrained base stations.*

Authors' Response: We would like to thank Reviewer 3 for this comment.

To analyze the computational complexity of the proposed joint hybrid beamforming and RIS phase shifts design, we note that the total runtime of the optimization procedure is given by sum of computational costs of the proposed Algorithm. 1 and Algorithm. 2. We will analyse the complexity of each algorithm as follows:

- **Algorithm.** 1: We consider the pseudo code of Algorithm 1. We recall that the update formulation of $\mathbf{Q}^{(\kappa)}$ is given by using the Lagrangian multiplier method as follows:

$$\mathbf{q}_k^{(\kappa+1)} = \begin{cases} \left(\Psi^{(\kappa)}\right)^{-1} \rho_k^{(\kappa)} \mathbf{y}^{(\kappa)}, & \text{if } \left\|\mathbf{Q}^{(\kappa+1)}\right\|^2 \leq P \\ \left(\Psi^{(\kappa)} + \mu I_M\right)^{-1} \rho_k^{(\kappa)} \mathbf{y}^{(\kappa)}, & \text{otherwise} \end{cases} \tag{16}$$

with $\mu > 0$ is a constant selected to satisfy the following equality by the bisection method:

$$\sum_{k=1}^{K} \left\| \left(\Psi^{(\kappa)} + \mu I_M\right)^{-1} \rho_k^{(\kappa)} b_k^{(\kappa)} \right\|^2 = P. \tag{17}$$

We note that for any dense square matrix $A \in \mathbb{C}^{n\times n}$ the computation complexity to calculate its inverse is $\mathcal{O}(n^3)$ [7]. We also observe that the matrix product between two matrices $A \in \mathbb{C}^{n\times m}$ and $B \in \mathbb{C}^{m\times p}$ is $\mathcal{O}(mnp)$. Thus, the complexity to compute $\mathbf{Q}^{(\kappa+1)}$ is $\mathcal{O}(M^3K)$ for a fixed value of μ. However, to select the constant μ that satisfies the given condition, we need an additional iteration complexity of $\mathcal{O}(M^3K/\log(\epsilon_\mu))$, where ϵ_μ is the target accuracy of the bisection method, since this method is $\mathcal{O}(\frac{1}{\log(\epsilon_\mu)})$ [8] and at each iteration we need to calculate the inverse and matrix multiplication of $\mathcal{O}(M^3K)$. Consider the complexity to update $\mathbf{\Theta}^{(\kappa)}$, we recall the equation:

$$\theta_f^{(\kappa+1)} = -\angle\Delta_f^{(\kappa)}, f \in \mathcal{F}. \tag{18}$$

By the definition of $\Delta_f^{(\kappa)}$, the computation complexity is $\mathcal{O}(K^2F^2 + FM)$. The complexity of a two-block BCD algorithm can be shown to have a complexity of $\mathcal{O}(\frac{1}{\sqrt{\epsilon_g}})$ [9]. Thus, the complexity of Algorithm. 1 is $\mathcal{O}((\frac{M^3K}{\log(\epsilon_\mu)} + K^2F^2 + FM)\frac{1}{\sqrt{\epsilon_g}})$.

- **Algorithm.** 2: We then consider the pseudo code of Algorithm 2. Similar to the analysis for Algorithm. 1, we show the complexity to compute $\mathbf{W}^{(\eta)}$ to be $\mathcal{O}(MNK)$ while the RCG algorithm for update $\mathbf{V}^{(\eta)}$ is $\mathcal{O}(\epsilon_v^{-2})$ where ϵ_v^{-2} is the target accuracy of RCG. Since the matrix decomposition procedure leverage two-block BCD, its complexity is $\mathcal{O}((MNK + \epsilon_v^{-2})\frac{1}{\sqrt{\epsilon_q}})$. Hence, the total complexity of Algorithm. 2 is $\mathcal{O}((\frac{M^3K}{\log(\epsilon_\mu)} + K^2F^2 + FM)\frac{1}{\sqrt{\epsilon_g}} + (MNK + \epsilon_v^{-2})\frac{1}{\sqrt{\epsilon_q}})$.

We have added the following discussion in the revised manuscript:

Complexity Analysis of Alg. 2: To analyze the computational complexity of the proposed joint hybrid beamforming and RIS phase shifts design, it is essential to compute the complexity of Alg. 1 along with the complexity of RCG algorithm. We note that for any dense square matrix $A \in \mathbb{C}^{n\times n}$ the computation complexity to calculate its inverse is $\mathcal{O}(n^3)$ [37]. We also observe that the matrix product between two matrices $A \in \mathbb{C}^{n\times m}$ and $B \in \mathbb{C}^{m\times p}$

is $\mathcal{O}(mnp)$. Thus, the complexity of (16) is $\mathcal{O}(M^3K)$ for a fixed value of μ. However, to select the constant μ, we need an additional iteration complexity of $\mathcal{O}(M^3K/\log(\epsilon_\mu))$, where ϵ_μ is the target accuracy of the bisection method, since this method is $\mathcal{O}(\frac{1}{\log(\epsilon_\mu)})$ [38] and at each iteration we need to calculate the inverse and matrix multiplication of $\mathcal{O}(M^3K)$. Next, we consider the complexity of updating $\mathbf{\Theta}^{(\kappa)}$. We recall the definition of $\Delta_f^{(\kappa)}$ as (18), which is $\mathcal{O}(K^2F^2 + FM)$. The complexity of a two-block BCD algorithm has a complexity of $\mathcal{O}(\frac{1}{\sqrt{\epsilon_g}})$ [39]. Thus, the complexity of Alg. 1 is $\mathcal{O}((\frac{M^3K}{\log(\epsilon_\mu)} + K^2F^2 + FM)\frac{1}{\sqrt{\epsilon_g}})$. Similarly, we show the complexity to compute $\mathbf{W}^{(\eta)}$ to be $\mathcal{O}(MNK)$ while the RCG algorithm for update $\mathbf{V}^{(\eta)}$ is $\mathcal{O}(\epsilon_v^{-2})$, where ϵ_v^{-2} is the target accuracy of RCG. Since our beam decomposition leverages two-block BCD, its complexity is $\mathcal{O}((MNK + \epsilon_v^{-2})\frac{1}{\sqrt{\epsilon_q}})$. Hence, the total complexity of Alg. 2 is $\mathcal{O}((\frac{M^3K}{\log(\epsilon_\mu)} + K^2F^2 + FM)\frac{1}{\sqrt{\epsilon_g}} + (MNK + \epsilon_v^{-2})\frac{1}{\sqrt{\epsilon_q}})$.

We hope that the revised and highlighted discussions on the computational complexity clarify the proposed framework to the Reviewer's satisfaction.

Reviewer Point 3.6 — *The performance evaluation only focuses on geometric mean rate and convergence, neglecting key metrics like energy efficiency, complexity, or anti-interference resilience.*

We hope that these discussions and the corresponding revision satisfactorily address the Reviewer's concern.

Authors' Response: We would like to thank Reviewer 3 for this comment.

In the current manuscript, the performance evaluation focuses primarily on geometric mean rate and convergence behavior, as these metrics directly reflect the effectiveness and stability of the proposed optimization framework. However, we agree that additional system-level metrics such as energy efficiency, computational complexity, and robustness against interference are important for a more comprehensive performance assessment.

In particular, energy efficiency analysis would require incorporating detailed power consumption models for RF chains, phase shifters, and RIS control circuitry, while anti-interference resilience would necessitate extended simulations under imperfect CSI, dynamic blockage, or co-channel interference scenarios. These aspects introduce additional modeling dimensions beyond the current scope of this work. We will consider these important performance metrics in future research to further evaluate the practical deployment potential of the proposed OSA-based RIS hybrid beamforming framework.

Reviewer Point 3.7 — *The writing is somewhat abrupt in algorithm flow and symbol definitions, such as not clearly defining abbreviations or optimization variables.*

Authors' Response: We thank the Reviewer for the constructive comment. The manuscript has been substantially revised to improve the clarity of notation, abbreviation usage, and the overall algorithmic flow, particularly in the portions highlighted in blue.

First, we have added a dedicated table of abbreviations to ensure that all key terms (e.g., RIS, HBF, OSA) are clearly defined and consistently used throughout the manuscript. Second, the main optimization variables, including the digital beamforming matrix $\mathbf{W}$, the analog beamforming matrix $\mathbf{V}$, and the RIS response matrix $\mathbf{\Theta}$, are now explicitly introduced in the System Model and Problem Formulation sections together with their dimensions and roles. Third, we have streamlined the presentation of the proposed algorithm and added a comparative table against existing methods to improve readability and highlight the methodological distinctions.

The table 2 summarizes all key symbols, including variables, vectors, matrices, and sets, together with their dimensions and concise descriptions.

> *Overall, the manuscript has been carefully rewritten to ensure a smoother logical progression and improved technical clarity. We believe that these revisions adequately address the Reviewer's comment and improve the readability and structural coherence of the manuscript.*

References

[1] Tam HHM, Tuan HD, Ngo DT. Successive Convex Quadratic Programming for Quality-of-Service Management in Full-Duplex MU-MIMO Multicell Networks. IEEE Transactions on Communications. 2016;64(6):2340–2353. doi:10.1109/TCOMM.2016.2550440.

[2] Jiang J, Yuan Y, Zhen L. Multi-User Hybrid Precoding for Dynamic Subarrays in mmWave Massive MIMO Systems. IEEE Access. 2019;7:101718–101728. doi:10.1109/ACCESS.2019.2929927.

[3] Dong F, Liu F, Cui Y, Wang W, Han K, Wang Z. Sensing as a Service in 6G Perceptive Networks: A Unified Framework for ISAC Resource Allocation. IEEE Transactions on Wireless Communications. 2023;22(5):3522–3536. doi:10.1109/TWC.2022.3219463.

[4] Zhu W, Tuan HD, Dutkiewicz E, Fang Y, Hanzo L. Low-Complexity Pareto-Optimal 3D Beamforming for the Full-Dimensional Multi-User Massive MIMO Downlink. IEEE Transactions on Vehicular Technology. 2023;72(7):8869–8885. doi:10.1109/TVT.2023.3245539.

[5] Singh J, Jagannatham AK, Hanzo L. Geometric Mean Rate Maximization in RIS-Aided mmWave ISAC Systems Relying on a Non-Diagonal Phase Shift Matrix. IEEE Open Journal of the Communications Society. 2025;6:4756–4771. doi:10.1109/OJCOMS.2025.3573196.

[6] Yu H, Tuan HD, Dutkiewicz E, Poor HV, Hanzo L. Maximizing the Geometric Mean of User-Rates to Improve Rate-Fairness: Proper vs. Improper Gaussian

Signaling. IEEE Transactions on Wireless Communications. 2022;21(1):295–309. doi:10.1109/TWC.2021.3095174.

[7] Golub GH, Van Loan CF. Matrix Computations - 4th Edition. Philadelphia, PA: Johns Hopkins University Press; 2013. Available from: `https://epubs.siam.org/doi/abs/10.1137/1.9781421407944`.

[8] Prentice JSC. Efficiency of the multisection method. Journal of Computational Mathematics and Data Science. 2024;13:100106. doi:https://doi.org/10.1016/j.jcmds.2024.100106.

[9] Hong M, Wang X, Razaviyayn M, Luo ZQ. Iteration complexity analysis of block coordinate descent methods. Math Program. 2017;163(1–2):85–114. doi:10.1007/s10107-016-1057-8.

[10] Ayach OE, Rajagopal S, Abu-Surra S, Pi Z, Heath RW. Spatially Sparse Precoding in Millimeter Wave MIMO Systems. IEEE Transactions on Wireless Communications. 2014;13(3):1499–1513. doi:10.1109/TWC.2014.011714.130846.

[11] Molisch AF, Ratnam VV, Han S, Li Z, Nguyen SLH, Li L, et al. Hybrid Beamforming for Massive MIMO: A Survey. IEEE Communications Magazine. 2017;55(9):134–141. doi:10.1109/MCOM.2017.1600400.

[12] Wu Q, Zhang R. Intelligent Reflecting Surface Enhanced Wireless Network via Joint Active and Passive Beamforming. IEEE Transactions on Wireless Communications. 2019;18(11):5394–5409. doi:10.1109/TWC.2019.2936025.

[13] Basar E, Di Renzo M, De Rosny J, Debbah M, Alouini MS, Zhang R. Wireless Communications Through Reconfigurable Intelligent Surfaces. IEEE Access. 2019;7:116753–116773. doi:10.1109/ACCESS.2019.2935192.

[14] Song N, Yang T, Sun H. Overlapped Subarray Based Hybrid Beamforming for Millimeter Wave Multiuser Massive MIMO. IEEE Signal Processing Letters. 2017;24(5):550–554. doi:10.1109/LSP.2017.2681689.

[15] Chou PH, Zheng BR, Huang WJ, Saad W, Tsao Y, Chang RY. Deep Reinforcement Learning-Based Precoding for Multi-RIS-Aided Multiuser Downlink Systems With Practical Phase Shift. IEEE Wireless Communications Letters. 2025;14(1):23–27. doi:10.1109/LWC.2024.3482720.

[16] Lin T, Cong J, Zhu Y, Zhang J, Ben Letaief K. Hybrid Beamforming for Millimeter Wave Systems Using the MMSE Criterion. IEEE Transactions on Communications. 2019;67(5):3693–3708. doi:10.1109/TCOMM.2019.2893632.

[17] Zhao X, Lin T, Zhu Y, Zhang J. Partially-Connected Hybrid Beamforming for Spectral Efficiency Maximization via a Weighted MMSE Equivalence. IEEE Transactions on Wireless Communications. 2021;20(12):8218–8232. doi:10.1109/TWC.2021.3091524.

[18] Majidzadeh M, Kaleva J, Tervo N, Pennanen H, Tölli A, Latva-aho M. Hybrid Beamforming for mm-Wave Massive MIMO Systems with Partially Connected RF Architecture. Wireless Personal Communications. 2024;136(4):1947–1979. doi:10.1007/s11277-024-11026-1.

[19] Zhu Y, Bo Z, Li M, Liu Y, Liu Q, Chang Z, et al. Deep Reinforcement Learning based Joint Active and Passive Beamforming Design for RIS-Assisted MISO Systems. In: 2022 IEEE Wireless Communications and Networking Conference (WCNC); 2022. p. 477–482.

[20] Hassan K, Masarra M, Zwingelstein M, Dayoub I. Channel Estimation Techniques for Millimeter-Wave Communication Systems: Achievements and Challenges. IEEE Open Journal of the Communications Society. 2020;1:1336–1363. doi:10.1109/OJCOMS.2020.3015394.
