# Supplementary material for: Scalable RIS-aided hybrid beamforming for mmWave systems enables multiple sub-array architectures: A Geometric Mean Approach
Source: PLoS One. 2026 Aug 21;21(8):e0354965. doi: 10.1371/journal.pone.0354965 (PMC13496462; doi:10.1371/journal.pone.0354965)
Supplement: Supplementary file 4 [file pone.0354965.s005.pdf]

Response to the Reviewers

Original Manuscript ID: PONE-D-25-56680
Original Article Title: Scalable RIS-aided Hybrid Beamforming for mmWave Systems enables Multiple Sub-array Architectures: A Geometric Mean Approach
Authors: Hoang M. Tran, Tung V. Dinh, Hoa T. Nguyen, Huy T. Nguyen

Dear Editor and Reviewers,

We appreciate the time you have dedicated to handling our manuscript and for giving us the opportunity to revise and resubmit it. In this revision, we have made the following updates:

- We have revised and clarified the system setup, along with the application of the considered system.
- We have updated and improved the description of the signal detection process at the target to make it more readable to readers.
- We have improved the discussion about the non-convexity and challenges in solving the problem of maximizing the mutual information.
- We have added a discussion on the convergence characteristic and clarified the accuracy of the result achieved by the proposed algorithm in solving the problem of maximizing the mutual information.
- We have rechecked and proofread the whole manuscript.

In the following, we provide point-to-point responses to the comments and actions that we have taken to address those issues. The raised comments and our answers are described in *italic* font and roman font, respectively. Unless stated otherwise, all the numbered items (figures, equations, etc.) in this response letter refer to those in the revised manuscript.

Furthermore, in the revised manuscript, the major changed parts, such as updated related works, more detailed explanations of the proposed system model, new numerical results, and more insightful discussions have been highlighted in blue color. Other minor corrections and rearrangements have not been highlighted. Please also note that in this response, we provide many relevant references to strongly support the correctness of some of our arguments. However, highly correlated references are cited in the revised manuscript.

Further, we would like the Editor to assign our article to the same set of Reviewers for further review. We do hope that the Editor and Reviewers find our explanations/justifications and revisions/improvements satisfactory.

Yours sincerely,

Executive Editor

General comments: *After careful consideration of the revised manuscript and the reviewers' comments, I invite you to submit a further revised version of the manuscript. Although two reviewers consider the manuscript acceptable and acknowledge that their previous concerns have been addressed, one reviewer has identified several remaining issues that must be resolved before the manuscript can be considered for publication. In addition, concerns remain regarding statistical rigor and data availability, which are part of PLOS ONE's publication criteria. The following revisions are required:*

Consider 1 – *First, the response letter should be strengthened. The authors should not only refer to revised manuscript sections, but should also provide concise and explicit explanations of the main technical rationale, design choices, and methodological decisions.*

Authors' Response: We thank the Editor for this insightful suggestion. In the revised manuscript, we have explicitly clarified the key technical motivations underlying our core design choices, as detailed below:

- **Use of GM objective**: The geometric mean objective is selected because RIS-assisted mmWave channels exhibit structural user disparity due to blockage and path-loss heterogeneity. Unlike sum-rate maximization, which prioritizes dominant users, the GM objective penalizes near-zero user rates, thereby ensuring non-collapse of individual user performance and improving instantaneous fairness.
- **Adoption of OSA-based hybrid beamforming**: The OSA architecture is chosen as a middle-ground design that balances spatial degrees of freedom and hardware complexity. It generalizes both fully-connected (high flexibility) and sub-array (low complexity) structures, enabling scalable implementation under RF-chain constraints.
- **Riemannian manifold optimization**: The manifold framework is employed to directly handle unit-modulus constraints without relaxation (e.g., SDR), avoiding high computational complexity and enabling a hardware-consistent solution with discrete phase projection.
- **BCD decomposition**: The problem is strongly non-convex with coupled variables. The BCD framework provides a tractable approach with monotonic convergence guarantees while preserving problem structure.

These clarifications have been explicitly included in both the current version and the previous response letter, rather than relying solely on references to revised sections of the manuscript.

The above design choices are motivated by both propagation-level and implementation-level considerations. First, the GM-rate objective is adopted because it provides a fairness-aware alternative to conventional sum-rate maximization by penalizing near-zero user rates

(A) (B)

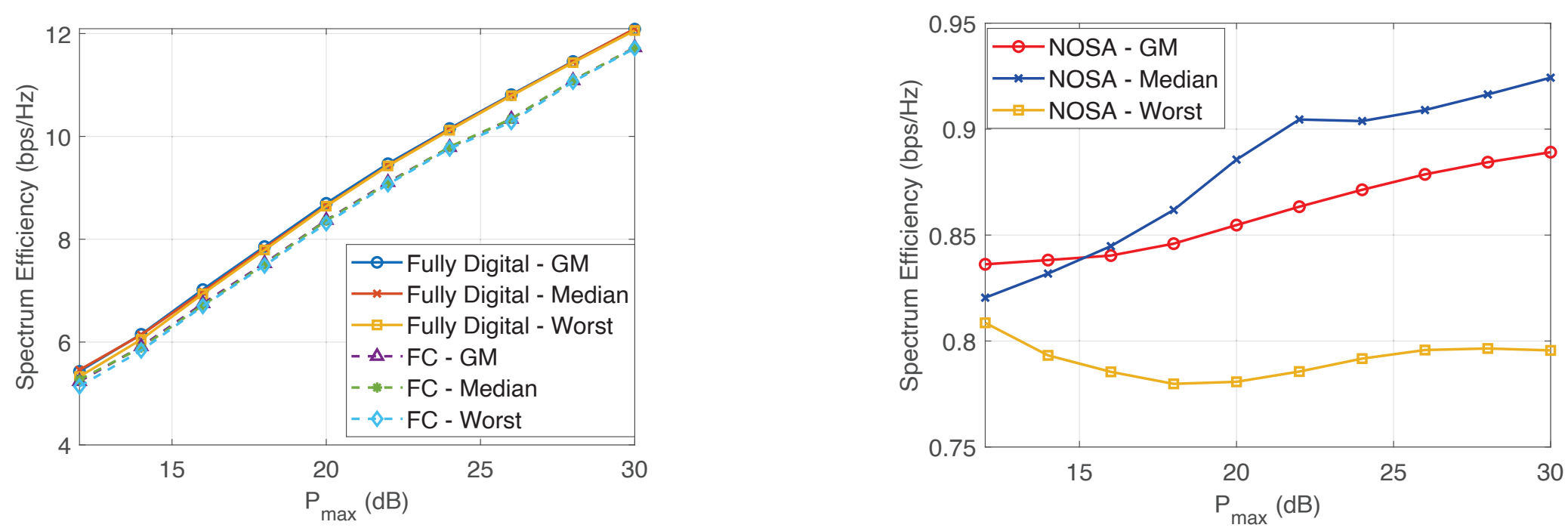

Figure 1: Rate distribution comparison between geometric mean (GM) and max–min designs. (A) Fully connected (FC) architecture. (B) Non-overlapped subarray (NOSA) architecture.

and avoiding rate collapse among users with weak composite channels [1]. This property is particularly relevant to RIS-assisted mmWave systems, where blockage and path-loss heterogeneity may create persistent user-rate disparity. Second, the OSA-based hybrid beamforming architecture is selected because it offers a practical compromise between the high spatial flexibility of fully-connected architectures and the lower hardware complexity of non-overlapped sub-array structures [2]. This makes OSA suitable for scalable mmWave deployments under RF-chain and phase-shifter constraints. Third, since both analog beamforming coefficients and RIS phase shifts are subject to unit-modulus constraints, the feasible set naturally admits a manifold structure. Riemannian manifold optimization therefore enables direct optimization over the feasible set without relying on high-dimensional relaxation methods [3]. Finally, because the resulting GM-rate maximization problem involves strongly coupled digital precoding, analog beamforming, and RIS phase variables, a BCD framework is adopted to decompose the original non-convex problem into tractable subproblems while preserving monotonic objective improvement [4].

Consider 2 – *Second, the motivation for using the geometric mean objective should be supported more quantitatively. The revised manuscript should include additional evidence, such as fairness metrics, rate distribution comparisons, or performance-gap analysis, to clarify why the geometric mean objective is necessary compared with sum-rate or proportional fairness objectives.*

Authors' Response: We appreciate this valuable comment and have strengthened the quantitative justification of the GM objective using simulation results already present in the manuscript. Specifically, the quantitative justification of the GM objective can be found from Figure. 1 (corresponding to Fig. 6 in the revised manuscript) and Figure. 2 (corresponding to Fig. 7(A) in the revised manuscript), which both illustrates the rate distribution comparison between overall GM rate of users and rate of specific individual.

From these figures, we make several clarifications on quantitative validation of fairness improvement of GM objective in comparision to traditional approaches such as sum-rate or

proportional fairness constraint:

- Fairness improvement (rate distribution): Fig. 1B shows that the GM objective significantly improves the worst-user rate compared to median and dominant users for the NOSA case in the low SNR region while striking a balance between overall performance and maintaining an acceptable performance for the worst-user rate in the high SNR region, demonstrating the ability to reduce rate disparity across users. In detail, the worst-user rate maintains a stable performance around 0.8 bps/Hz for increasing level of P_{max} while the median-user rate and GM rate increasing from 0.85 to 0.9 bps/Hz. Thus we can see that even in the low flexibility structure as NOSA, maximizing GM rate can still ensure that no user-rate collapse from unfair resource distribution.

- Alignment of user rates: In FC and FD architectures, the worst-user, median-user, and GM curves closely align, indicating that the GM formulation effectively enforces balanced rate allocation across users. This is because in highly flexible architectures such as FC and FD, more spatial degree of freedom can be offered to improve the performance of the worst-user rate, which in return improve the overall GM. As the maximum of GM can be obtained when all user archive the same data rate, the worst-user rate is improved until aligning with the overall GM. This phenomenon can be seen in both Figure. 1 and Figure. 2

- Implicit fairness metric: The GM objective is mathematically equivalent to maximizing the product of user rates, which is closely related to proportional fairness but avoids additional constraint complexity. The difficulty of transitioning from GM to proportional fairness lies in the fact that proportional fairness is typically defined as a property of a solution rather than an objective. To enforce proportional fairness directly, one would need to solve a variational inequality or incorporate an infinite family of constraints, as discussed previously. The GM formulation bypasses these difficulties entirely by recognizing that maximizing the rate product yields approximately the same solution as the min-max objective while providing a tractable and scalable solution that implicitly improves fairness without any of the associated computational complexity.

Additionally, we highlight that the novelty behind choosing the geometric mean (GM) of data rates as the objective function lies in the trade-off of rate fairness among users with the overall network performance. Specifically, the overall network performance is often formulated as the weighted sum rate (SR) optimization problem in the literature [5][6]. However, this approach has the deficiency of allocating a large fraction of the sum-rate to a few users having good channel conditions, while leaving the rest of the users with almost zero rates. This motivates the formulation of fairness based on GM rate due to several key aspects:

- The geometric mean over $\prod_{k=1}^{K}(1+\mathrm{SINR}_k)$ is equivalent to the unweighted sum rate formulation, i.e. $\sum_{k=1}^{K} R_k$, since $\sum_{k=1}^{K}\log_2(1+\mathrm{SINR}_k) = \log_2\left(\prod_{k=1}^{K}(1+\mathrm{SINR}_k)\right)$.

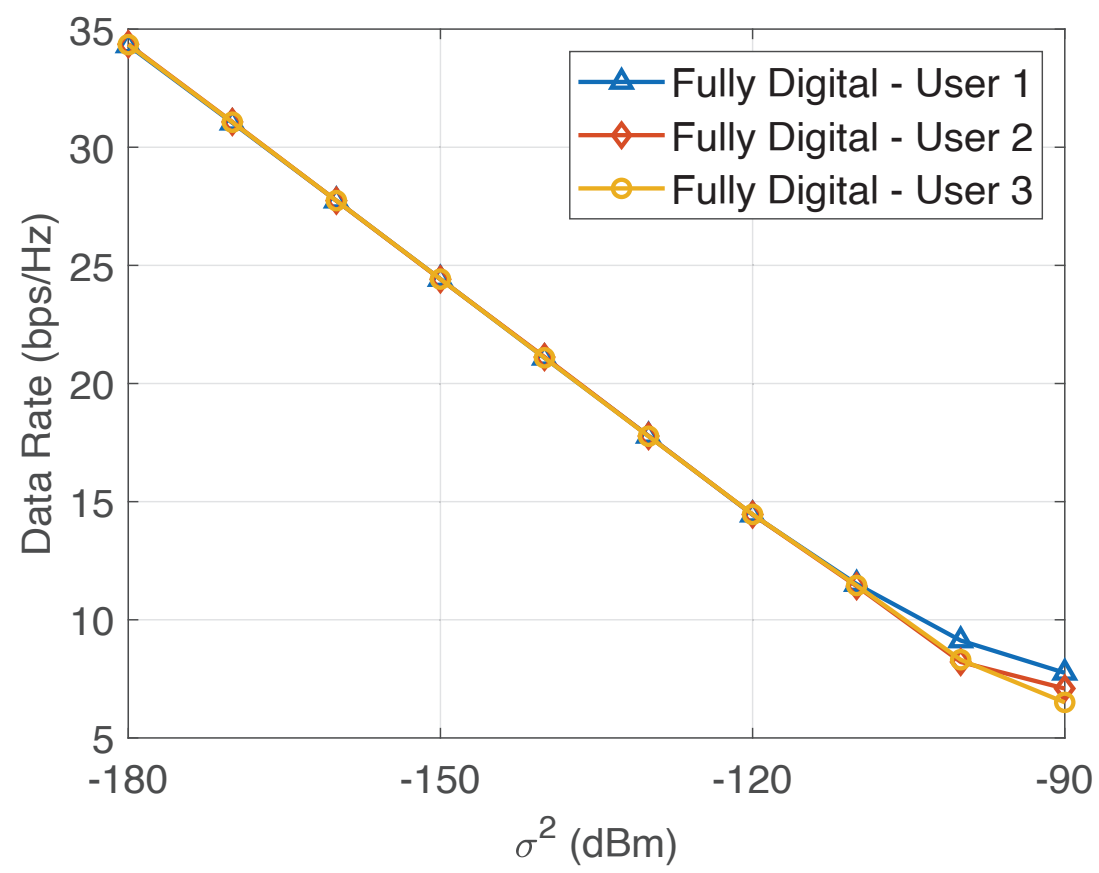

Figure 2: Spectral efficiency of individual users versus noise power.

As such, the objective function $\max(\sum_{k=1}^{K} R_k) = \max \log_2 \left(\prod_{k=1}^{K}(1 + \text{SINR}_k)\right)$ is equivalent to $\max \left(\prod_{k=1}^{K}(1 + \text{SINR}_k)\right)$.

- To achieve the fairness target, one can employ the max-min rate objective or proportional rate constraint for maximizing the rate of the users having poor channel conditions. However, the max-min rate approach leads to an undesirable degradation in the overall sum rate (SR) of the system while the proportional rate constraint introduces additional nonconvexity and computation complexity to the hybrid beamforming scheme. Furthermore, the authors in [7] pointed out that with suitable choice of parameters, the standard proportional fairness can be regarded as a max-min optimization problem. As an alternative solution to balance fairness and high sum rate while introduce little computation difficulty, the geometric mean (GM) rate maximization is proposed as a promising solution in the literature [1][8][9].

These clarifications are now explicitly discussed in the introduction of the revised manuscript as follows:

Geometric mean (GM) rate maximization provides a principled mechanism to address this fairness and reliability gap by explicitly balancing individual user rates [24]. Unlike sum-rate objectives, which are insensitive to individual rate collapse, GM-rate penalizes vanishing user rates and enforces balanced performance across all users within a single transmission interval. Moreover, the GM-rate achieves a more favorable trade-off between fairness guarantees and overall performance maximization compared to conventional fairness paradigms, such as the max-min fairness objective or proportional rate constraints. Unlike the max-min objective, which heavily favors fairness at the expense of efficiency, or proportional rate constraints, which incur additional computational complexity, the GM objective offers a tractable and scalable solution that implicitly improves fairness without introducing any associated complex constraints. This property is particularly important in quasi-static RIS-assisted mmWave

systems, where limited scheduling diversity and reliance on reflected paths prevent fairness from being achieved through long-term averaging. Under OSA-based hybrid beamforming, GM-rate aligns with reduced spatial degrees of freedom by prioritizing robustness and service continuity over peak throughput.

Together we also expanded the discussion on Fig. 6 in the revised manuscript as follows:

Fig. 6 illustrates the relationship between the surrogate objective and user fairness. Specifically, we evaluate the worst-user rate and the median-user rate under the same simulation settings to verify that the GM design effectively improves the performance of the weakest user relative to FC and NOSA. As shown in Fig. 6(A), the performance curve of each target user aligns to the geometric mean curve for both FD and FC architectures, confirming the fairness traits of GM design. However, Fig. 6(B) shows a considerable separation between the three benchmarks. This phenomenon arises from the trade-off between architectural complexity and QoS performance in the NOSA scheme, which provides a limited degree of freedom for analog beamforming to suppress inter-user interference. Nevertheless, the GM objective significantly improves the worst-user rate compared to median and dominant users for the NOSA case in the low SNR region while striking a balance between overall performance and maintaining an acceptable performance for the worst-user rate in the high SNR region, demonstrating the ability to reduce rate disparity across users. In detail, the worst-user rate maintains a stable performance around 0.8 bps/Hz for increasing level of $P_{\max}$ while the median-user rate and GM rate increasing from 0.85 to 0.9 bps/Hz. Nevertheless, the GM objective significantly improves the worst-user rate compared to the median and dominant users for the NOSA case in the low SNR region, while striking a balance between overall performance and maintaining an acceptable worst-user rate in the high SNR region, thereby demonstrating its ability to reduce rate disparity across users. Concretely, the worst-user rate remains stable at approximately 0.8 bps/Hz as $P_{\max}$ increases, whereas the median-user rate and the GM rate increase from 0.85 to 0.9 bps/Hz. Thus, even in a low spatial degree of freedom structure such as NOSA, maximizing the GM rate prevents user-rate collapse due to unfair resource distribution. Consequently, we note that a restricted spatial OSA configuration intensifies the trade-off between overall rate and user fairness, leading to a misalignment between the GM rate and the individual user rates compared to a more flexible configuration.

Consider 3 – *Third, the authors should more clearly distinguish the primary technical contribution from incremental improvements over existing RIS-aided hybrid beamforming methods. In particular, the revision should explicitly explain the main methodological novelty, how it differs from standard BCD-based, manifold-optimization-based, analytical, or learning-based approaches, and what specific advantage is obtained by the proposed framework.*

Authors' Response: We thank the Editor for this important request and clarify our contributions explicitly:

- **Primary technical novelty**: The core novelty lies in the unified integration of GM-rate maximization, OSA-based hybrid beamforming, and discrete RIS phase optimization

within a Riemannian manifold framework, which has not been jointly addressed in prior works.

- **Methodological difference from existing RIS-HBF methods**: Existing works predominantly optimize sum-rate or weighted sum-rate, assume fully digital architectures, or use SDR or heuristic alternating optimization. In contrast, our method targets fairness-aware GM optimization, operates under practical OSA hybrid constraints, employs manifold optimization instead of convex relaxation, and incorporates discrete phase projection per iteration.

- **Unique advantage beyond standard BCD/manifold approaches**: A novel OSA-consistent factorization that maps fully-digital solutions to hybrid realizations without matrix lifting. Dynamic GM-based weighting mechanism in BCD that differs from fixed weights in sum-rate formulations. Interference-aware initialization, yielding up to 18.18% performance improvement and faster convergence.

We have refined the main contribution discussion along with "Novelty Statement" section to explicitly separate (i) conceptual novelty, (ii) methodological differentiation, and (iii) practical advantages as follows:

1. **Conceptual novelty**: We formulate a fairness-driven GM-rate maximization problem for RIS-assisted mmWave systems with OSA-based hybrid beamforming and discrete RIS phases, thereby extending the applicable configuration concept and explicitly addressing the reliability gap inherent in sum-rate-centric designs prevalent in the existing literature.

2. **Methodological differentiation**: We propose an OSA-consistent Riemannian manifold factorization that maps the fully-digital GM-optimal precoder benchmark into a realizable hybrid analog-digital structure without resorting to matrix lifting or semidefinite relaxation. We apply a dynamic GM-based weighting approximation in BCD that differs from fixed weights in sum-rate formulations. Furthermore, instead of directly applying the Riemannian manifold conjugate descent, we iteratively search for the optimal digital beamforming matrix via a closed-form expression while reformulating the optimal analog beamforming matrix as a manifold factorization problem.

3. **Practical advantages**: We develop an interference-aware warm-start strategy that stabilizes the power-constrained precoder update and accelerates convergence by up to 18.18% compared with random initialization. Extensive simulations demonstrate that OSA-based architectures with moderate RF-chain counts and low-resolution (3-bit) phase shifters at both the BS and RIS can closely approach continuous-phase performance, validating the practicality of the proposed framework.

Novelty Statement. To the best of our knowledge, this work represents the first principled attempt to systematically bridge three previously disconnected aspects in RIS-assisted mmWave systems, namely fairness-oriented performance optimization by leveraging a GM

objective, practical OSA-based hybrid beamforming architectures, and discrete phase control within a unified Riemannian manifold optimization framework. In terms of the conceptual system model, unlike the prevailing literature that prioritizes sum-rate maximization or relies on idealized fully-digital precoding assumptions [15-17], the proposed framework directly addresses the structural rate imbalance intrinsic to RIS-aided mmWave propagation with the influence of hybrid beamforming, where blockage sensitivity and geometry-dependent attenuation fundamentally limit user-level reliability [12,13]. By integrating GM-rate maximization with an OSA-consistent hybrid realization under discrete phase constraints, this work fills a critical research gap and paves a realizable methodology toward fairness-critical RIS-assisted deployments under stringent hardware limitations [24].

Consider 4 – *Fourth, assumptions that were previously described as being outside the scope of the paper, such as dynamic environments, robustness, and extended performance metrics, should be discussed more carefully. The authors do not necessarily need to solve all of these issues in the present work, but they should provide a clear analytical or conceptual justification of the assumptions and explain how these limitations affect the applicability and generalizability of the proposed method.*

Authors' Response: We have revised the manuscript to clarify the technical implications of the assumptions rather than simply treating them as outside the scope.

- The quasi-static channel assumption is required because the proposed BCD-based framework jointly updates the fully digital equivalent precoder, the RIS phase matrix, and the OSA hybrid factorization. These variables are strongly coupled, and the monotonic improvement property of the alternating procedure is meaningful only when the effective cascaded channel remains unchanged during one optimization block. Therefore, the method is most applicable to slowly varying RIS-assisted mmWave scenarios, such as indoor hotspots, fixed wireless access, and low-mobility users, where the channel coherence interval is sufficiently long for iterative beamforming updates. In highly mobile scenarios, the current formulation should be interpreted as a per-channel-block optimization benchmark rather than a real-time tracking algorithm.

- For CSI uncertainty, we now clarify that the algorithm is not explicitly robust in the worst-case or stochastic-robust optimization sense. Nevertheless, Fig. 8 in the revised manuscript quantifies the sensitivity of the GM-rate to channel perturbations. The observed gradual degradation indicates that moderate CSI errors do not immediately destroy the fairness-oriented gain, although severe CSI mismatch would reduce the reliability of both the precoder and RIS phase updates. This limitation motivates robust extensions in which the GM objective is optimized under bounded or statistical CSI error models.

- Regarding energy efficiency and latency, these metrics are not omitted because they are unimportant, but because they require additional models that are not part of the present physical-layer formulation. Energy efficiency would require explicit circuit-power

models for RF chains, phase shifters, RIS control links, and baseband processing, while latency would require channel training, feedback, scheduling, and convergence-time models. Incorporating these factors would change the problem from fairness-oriented beamforming into a cross-layer multi-objective design. The present study therefore isolates the fundamental trade-off among GM-rate fairness, OSA hardware constraints, and discrete RIS phase control.

We have added a dedicated discussion in the Conclusion to state these applicability limits and to identify robust CSI-aware optimization, low-complexity tracking for dynamic channels, and fairness-energy-efficiency trade-offs for a concrete extension as follows:

In this paper, we investigated the spectral efficiency of an RIS-aided scalable hybrid beamforming system operating over the mmWave channel. Given the adopted OSA hybrid architecture at BS, we proposed a joint optimization algorithm to maximize the GM of users' achievable rates. Based on existing works, we divide the hybrid beamforming problem into the FD beamforming sub-problem and the matrix decomposition problem. We then adopt the iterative BCD method and RCG to solve the resulting optimization problems. An effective initialization method is proposed to enhance the convergence rate of the algorithm. Numerical results demonstrate the effectiveness of the proposed framework in improving fairness-oriented spectral efficiency, convergence behavior, and architectural trade-offs under practical hardware constraints. Specifically, we observed an 18.18% objective value improvement compared to existing methods and identified suitable hardware phase resolutions for RIS-assisted hybrid beamforming systems.

The proposed framework is specifically designed to optimize fairness-oriented spectral efficiency under hardware constraints. Accordingly, the conclusions of this work are restricted to the evaluated aspects, including GM-rate performance, convergence behavior, fairness characteristics, architectural trade-offs, and sensitivity to CSI uncertainty under the considered system model. While the impact of CSI uncertainty has been partially examined through CSI perturbation analysis, energy-efficiency optimization, latency-aware design, and robust formulations for highly dynamic environments remain topics for further investigation.

Consider 5 – *Fifth, the experimental evaluation should be strengthened or the conclusions should be narrowed accordingly. If additional performance evaluations are feasible, the authors should include them. If not, the manuscript should explicitly limit its claims to the evaluation settings that have actually been tested.*

Authors' Response: We agree that the experimental evaluation does not aim to cover all possible system-level metrics. The primary objective of this work is to investigate fairness-oriented spectral efficiency in RIS-assisted hybrid beamforming systems under practical hardware constraints.

1. Within this scope, the numerical results already assess the proposed framework from several complementary perspectives, including: i) GM-rate performance, ii) convergence behavior (Fig. 4 in the revised manuscript), iii) user-rate distribution and fairness

characteristics (Fig. 6 in the revised manuscript), iv) comparison with different hybrid architectures (FC and NOSA), v) the impact of RF-chain configuration and noise conditions, and vi) sensitivity to CSI uncertainty (Fig. 8 in the revised manuscript). Together, these results provide a comprehensive evaluation of the fairness-performance trade-off targeted by the proposed design.

2. Rather than expanding into unrelated metrics, we refine the claims to align with the paper's primary goal: "*The proposed framework is specifically designed to optimize fairness-oriented spectral efficiency under hardware constraints"* as follows:

The proposed framework is specifically designed to optimize fairness-oriented spectral efficiency under hardware constraints. Accordingly, the conclusions of this work are restricted to the evaluated aspects, including GM-rate performance, convergence behavior, fairness characteristics, architectural trade-offs, and sensitivity to CSI uncertainty under the considered system model. While the impact of CSI uncertainty has been partially examined through CSI perturbation analysis, energy-efficiency optimization, latency-aware design, and robust formulations for highly dynamic environments remain topics for further investigation.

Consider 6 – *Sixth, the positioning of the proposed method within the existing literature should be improved through a deeper comparative discussion with representative analytical and learning-based methods.*

Authors' Response: We have strengthened the discussion to better position the proposed framework relative to both analytical and learning-based approaches.

1. Compared with conventional analytical methods such as SDR- and WMMSE-based designs, the proposed framework directly accommodates the OSA hybrid architecture and discrete RIS phase constraints without resorting to matrix lifting or semidefinite relaxation. This avoids the dimensional growth associated with lifted formulations and facilitates the treatment of large-scale RIS-assisted systems.

2. Compared with AI- and DRL-based approaches, the proposed method does not require offline training and maintains an explicit link between the optimization objective and user fairness through GM-rate maximization. In addition, the optimization process remains transparent and interpretable, since each update is directly associated with the underlying system model and constraints.

3. At the same time, the proposed framework inherits several limitations common to model-based optimization. It relies on CSI availability, converges to a stationary point rather than a globally optimal solution, and is most suitable for scenarios in which the channel remains approximately constant during the optimization process. In contrast, learning-based approaches may offer advantages in highly dynamic environments once sufficiently trained.

Therefore, the proposed approach should be viewed as a fairness-oriented and hardware-consistent model-based solution that complements, rather than replaces, existing analytical and learning-based frameworks. To address this comment, we have expanded the discussion after Table 1 in the Introduction as follows:

As summarized in Table 1, the proposed framework occupies an intermediate position between conventional optimization-based and learning-based beamforming designs. Compared with SDR- and WMMSE-based approaches, it directly accommodates the OSA hybrid architecture and discrete RIS phase constraints without relying on semidefinite relaxation or matrix lifting, while directly handling the structured constraints imposed by the hardware architecture. Compared with AI- and DRL-based methods, the proposed approach does not require offline training and explicitly incorporates user fairness through GM-rate maximization. Additionally, the proposed framework inherits several limitations common to model-based optimization, including its dependence on CSI availability and convergence to a stationary point of a non-convex problem. Moreover, its iterative nature makes it more suitable for scenarios where channel variations occur on a slower time scale than the optimization process. Therefore, the proposed framework should be viewed as a fairness-oriented and hardware-consistent model-based solution that complements, rather than replaces, existing analytical and learning-based approaches.

Consider 7 – *Finally, the authors must address the remaining data availability and reproducibility concerns. Please ensure that all data, code, simulation parameters, or sufficient information needed to reproduce the results are made available in accordance with PLOS ONE's data policy, unless a valid restriction applies. The authors should also carefully check the reference list and remove or correct repeated references, including the repeated entries noted by the reviewer.*

Authors' Response: We thank the Editor for this important comment. We have revised the manuscript to comply with the PLOS ONE Data Availability Policy. Since this study is entirely simulation-based, the manuscript now provides all system models, optimization procedures, simulation parameters, and implementation details necessary to reproduce the reported results. The corresponding Data Availability Statement has also been updated accordingly.

We carefully reviewed both the reference list and all in-text citations, corrected repeated citations, and verified the consistency of all references throughout the manuscript. Finally, we have included a detailed point-by-point response, a tracked-changes manuscript, and a clean revised manuscript, as requested.

References

[1] Yu H, Tuan HD, Dutkiewicz E, Poor HV, Hanzo L. Maximizing the Geometric Mean of User-Rates to Improve Rate-Fairness: Proper vs. Improper Gaussian Signaling. IEEE Transactions on Wireless Communications. 2022;21(1):295–309. doi:10.1109/TWC.2021.3095174.

[2] Song N, Yang T, Sun H. Overlapped Subarray Based Hybrid Beamforming for Millimeter Wave Multiuser Massive MIMO. IEEE Signal Processing Letters. 2017;24(5):550–554. doi:10.1109/LSP.2017.2681689.

[3] Li S, Chen J, Xu Y, Li S, Yao L, Wang J, et al. Riemannian Manifold Optimization for Advanced Wireless Communications: Fundamentals and Applications. arXiv preprint arXiv:260208225. 2026;.

[4] Razaviyayn M, Hong M, Luo ZQ. A Unified Convergence Analysis of Block Successive Minimization Methods for Nonsmooth Optimization. SIAM Journal on Optimization. 2013;23(2):1126–1153. doi:10.1137/120891009.

[5] Tam HHM, Tuan HD, Ngo DT. Successive Convex Quadratic Programming for Quality-of-Service Management in Full-Duplex MU-MIMO Multicell Networks. IEEE Transactions on Communications. 2016;64(6):2340–2353. doi:10.1109/TCOMM.2016.2550440.

[6] Jiang J, Yuan Y, Zhen L. Multi-User Hybrid Precoding for Dynamic Subarrays in mmWave Massive MIMO Systems. IEEE Access. 2019;7:101718–101728. doi:10.1109/ACCESS.2019.2929927.

[7] Dong F, Liu F, Cui Y, Wang W, Han K, Wang Z. Sensing as a Service in 6G Perceptive Networks: A Unified Framework for ISAC Resource Allocation. IEEE Transactions on Wireless Communications. 2023;22(5):3522–3536. doi:10.1109/TWC.2022.3219463.

[8] Zhu W, Tuan HD, Dutkiewicz E, Fang Y, Hanzo L. Low-Complexity Pareto-Optimal 3D Beamforming for the Full-Dimensional Multi-User Massive MIMO Downlink. IEEE Transactions on Vehicular Technology. 2023;72(7):8869–8885. doi:10.1109/TVT.2023.3245539.

[9] Singh J, Jagannatham AK, Hanzo L. Geometric Mean Rate Maximization in RIS-Aided mmWave ISAC Systems Relying on a Non-Diagonal Phase Shift Matrix. IEEE Open Journal of the Communications Society. 2025;6:4756–4771. doi:10.1109/OJCOMS.2025.3573196.
