# Supplementary material for: Scalable RIS-aided hybrid beamforming for mmWave systems enables multiple sub-array architectures: A Geometric Mean Approach
Source: PLoS One. 2026 Aug 21;21(8):e0354965. doi: 10.1371/journal.pone.0354965 (PMC13496462; doi:10.1371/journal.pone.0354965)
Supplement: Peer review history

# Peer review: Decision Letter 0

**DOI:** 10.1371/journal.pone.0354965.r001

**Authors:** Mohammad Reza Ghavidel Aghdam

5 Jan 2026

PONE-D-25-56680

Scalable RIS-aided Hybrid Beamforming for mmWave Systems enables Multiple Sub-array Architectures: A Geometric Mean Approach

PLOS One

Dear Dr. Nguyen Thanh,

Thank you for submitting your manuscript to PLOS ONE. After careful consideration, we feel that it has merit but does not fully meet PLOS ONE’s publication criteria as it currently stands. Therefore, we invite you to submit a revised version of the manuscript that addresses the points raised during the review process.

Please submit your revised manuscript by Feb 19 2026 11:59PM. If you will need more time than this to complete your revisions, please reply to this message or contact the journal office at plosone@plos.org. When you're ready to submit your revision, log on to https://www.editorialmanager.com/pone/ and select the 'Submissions Needing Revision' folder to locate your manuscript file.

Please include the following items when submitting your revised manuscript:

- A letter that responds to each point raised by the academic editor and reviewer(s). You should upload this letter as a separate file labeled 'Response to Reviewers'.
- A marked-up copy of your manuscript that highlights changes made to the original version. You should upload this as a separate file labeled 'Revised Manuscript with Track Changes'.
- An unmarked version of your revised paper without tracked changes. You should upload this as a separate file labeled 'Manuscript'.

If you would like to make changes to your financial disclosure, please include your updated statement in your cover letter. Guidelines for resubmitting your figure files are available below the reviewer comments at the end of this letter.

If applicable, we recommend that you deposit your laboratory protocols in protocols.io to enhance the reproducibility of your results. Protocols.io assigns your protocol its own identifier (DOI) so that it can be cited independently in the future. For instructions see: https://journals.plos.org/plosone/s/submission-guidelines#loc-laboratory-protocols. Additionally, PLOS ONE offers an option for publishing peer-reviewed Lab Protocol articles, which describe protocols hosted on protocols.io. Read more information on sharing protocols at https://plos.org/protocols?utm_medium=editorial-email&utm_source=authorletters&utm_campaign=protocols.

We look forward to receiving your revised manuscript.

Kind regards,

Mohammad Reza Ghavidel Aghdam, Ph.D.

Academic Editor

PLOS One

Journal Requirements:

When submitting your revision, we need you to address these additional requirements.

1. Please ensure that your manuscript meets PLOS ONE's style requirements, including those for file naming. The PLOS ONE style templates can be found at

https://journals.plos.org/plosone/s/file?id=wjVg/PLOSOne_formatting_sample_main_body.pdf and

https://journals.plos.org/plosone/s/file?id=ba62/PLOSOne_formatting_sample_title_authors_affiliations.pdf

2. Please note that PLOS One has specific guidelines on code sharing for submissions in which author-generated code underpins the findings in the manuscript. In these cases, we expect all author-generated code to be made available without restrictions upon publication of the work. Please review our guidelines at https://journals.plos.org/plosone/s/materials-and-software-sharing#loc-sharing-code and ensure that your code is shared in a way that follows best practice and facilitates reproducibility and reuse.

3. We note that the grant information you provided in the ‘Funding Information’ and ‘Financial Disclosure’ sections do not match.

When you resubmit, please ensure that you provide the correct grant numbers for the awards you received for your study in the ‘Funding Information’ section.

4. Thank you for stating the following financial disclosure:

[This research is funded by Hanoi University of Science and Technology

(HUST) under project number T2023-PC-030.].

Please state what role the funders took in the study.  If the funders had no role, please state: "The funders had no role in study design, data collection and analysis, decision to publish, or preparation of the manuscript."

If this statement is not correct you must amend it as needed.

Please include this amended Role of Funder statement in your cover letter; we will change the online submission form on your behalf.

5. Thank you for stating the following in the Acknowledgments Section of your manuscript:

[This research is funded by Hanoi University of Science and Technology (HUST) under project number T2023-PC-030.]

We note that you have provided funding information that is currently declared in your Funding Statement. However, funding information should not appear in the Acknowledgments section or other areas of your manuscript. We will only publish funding information present in the Funding Statement section of the online submission form.

Please remove any funding-related text from the manuscript and let us know how you would like to update your Funding Statement. Currently, your Funding Statement reads as follows:

[This research is funded by Hanoi University of Science and Technology

(HUST) under project number T2023-PC-030.]

Please include your amended statements within your cover letter; we will change the online submission form on your behalf.

6. We note that your Data Availability Statement is currently as follows: [All relevant data are within the manuscript and its Supporting Information files.]

Please confirm at this time whether or not your submission contains all raw data required to replicate the results of your study. Authors must share the “minimal data set” for their submission. PLOS defines the minimal data set to consist of the data required to replicate all study findings reported in the article, as well as related metadata and methods (https://journals.plos.org/plosone/s/data-availability#loc-minimal-data-set-definition).

For example, authors should submit the following data:

- The values behind the means, standard deviations and other measures reported;

- The values used to build graphs;

- The points extracted from images for analysis.

Authors do not need to submit their entire data set if only a portion of the data was used in the reported study.

If your submission does not contain these data, please either upload them as Supporting Information files or deposit them to a stable, public repository and provide us with the relevant URLs, DOIs, or accession numbers. For a list of recommended repositories, please see https://journals.plos.org/plosone/s/recommended-repositories.

If there are ethical or legal restrictions on sharing a de-identified data set, please explain them in detail (e.g., data contain potentially sensitive information, data are owned by a third-party organization, etc.) and who has imposed them (e.g., an ethics committee). Please also provide contact information for a data access committee, ethics committee, or other institutional body to which data requests may be sent. If data are owned by a third party, please indicate how others may request data access.

7. We note that Figure 1 in your submission contain copyrighted images. All PLOS content is published under the Creative Commons Attribution License (CC BY 4.0), which means that the manuscript, images, and Supporting Information files will be freely available online, and any third party is permitted to access, download, copy, distribute, and use these materials in any way, even commercially, with proper attribution. For more information, see our copyright guidelines: http://journals.plos.org/plosone/s/licenses-and-copyright.

We require you to either (1) present written permission from the copyright holder to publish these figures specifically under the CC BY 4.0 license, or (2) remove the figures from your submission:

1. You may seek permission from the original copyright holder of Figure 1 to publish the content specifically under the CC BY 4.0 license.

We recommend that you contact the original copyright holder with the Content Permission Form (http://journals.plos.org/plosone/s/file?id=7c09/content-permission-form.pdf) and the following text:

“I request permission for the open-access journal PLOS ONE to publish XXX under the Creative Commons Attribution License (CCAL) CC BY 4.0 (http://creativecommons.org/licenses/by/4.0/). Please be aware that this license allows unrestricted use and distribution, even commercially, by third parties. Please reply and provide explicit written permission to publish XXX under a CC BY license and complete the attached form.”

Please upload the completed Content Permission Form or other proof of granted permissions as an "Other" file with your submission.

In the figure caption of the copyrighted figure, please include the following text: “Reprinted from [ref] under a CC BY license, with permission from [name of publisher], original copyright [original copyright year].”

2. If you are unable to obtain permission from the original copyright holder to publish these figures under the CC BY 4.0 license or if the copyright holder’s requirements are incompatible with the CC BY 4.0 license, please either i) remove the figure or ii) supply a replacement figure that complies with the CC BY 4.0 license. Please check copyright information on all replacement figures and update the figure caption with source information. If applicable, please specify in the figure caption text when a figure is similar but not identical to the original image and is therefore for illustrative purposes only.

If the reviewer comments include a recommendation to cite specific previously published works, please review and evaluate these publications to determine whether they are relevant and should be cited. There is no requirement to cite these works unless the editor has indicated otherwise.

Additional Editor Comments:

Thank you for submitting your manuscript on RIS-aided mmWave hybrid beamforming with overlapped subarray architecture. The paper addresses a timely topic and proposes a technically sound optimization framework combining geometric mean rate maximization, block coordinate descent, and Riemannian manifold-based hybrid decomposition. The reviewers acknowledge the mathematical correctness of the approach and the promising numerical gains over existing methods.

However, reviewers have raised several substantial concerns regarding motivation, novelty clarification, contextualization with recent literature, fairness interpretation, algorithmic clarity, and experimental validation. These issues must be addressed thoroughly before the manuscript can be reconsidered for publication. A major revision is therefore required.

Below, we summarize the required revisions by theme. You are expected to respond point-by-point to all reviewer comments in a detailed rebuttal and revise the manuscript accordingly.

1. Motivation and Fairness Justification of the Geometric Mean Objective: A central concern raised by both reviewers is that the choice of geometric mean (GM) rate maximization is insufficiently motivated. You must clearly explain what specific fairness or reliability problem GM rate addresses in RIS-aided hybrid beamforming systems that conventional sum-rate or proportional-fair objectives do not.

2. Novelty Clarification and Contributions: While the contribution list is extensive, it lacks clarity regarding what is fundamentally new versus incremental. Please rewrite the contribution list so that each item reflects a distinct technical innovation, rather than a procedural step.

3. Related Work and Missing Literature (Including AI-Based Methods): The Introduction and related work sections are currently incomplete. Please include recent RIS literature, including but not limited to:

Basar et al., IEEE Access, 2019, Yildirim et al., IEEE TCOM, 2020, Ghavidel Aghdam et al., IEEEMECOM, 2024, Tang et al., IEEE TCOM, 2022, Recent RIS statistical and path-loss modeling works (e.g., indoor/outdoor, mmWave)

Expand the discussion on channel modeling and path-loss assumptions, which are critical in RIS systems. Clearly justify the adopted model and parameters with references.

Importantly, the manuscript must discuss AI-based and reinforcement-learning-based RIS and hybrid beamforming approaches, which are now major competing methodologies.

4. Computational Complexity and Convergence Claims: Claims of improved efficiency and convergence must be better substantiated. Provide a computational complexity analysis in Big- notation and/or in terms of dominant multiplications/additions.

5. Practical Relevance and Deployment Considerations: The manuscript motivates OSA and low-resolution RIS phases but lacks concrete system-level interpretation.

6. Notation, Organization, and English Quality

[Note: HTML markup is below. Please do not edit.]

Reviewers' comments:

Reviewer's Responses to Questions

**Comments to the Author**

1. Is the manuscript technically sound, and do the data support the conclusions?

The manuscript must describe a technically sound piece of scientific research with data that supports the conclusions. Experiments must have been conducted rigorously, with appropriate controls, replication, and sample sizes. The conclusions must be drawn appropriately based on the data presented.

Reviewer #1: Yes

Reviewer #2: Yes

Reviewer #3: Partly

**********

2. Has the statistical analysis been performed appropriately and rigorously?

Reviewer #1: Yes

Reviewer #2: Yes

Reviewer #3: Yes

**********

3. Have the authors made all data underlying the findings in their manuscript fully available?

The PLOS Data policy requires authors to make all data underlying the findings described in their manuscript fully available without restriction, with rare exception (please refer to the Data Availability Statement in the manuscript PDF file). The data should be provided as part of the manuscript or its supporting information, or deposited to a public repository. For example, in addition to summary statistics, the data points behind means, medians and variance measures should be available. If there are restrictions on publicly sharing data—e.g. participant privacy or use of data from a third party—those must be specified.

Reviewer #1: Yes

Reviewer #2: No

Reviewer #3: Yes

**********

4. Is the manuscript presented in an intelligible fashion and written in standard English?

PLOS ONE does not copyedit accepted manuscripts, so the language in submitted articles must be clear, correct, and unambiguous. Any typographical or grammatical errors should be corrected at revision, so please note any specific errors here.

Reviewer #1: Yes

Reviewer #2: Yes

Reviewer #3: Yes

**********

5. Review Comments to the Author

Please use the space provided to explain your answers to the questions above. You may also include additional comments for the author, including concerns about dual publication, research ethics, or publication ethics. (Please upload your review as an attachment if it exceeds 20,000 characters)

Reviewer #1: The paper studies a reconfigurable intelligent surface aided downlink with hybrid beamforming under an overlapped sub array architecture, and it maximizes the geometric mean of user rates by alternating between a fully digital surrogate precoder and RIS phases, while enforcing unit modulus and OSA connectivity. The method uses a pseudoinverse based warm start for precoder, closed form RIS updates, and a normalized least squares update followed by Riemannian conjugate gradient steps. I have the following major comments:

1. The introduction lacks some coherence. Please solidify. Moreover, there are many previous related RIS works such as [REF01,REF02]. Kindly include.

2. Please discuss the novelty behind the optimization of the linear transmit beamformers and the RIS reflecting coefficients.

3. The choice of geometric mean of rates is not well motivated. In particular, the objective is to maximize $\left(\prod_{k=1}^K R_k\right) \quad$ with $R_k=\log _2\left(1+\mathrm{SINR}_k\right)$ in (12). Why is the geometric mean taken over the rates and not over $1+ \mathrm{SINR}_k$ ? The objective $\sum_k \log R_k$ weights users through $\log \log \left(1+\mathrm{SINR}_k\right)$, which compresses differences more than $\sum_k \log \left(1+\mathrm{SINR}_k\right)$. Explain this choice better aligns with your fairness target than the standard proportional fairness form or other types of fairness objectives.

4. Explain how discrete phases are enforced consistently across both blocks and how per iteration the discrete phases are activated.

5. In (14), please provide the exact inequality or tangent majorizer that turns $\sum_k \log R_k$ into this weighted sum at each step that can guarantee all regularity conditions.

6. Discuss the relation between the surrogate objective and fairness across users. In particular, Fig. 4 to Fig. 7 plot the GM Rate. Since GM can remain high even when one user is small, it would be useful to add a plot of the worst user rate and the median user rate for the same simulation to confirm that your GM design indeed lifts the weakest user relative to FC and NOSA.

7. The computational complexity should be analyzed in terms of number of additions and multiplications or in terms of big-Oh notation.

8. Please explain whether the proposed algorithm gives the local or global optimum, even though there is a discussion on no guarantee of global optimality.

9. In Fig. 4(b), how can the GM rate of Q = 4 perform better than that of 16 and infinity ? Perhaps you may need to average over more Monte Carlo simulations.

10. The CSI error model and Fig. 7 are unclear. You define NCPE in (34) but the $x$ axis uses NMSE in dB and the caption says imperfect channel estimation, which is confusing.

References

[REF01] “Multi-Functional RIS for a Multi-Functional System: Integrating Sensing, Communication, and Wireless Power Transfer,” in IEEE Network, doi: 10.1109/MNET.2024.3482571

[REF02] “RIS-Enabled Integrated Sensing and Communication for 6G Systems,” 2024 IEEE Wireless Communications and Networking Conference (WCNC), Dubai, United Arab Emirates, 2024, pp. 1-6, doi: 10.1109/WCNC57260.2024.10571090

Reviewer #2: This manuscript studies an RIS-aided mmWave hybrid beamforming system that adopts an overlapped subarray architecture at the base station to improve spatial flexibility and hardware efficiency. The authors formulate a geometric mean rate maximization problem and develop a two-step BCD framework to optimize a fully digital equivalent precoder together with the RIS reflection phases. A Riemannian manifold-based factorization is applied to decompose the fully digital solution into realizable analog and digital parts under OSA constraints. The proposed initialization and optimization strategy improves convergence and achieves an 18.18 percent objective gain over previous methods. Simulation results show that 3-bit phase control is sufficient to approach continuous phase performance and that OSA can approach the performance of fully connected structures.

However, several issues still need to be resolved in the following:

1. The introduction explains the advantages of RIS and OSA architectures, but the manuscript does not clearly identify the specific fairness or reliability problem that geometric mean maximization uniquely addresses. Since GM rate optimization is the key point to your formulation, this manuscript needs a more concrete description of when and why the GM rate provides a necessary improvement over conventional sum rate objectives.

Please clarify the fairness-related performance gap observed in existing RIS hybrid beamforming methods and provide a concrete scenario, such as blockage-prone or cell-edge users, where GM rate offers distinct advantages.

2. The contribution list is long but does not clearly emphasize what is fundamentally new versus what is incremental. Some contributions are descriptive rather than conceptual.

(1) Please refine the contribution list so that each point states a unique technical advancement rather than a procedural step.

(2) Please explicitly identify what part of the algorithm or framework is new relative to the conference version referenced in the manuscript.

3. The Introduction section covers conventional optimization approaches but does not discuss AI-based precoding or reinforcement learning-based RIS control, which have become major competing directions in this field. Avoiding these methods creates a gap in the contextualization of your contribution, because AI techniques are often applied to the same type of non-convex joint beamforming problems.

(1) Please add a subsection in the introduction or related work that reviews AI-based hybrid beamforming and RIS optimization frameworks, including deep reinforcement learning approaches.

This discussion should highlight the advantages and disadvantages (or limitations) of AI methods relative to your fully model-based optimization framework.

(2) Please include a comparison table summarizing classical optimization, your proposed method, and AI-based methods, highlighting differences in complexity, interpretability, data requirements, and scalability.

(3) Please cite and compare with the following work in your Introduction and Performance Evaluation (experiments):

P. -H. Chou, B. -R. Zheng, W. -J. Huang, W. Saad, Y. Tsao, and R. Y. Chang, "Deep Reinforcement Learning-Based Precoding for Multi-RIS-Aided Multiuser Downlink Systems With Practical Phase Shift," in IEEE Wireless Communications Letters, vol. 14, no. 1, pp. 23-27, Jan. 2025, doi: 10.1109/LWC.2024.3482720.

Since your system involves a single RIS, it is a simpler configuration than a multi-RIS system in the above work. Therefore, it should be included a comparison of performances, algorithmic complexity, fairness behavior, and practicality.

4. The two-step BCD procedure, the RIS phase update, and the Riemannian manifold factorization are mathematically correct but difficult for readers to follow on first reading. Several steps involve complex transformations that would benefit from intuition and a structural overview.

(1) Please provide a high-level flow diagram summarizing the full optimization pipeline, showing the interaction between Q updates, RIS phase updates, and the hybrid decomposition stage.

(2) Please add brief intuitive explanations after major derivation steps to help us understand why each approximation or update rule is mathematically and conceptually reasonable.

5. Since the manuscript claims improved efficiency and faster convergence, such evidence should be quantified.

(1) Please include a runtime or algorithmic complexity comparison to support the claims of computational efficiency.

(2) Please add two additional baselines, including WMMSE-based hybrid beamforming and a DDPG-based RIS control method, to contextualize the performance gains of your method.

6. The manuscript motivates OSA hardware feasibility and low phase resolution, but the practical context of these choices is not illustrated. Since RIS and hybrid architectures face real-world constraints, the relevance of the proposed method would be clearer with concrete examples.

(1) Please describe one or two realistic deployment scenarios where OSA-based RIS hybrid beamforming is beneficial, such as indoor hotspot expansion, factory automation, or blockage mitigation.

(2) Please briefly discuss control overhead, calibration, or signaling requirements for RIS phase updates and OSA analog precoding.

7. Several notation-related issues in the manuscript affect readability.

(1) Please prepare a notation table placed either at the end of the Introduction or at the beginning of the System Model.

The table should include:

(a) All variables, vectors, matrices, and sets

(b) A concise description of their roles

(c) Dimensions where appropriate

(d) Constraints (unit modulus, discrete phase set, manifold space, etc.)

(e) Iteration indices with a clear explanation of which algorithmic stage they belong to

(2) Please unify the superscript and subscript conventions so that iteration indices, user indices, antenna indices, and RIS indices follow a consistent ordering and formatting.

(3) Please ensure each symbol is explicitly defined at its first occurrence and that symbols reused for different purposes (such as μ variations) are renamed or clarified to avoid ambiguity.

8. Comments on the Quality of English.

(1) Sentences are often too long and contain multiple nested clauses, which makes several technical descriptions difficult to follow.

Please break long sentences into shorter units and simplify clause structure to improve clarity, especially in Sections 2 and 3.

(2) Some symbols and technical terms appear before being clearly defined, and several pronouns (“this,” “that,” “these”) refer to ambiguous antecedents.

Please ensure that each variable is explicitly defined at first use and replace vague pronouns with specific referents to reduce ambiguity.

(3) Minor grammatical inconsistencies appear throughout the manuscript, including article usage, subject-verb agreement, and inconsistent compound noun formatting (e.g., “fully digital” vs “fully-digital,” “phase shifter” vs “phase-shifter”).

Please perform a careful language pass to correct these recurring issues and maintain consistent technical terminology.

Reviewer #3: This paper investigated a RIS-aided hybrid beamforming system designed for scalable operation in the millimeter wave channel. The authors proposed a framework that jointly optimizes the linear transmit beamformers and the programmable reflecting coefficients of the RIS elements. Generally, the authors have done an interesting work, however, some parts need to be further improved.

Please see the detailed comments in the attached file.

**********

6. PLOS authors have the option to publish the peer review history of their article (what does this mean?). If published, this will include your full peer review and any attached files.

If you choose “no”, your identity will remain anonymous but your review may still be made public.

**Do you want your identity to be public for this peer review?**  For information about this choice, including consent withdrawal, please see our Privacy Policy.

Reviewer #1: No

Reviewer #2: No

Reviewer #3: No

**********

[NOTE: If reviewer comments were submitted as an attachment file, they will be attached to this email and accessible via the submission site. Please log into your account, locate the manuscript record, and check for the action link "View Attachments". If this link does not appear, there are no attachment files.]

To ensure your figures meet our technical requirements, please review our figure guidelines: https://journals.plos.org/plosone/s/figures

You may also use PLOS’s free figure tool, NAAS, to help you prepare publication quality figures: https://journals.plos.org/plosone/s/figures#loc-tools-for-figure-preparation.

NAAS will assess whether your figures meet our technical requirements by comparing each figure against our figure specifications.

Attachment Submitted filename: Comments to PONE-D-25-56680.pdf

# Author comment: Author response to Decision Letter 1

**DOI:** 10.1371/journal.pone.0354965.r002

6 Mar 2026

Response to the Reviewers

Original Manuscript ID: PONE-D-25-56680

Original Article Title: Scalable RIS-aided Hybrid Beamforming for mmWave Systems enables Multiple Sub-array Architectures: A Geometric Mean Approach

Authors: Hoang M. Tran, Tung V. Dinh, Hoa T. Nguyen, Huy T. Nguyen

Dear Editor and Reviewers,

We appreciate the time you have dedicated to handling our manuscript and for giving us the

opportunity to revise and resubmit it. In this revision, we have made the following updates:

• We have revised and clarified the system setup, along with the application of the considered system.

• We have updated and improved the description of the signal detection process at the

target to make it more readable to readers.

• We have improved the discussion about the non-convexity and challenges in solving

the problem of maximizing the mutual information.

• We have added a discussion on the convergence characteristic and clarified the accuracy

of the result achieved by the proposed algorithm in solving the problem of maximizing

the mutual information.

• We have rechecked and proofread the whole manuscript.

In the following, we provide point-to-point responses to the comments and actions that we

have taken to address those issues. The raised comments and our answers are described in

italic font and roman font, respectively. Unless stated otherwise, all the numbered items (figures, equations, etc.) in this response letter refer to those in the revised manuscript.

Furthermore, in the revised manuscript, the major changed parts, such as updated related

works, more detailed explanations of the proposed system model, new numerical results, and

more insightful discussions have been highlighted in blue color. Other minor corrections and

rearrangements have not been highlighted. Please also note that in this response, we provide

many relevant references to strongly support the correctness of some of our arguments. However, highly correlated references are cited in the revised manuscript.

Further, we would like the Editor to assign our article to the same set of Reviewers for further

review. We do hope that the Editor and Reviewers find our explanations/justifications and

revisions/improvements satisfactory.

Yours sincerely

Attachment Submitted filename: THz_communication__PloS___R1_response.pdf

# Peer review: Decision Letter 1

**DOI:** 10.1371/journal.pone.0354965.r003

**Authors:** Trong-Minh Hoang

15 May 2026

PONE-D-25-56680R1

Scalable RIS-aided Hybrid Beamforming for mmWave Systems enables Multiple Sub-array Architectures: A Geometric Mean Approach

PLOS One

Dear Dr. Nguyen Thanh,

Thank you for submitting your manuscript to PLOS ONE. After careful consideration, we feel that it has merit but does not fully meet PLOS ONE’s publication criteria as it currently stands. Therefore, we invite you to submit a revised version of the manuscript that addresses the points raised during the review process.

Please submit your revised manuscript by Jun 29 2026 11:59PM. If you will need more time than this to complete your revisions, please reply to this message or contact the journal office at plosone@plos.org. When you're ready to submit your revision, log on to https://www.editorialmanager.com/pone/ and select the 'Submissions Needing Revision' folder to locate your manuscript file.

Please include the following items when submitting your revised manuscript:

- A letter that responds to each point raised by the academic editor and reviewer(s). You should upload this letter as a separate file labeled 'Response to Reviewers'.
- A marked-up copy of your manuscript that highlights changes made to the original version. You should upload this as a separate file labeled 'Revised Manuscript with Track Changes'.
- An unmarked version of your revised paper without tracked changes. You should upload this as a separate file labeled 'Manuscript'.

If you would like to make changes to your financial disclosure, please include your updated statement in your cover letter. Guidelines for resubmitting your figure files are available below the reviewer comments at the end of this letter.

If applicable, we recommend that you deposit your laboratory protocols in protocols.io to enhance the reproducibility of your results. Protocols.io assigns your protocol its own identifier (DOI) so that it can be cited independently in the future. For instructions see: https://journals.plos.org/plosone/s/submission-guidelines#loc-laboratory-protocols. Additionally, PLOS ONE offers an option for publishing peer-reviewed Lab Protocol articles, which describe protocols hosted on protocols.io. Read more information on sharing protocols at https://plos.org/protocols?utm_medium=editorial-email&utm_source=authorletters&utm_campaign=protocols.

As the corresponding author, your ORCID iD is verified in the submission system and will appear in the published article. PLOS supports the use of ORCID, and we encourage all coauthors to register for an ORCID iD and use it as well. Please encourage your coauthors to verify their ORCID iD within the submission system before final acceptance, as unverified ORCID iDs will not appear in the published article. *Only*  the individual author can complete the verification step; PLOS staff *cannot*  verify ORCID iDs on behalf of authors.

We look forward to receiving your revised manuscript.

Kind regards,

Trong-Minh Hoang

Academic Editor

PLOS One

Journal Requirements:

If the reviewer comments include a recommendation to cite specific previously published works, please review and evaluate these publications to determine whether they are relevant and should be cited. There is no requirement to cite these works unless the editor has indicated otherwise.

Additional Editor Comments :

Decision: Major revision

The authors should provide more explicit technical explanations in the rebuttal, strengthen the quantitative justification for the geometric mean objective, and clarify the manuscript’s main novelty relative to existing RIS-aided hybrid beamforming methods.

The reviewer also requests broader or better-scoped experimental evaluation, clearer discussion of assumptions and limitations, stronger comparison with analytical and learning-based methods, more rigorous statistical analysis, and full compliance with PLOS data availability requirements.

See the full detailed comments from reviewers.

[Note: HTML markup is below. Please do not edit.]

Reviewers' comments:

Reviewer's Responses to Questions

**Comments to the Author**

1. If the authors have adequately addressed your comments raised in a previous round of review and you feel that this manuscript is now acceptable for publication, you may indicate that here to bypass the “Comments to the Author” section, enter your conflict of interest statement in the “Confidential to Editor” section, and submit your "Accept" recommendation.

Reviewer #1: (No Response)

Reviewer #2: (No Response)

Reviewer #3: All comments have been addressed

**********

2. Is the manuscript technically sound, and do the data support the conclusions?

The manuscript must describe a technically sound piece of scientific research with data that supports the conclusions. Experiments must have been conducted rigorously, with appropriate controls, replication, and sample sizes. The conclusions must be drawn appropriately based on the data presented.

Reviewer #1: (No Response)

Reviewer #2: Partly

Reviewer #3: Yes

**********

3. Has the statistical analysis been performed appropriately and rigorously?

Reviewer #1: (No Response)

Reviewer #2: No

Reviewer #3: Yes

**********

4. Have the authors made all data underlying the findings in their manuscript fully available?

The PLOS Data policy requires authors to make all data underlying the findings described in their manuscript fully available without restriction, with rare exception (please refer to the Data Availability Statement in the manuscript PDF file). The data should be provided as part of the manuscript or its supporting information, or deposited to a public repository. For example, in addition to summary statistics, the data points behind means, medians and variance measures should be available. If there are restrictions on publicly sharing data—e.g. participant privacy or use of data from a third party—those must be specified.

Reviewer #1: (No Response)

Reviewer #2: No

Reviewer #3: No

**********

5. Is the manuscript presented in an intelligible fashion and written in standard English?

PLOS ONE does not copyedit accepted manuscripts, so the language in submitted articles must be clear, correct, and unambiguous. Any typographical or grammatical errors should be corrected at revision, so please note any specific errors here.

Reviewer #1: (No Response)

Reviewer #2: Yes

Reviewer #3: Yes

**********

6. Review Comments to the Author

Please use the space provided to explain your answers to the questions above. You may also include additional comments for the author, including concerns about dual publication, research ethics, or publication ethics. (Please upload your review as an attachment if it exceeds 20,000 characters)

Reviewer #1: The reviewer has no further comments on the manuscript.

Thank for you addressing my previous comments.

Reviewer #2: Several important issues remain only partially addressed:

1. While the authors have provided responses to most reviewer comments, many replies rely on referencing revised sections (e.g., “added in Section X”) without directly explaining the key technical rationale in the rebuttal.

Please provide concise but explicit explanations in the response letter for the core design choices and technical reasoning, rather than relying solely on manuscript revisions.

2. The motivation for adopting the geometric mean (GM) objective has been improved, but remains primarily qualitative without sufficient quantitative validation.

Please further strengthen the motivation by providing quantitative evidence (e.g., fairness metrics, rate distribution comparisons, or performance gaps) to clearly demonstrate the necessity of GM compared to sum-rate or proportional fairness approaches.

3. Although the contribution list has been rewritten, the distinction between fundamental novelty and incremental improvement is still not sufficiently distinguished from existing methods.

Please explicitly clarify:

(1) What is the primary technical novelty

(2) How it differs from existing RIS-aided hybrid beamforming methods at a methodological level

(3) What unique advantage is achieved beyond standard BCD or manifold-based approaches

4. Several reviewer concerns are addressed by stating that they are “outside the scope” (e.g., dynamic environments, robustness, extended performance metrics), without sufficient supporting discussion.

Please provide at least a brief analytical or conceptual discussion to justify these assumptions and clarify how they impact the applicability and generalizability of the proposed method.

5. The experimental evaluation remains limited, focusing primarily on geometric mean rate and convergence behavior, despite the added discussion.

Please either include additional performance evaluations or clearly limit the scope of the conclusions.

6. The positioning of the proposed method with respect to existing literature can be further strengthened, particularly in terms of deeper comparative analysis with both analytical and learning-based approaches.

Please enhance the discussion to more clearly highlight the advantages and limitations of the proposed framework.

Reviewer #3: The authors have addressed my concerns, please finally check the repeated references, such as [11]-[13] and [15], before uploading the final files.

**********

7. PLOS authors have the option to publish the peer review history of their article (what does this mean?). If published, this will include your full peer review and any attached files.

If you choose “no”, your identity will remain anonymous but your review may still be made public.

**Do you want your identity to be public for this peer review?**  For information about this choice, including consent withdrawal, please see our Privacy Policy.

Reviewer #1: No

Reviewer #2: No

Reviewer #3: No

**********

[NOTE: If reviewer comments were submitted as an attachment file, they will be attached to this email and accessible via the submission site. Please log into your account, locate the manuscript record, and check for the action link "View Attachments". If this link does not appear, there are no attachment files.]

To ensure your figures meet our technical requirements, please review our figure guidelines: https://journals.plos.org/plosone/s/figures

You may also use PLOS’s free figure tool, NAAS, to help you prepare publication quality figures: https://journals.plos.org/plosone/s/figures#loc-tools-for-figure-preparation.

NAAS will assess whether your figures meet our technical requirements by comparing each figure against our figure specifications.

# Author comment: Author response to Decision Letter 2

**DOI:** 10.1371/journal.pone.0354965.r004

27 Jun 2026

Response to the Reviewers

Original Manuscript ID: PONE-D-25-56680

Original Article Title: Scalable RIS-aided Hybrid Beamforming for mmWave Systems enables Multiple Sub-array Architectures: A Geometric Mean Approach

Authors: Hoang M. Tran, Tung V. Dinh, Hoa T. Nguyen, Huy T. Nguyen

Dear Editor and Reviewers,

We appreciate the time you have dedicated to handling our manuscript and for giving us the opportunity to revise and resubmit it. In this revision, we have made the following updates:

• We have revised and clarified the system setup, along with the application of the considered system.

• We have updated and improved the description of the signal detection process at the target to make it more readable to readers.

• We have improved the discussion about the non-convexity and challenges in solving the problem of maximizing the mutual information.

• We have added a discussion on the convergence characteristic and clarified the accuracy of the result achieved by the proposed algorithm in solving the problem of maximizing

the mutual information.

• We have rechecked and proofread the whole manuscript.

In the following, we provide point-to-point responses to the comments and actions that we have taken to address those issues. The raised comments and our answers are described in italic font and roman font, respectively. Unless stated otherwise, all the numbered items (figures, equations, etc.) in this response letter refer to those in the revised manuscript.

Furthermore, in the revised manuscript, the major changed parts, such as updated related works, more detailed explanations of the proposed system model, new numerical results, and more insightful discussions have been highlighted in blue color. Other minor corrections and rearrangements have not been highlighted. Please also note that in this response, we provide many relevant references to strongly support the correctness of some of our arguments. However, highly correlated references are cited in the revised manuscript.

Further, we would like the Editor to assign our article to the same set of Reviewers for further review. We do hope that the Editor and Reviewers find our explanations/justifications and revisions/improvements satisfactory.

Yours sincerely,

Attachment Submitted filename: ReponseLetter_R2 (PloS).pdf

# Editor report: Decision Letter 2

**DOI:** 10.1371/journal.pone.0354965.r005

**Authors:** Trong-Minh Hoang

5 Jul 2026

PONE-D-25-56680R2

Scalable RIS-aided Hybrid Beamforming for mmWave Systems enables Multiple Sub-array Architectures: A Geometric Mean Approach

PLOS One

Dear Dr. Nguyen Thanh,

Thank you for submitting your manuscript to PLOS ONE. After careful consideration, we feel that it has merit but does not fully meet PLOS ONE’s publication criteria as it currently stands. Therefore, we invite you to submit a revised version of the manuscript that addresses the points raised during the review process.

After careful consideration of the revised manuscript and the reviewers’ comments, I invite you to submit a further revised version of the manuscript. Although two reviewers consider the manuscript acceptable and acknowledge that their previous concerns have been addressed, one reviewer has identified several remaining issues that must be resolved before the manuscript can be considered for publication. In addition, concerns remain regarding statistical rigor and data availability, which are part of PLOS ONE’s publication criteria.

The following revisions are required. First, the response letter should be strengthened. The authors should not only refer to revised manuscript sections, but should also provide concise and explicit explanations of the main technical rationale, design choices, and methodological decisions. Second, the motivation for using the geometric mean objective should be supported more quantitatively. The revised manuscript should include additional evidence, such as fairness metrics, rate distribution comparisons, or performance-gap analysis, to clarify why the geometric mean objective is necessary compared with sum-rate or proportional fairness objectives.

Third, the authors should more clearly distinguish the primary technical contribution from incremental improvements over existing RIS-aided hybrid beamforming methods. In particular, the revision should explicitly explain the main methodological novelty, how it differs from standard BCD-based, manifold-optimization-based, analytical, or learning-based approaches, and what specific advantage is obtained by the proposed framework.

Fourth, assumptions that were previously described as being outside the scope of the paper, such as dynamic environments, robustness, and extended performance metrics, should be discussed more carefully. The authors do not necessarily need to solve all of these issues in the present work, but they should provide a clear analytical or conceptual justification of the assumptions and explain how these limitations affect the applicability and generalizability of the proposed method.

Fifth, the experimental evaluation should be strengthened or the conclusions should be narrowed accordingly. If additional performance evaluations are feasible, the authors should include them. If not, the manuscript should explicitly limit its claims to the evaluation settings that have actually been tested. Sixth, the positioning of the proposed method within the existing literature should be improved through a deeper comparative discussion with representative analytical and learning-based methods.

Finally, the authors must address the remaining data availability and reproducibility concerns. Please ensure that all data, code, simulation parameters, or sufficient information needed to reproduce the results are made available in accordance with PLOS ONE’s data policy, unless a valid restriction applies. The authors should also carefully check the reference list and remove or correct repeated references, including the repeated entries noted by the reviewer.

Please submit a detailed point-by-point response to all remaining reviewer and editor comments, a marked-up version of the revised manuscript showing all changes, and a clean version of the manuscript. The revised submission will undergo further editorial assessment and may be returned to the reviewers if necessary.

Please submit your revised manuscript by Aug 17 2026 11:59PM. If you will need more time than this to complete your revisions, please reply to this message or contact the journal office at plosone@plos.org. When you're ready to submit your revision, log on to https://www.editorialmanager.com/pone/ and select the 'Submissions Needing Revision' folder to locate your manuscript file.

Please include the following items when submitting your revised manuscript:

- A letter that responds to each point raised by the academic editor and reviewer(s). You should upload this letter as a separate file labeled 'Response to Reviewers'.
- A marked-up copy of your manuscript that highlights changes made to the original version. You should upload this as a separate file labeled 'Revised Manuscript with Track Changes'.
- An unmarked version of your revised paper without tracked changes. You should upload this as a separate file labeled 'Manuscript'.

If you would like to make changes to your financial disclosure, please include your updated statement in your cover letter. Guidelines for resubmitting your figure files are available below the reviewer comments at the end of this letter.

If applicable, we recommend that you deposit your laboratory protocols in protocols.io to enhance the reproducibility of your results. Protocols.io assigns your protocol its own identifier (DOI) so that it can be cited independently in the future. For instructions see: https://journals.plos.org/plosone/s/submission-guidelines#loc-laboratory-protocols. Additionally, PLOS ONE offers an option for publishing peer-reviewed Lab Protocol articles, which describe protocols hosted on protocols.io. Read more information on sharing protocols at https://plos.org/protocols?utm_medium=editorial-email&utm_source=authorletters&utm_campaign=protocols.

As the corresponding author, your ORCID iD is verified in the submission system and will appear in the published article. PLOS supports the use of ORCID, and we encourage all coauthors to register for an ORCID iD and use it as well. Please encourage your coauthors to verify their ORCID iD within the submission system before final acceptance, as unverified ORCID iDs will not appear in the published article. *Only*  the individual author can complete the verification step; PLOS staff *cannot*  verify ORCID iDs on behalf of authors.

We look forward to receiving your revised manuscript.

Kind regards,

Trong-Minh Hoang

Academic Editor

PLOS ONE

Journal Requirements:

If the reviewer comments include a recommendation to cite specific previously published works, please review and evaluate these publications to determine whether they are relevant and should be cited. There is no requirement to cite these works unless the editor has indicated otherwise.

Please review your reference list to ensure that it is complete and correct. If you have cited papers that have been retracted, please include the rationale for doing so in the manuscript text, or remove these references and replace them with relevant current references. Any changes to the reference list should be mentioned in the rebuttal letter that accompanies your revised manuscript. If you need to cite a retracted article, indicate the article’s retracted status in the References list and also include a citation and full reference for the retraction notice.

Additional Editor Comments:

Although two reviewers now recommend acceptance, I invite the authors to submit a further major revision because several issues relevant to PLOS ONE’s publication criteria remain unresolved. In particular, one reviewer still finds the manuscript only partly technically sound, indicates that the statistical analysis is not sufficiently rigorous, and raises concerns about data availability. Another reviewer also notes data availability concerns and asks the authors to check repeated references before final submission.

The authors are required to strengthen the response letter by providing explicit technical explanations rather than only referring to revised sections; provide stronger quantitative justification for the geometric mean objective; clarify the primary methodological contribution relative to existing RIS-aided hybrid beamforming approaches, including BCD-based, manifold-based, analytical, and learning-based methods; discuss the assumptions and limitations related to dynamic environments, robustness, and extended performance metrics; strengthen the experimental evaluation or narrow the conclusions accordingly; improve the comparative positioning within the literature; and address data availability and reproducibility in accordance with PLOS ONE policy. The authors should also correct repeated references and provide a detailed point-by-point response, a tracked-changes manuscript, and a clean manuscript.

[Note: HTML markup is below. Please do not edit.]

[NOTE: If reviewer comments were submitted as an attachment file, they will be attached to this email and accessible via the submission site. Please log into your account, locate the manuscript record, and check for the action link "View Attachments". If this link does not appear, there are no attachment files.]

To ensure your figures meet our technical requirements, please review our figure guidelines: https://journals.plos.org/plosone/s/figures

You may also use PLOS’s free figure tool, NAAS, to help you prepare publication quality figures: https://journals.plos.org/plosone/s/figures#loc-tools-for-figure-preparation.

NAAS will assess whether your figures meet our technical requirements by comparing each figure against our figure specifications.

# Author comment: Author response to Decision Letter 3

**DOI:** 10.1371/journal.pone.0354965.r006

13 Jul 2026

Original Manuscript ID: PONE-D-25-56680

Original Article Title: Scalable RIS-aided Hybrid Beamforming for mmWave Systems enables Multiple Sub-array Architectures: A Geometric Mean Approach

Authors: Hoang M. Tran, Tung V. Dinh, Hoa T. Nguyen, Huy T. Nguyen

Dear Editor and Reviewers,

We appreciate the time you have dedicated to handling our manuscript and for giving us the opportunity to revise and resubmit it. In this revision, we have made the following updates:

• We have revised and clarified the system setup, along with the application of the considered system.

• We have updated and improved the description of the signal detection process at the target to make it more readable to readers.

• We have improved the discussion about the non-convexity and challenges in solving the problem of maximizing the mutual information.

• We have added a discussion on the convergence characteristic and clarified the accuracy of the result achieved by the proposed algorithm in solving the problem of maximizing the mutual information.

• We have rechecked and proofread the whole manuscript.

In the following, we provide point-to-point responses to the comments and actions that we have taken to address those issues. The raised comments and our answers are described in italic font and roman font, respectively. Unless stated otherwise, all the numbered items (figures, equations, etc.) in this response letter refer to those in the revised manuscript.

Furthermore, in the revised manuscript, the major changed parts, such as updated related works, more detailed explanations of the proposed system model, new numerical results, and more insightful discussions have been highlighted in blue color. Other minor corrections and rearrangements have not been highlighted. Please also note that in this response, we provide many relevant references to strongly support the correctness of some of our arguments. However, highly correlated references are cited in the revised manuscript.

Further, we would like the Editor to assign our article to the same set of Reviewers for further review. We do hope that the Editor and Reviewers find our explanations/justifications and revisions/improvements satisfactory.

Yours sincerely,

Attachment Submitted filename: ReponseLetter_R3 (PloS).pdf

# Editor report: Decision Letter 3

**DOI:** 10.1371/journal.pone.0354965.r007

**Authors:** Trong-Minh Hoang

15 Jul 2026

Scalable RIS-aided Hybrid Beamforming for mmWave Systems enables Multiple Sub-array Architectures: A Geometric Mean Approach

PONE-D-25-56680R3

Dear Dr. Nguyen Thanh,

We’re pleased to inform you that your manuscript has been judged scientifically suitable for publication and will be formally accepted for publication once it meets all outstanding technical requirements.

Within one week, you’ll receive an e-mail detailing the required amendments. When these have been addressed, you’ll receive a formal acceptance letter and your manuscript will be scheduled for publication.

An invoice will be generated when your article is formally accepted. Please note, if your institution has a publishing partnership with PLOS and your article meets the relevant criteria, all or part of your publication costs will be covered. Please make sure your user information is up-to-date by logging into Editorial Manager at Editorial Manager® and clicking the ‘Update My Information' link at the top of the page. For questions related to billing, please contact billing support.

If your institution or institutions have a press office, please notify them about your upcoming paper to help maximize its impact. If they’ll be preparing press materials, please inform our press team as soon as possible -- no later than 48 hours after receiving the formal acceptance. Your manuscript will remain under strict press embargo until 2 pm Eastern Time on the date of publication. For more information, please contact onepress@plos.org.

Kind regards,

Trong-Minh Hoang

Academic Editor

PLOS One

Additional Editor Comments (optional):

Date: July 15, 2026

Manuscript ID: PONE-D-25-56680R2

Title: Scalable RIS-aided Hybrid Beamforming for mmWave Systems enables Multiple Sub-array Architectures: A Geometric Mean Approach

Authors: Hoang M. Tran, Tung V. Dinh, Hoa T. Nguyen, Huy T. Nguyen

Dear Dr. Nguyen Thanh and Co-authors,

On behalf of the Editorial Board of PLOS ONE, I am pleased to inform you that your manuscript has been accepted for publication, subject to the submission of final production files as described below.

After careful consideration of your revised manuscript (R2) and your detailed point-by-point response to all reviewer and editor comments, I am satisfied that the raised concerns have been adequately addressed. The revisions demonstrate a meaningful improvement in technical clarity, methodological justification, experimental evaluation, and compliance with PLOS ONE's publication criteria.

Required Final Submission Items

To proceed to production, please submit the following items through the Editorial Manager system at https://www.editorialmanager.com/pone/:

Final clean manuscript (labeled "Manuscript") — no tracked changes, no highlighted text, fully proofread.

Final figure files — high-resolution, meeting PLOS ONE figure specifications (https://journals.plos.org/plosone/s/figures).

Data availability statement — confirming that all simulation parameters, datasets, and/or code sufficient to reproduce the reported results are publicly available or accessible upon reasonable request, in accordance with PLOS ONE data policy.

Corrected reference list — ensuring all repeated or duplicate references have been removed and the reference list is complete and accurate.

ORCID verification — please ensure all co-authors have verified their ORCID iD within the submission system prior to final acceptance. Unverified ORCID iDs will not appear in the published article.

Note: If any data, code, or simulation materials are subject to restrictions, please provide a clear and explicit justification in the data availability statement.

We look forward to receiving your final submission and to publishing your work in PLOS ONE.

Yours sincerely,

Trong-Minh Hoang

Academic Editor

PLOS ONE

# Editor report: Acceptance letter

**DOI:** 10.1371/journal.pone.0354965.r008

**Authors:** Trong-Minh Hoang

PONE-D-25-56680R3

PLOS One

Dear Dr. Nguyen Thanh,

I'm pleased to inform you that your manuscript has been deemed suitable for publication in PLOS One. Congratulations! Your manuscript is now being handed over to our production team.

At this stage, our production department will prepare your paper for publication. This includes ensuring the following:

* All references, tables, and figures are properly cited

* All relevant supporting information is included in the manuscript submission,

* There are no issues that prevent the paper from being properly typeset

You will receive further instructions from the production team, including instructions on how to review your proof when it is ready. Please keep in mind that we are working through a large volume of accepted articles, so please give us a few days to review your paper and let you know the next and final steps.

Lastly, if your institution or institutions have a press office, please let them know about your upcoming paper now to help maximize its impact. If they'll be preparing press materials, please inform our press team within the next 48 hours. Your manuscript will remain under strict press embargo until 2 pm Eastern Time on the date of publication. For more information, please contact onepress@plos.org.

You will receive an invoice from PLOS for your publication fee after your manuscript has reached the completed accept phase. If you receive an email requesting payment before acceptance or for any other service, this may be a phishing scheme. Learn how to identify phishing emails and protect your accounts at https://explore.plos.org/phishing.

If we can help with anything else, please email us at customercare@plos.org.

Thank you for submitting your work to PLOS ONE and supporting open access.

Kind regards,

PLOS ONE Editorial Office Staff

on behalf of

Dr. Trong-Minh Hoang

Academic Editor

PLOS One
